# High-throughput ligand diversification to discover chemical inducers of proximity

**DOI:** 10.1038/s41589-025-02137-2

**Published:** 2026-02-16

**Authors:** James B. Shaum, Miquel Muñoz i Ordoño, Erica A. Steen, Daniela V. Wenge, Hakyung Cheong, Jordan Janowski, Moritz Hunkeler, Eric M. Bilotta, Zoe J. Rutter, Paige A. Barta, Abby M. Thornhill, Natalia Milosevich, Lauren M. Hargis, Timothy R. Bishop, Trever R. Carter, Bryce da Camara, Matthias Hinterndorfer, Lucas Dada, Wen-Ji He, Fabian Offensperger, Hirotake Furihata, Sydney R. Schweber, Charlie Hatton, Yanhe Wen, Benjamin F. Cravatt, Keary M. Engle, Katherine A. Donovan, Bruno Melillo, Seiya Kitamura, Alessio Ciulli, Scott A. Armstrong, Eric S. Fischer, Georg E. Winter, Michael A. Erb

**Affiliations:** 1 Department of Chemistry, The Scripps Research Institute, La Jolla, CA USA; 2 CeMM Research Center for Molecular Medicine of the Austrian Academy of Sciences, Vienna, Austria; 3 AITHYRA Research Institute for Biomedical Artificial Intelligence of the Austrian Academy of Sciences, Vienna, Austria; 4 Division of Hematology/Oncology, Department of Pediatric Oncology, Dana-Farber Cancer Institute, Boston Children’s Hospital, and Harvard Medical School, Boston, MA USA; 5 Department of Cancer Biology, Dana-Farber Cancer Institute, Boston, MA USA; 6 Department of Biological Chemistry and Molecular Pharmacology, Harvard Medical School, Boston, MA USA; 7 Centre for Targeted Protein Degradation, School of Life Sciences, University of Dundee, Dundee, UK; 8 Department of Biochemistry, Albert Einstein College of Medicine, Bronx, New York USA

**Keywords:** Screening, Small molecules, Chemical modification, Structural biology

## Abstract

Chemical inducers of proximity (CIPs) stabilize biomolecular interactions, often causing an emergent rewiring of cellular biochemistry. While the discovery of heterobifunctional CIPs is expedited by rational design strategies, molecular glues have relied predominantly on serendipity. We hypothesized that preexisting ligands could be systematically decorated with chemical modifications to discover compounds that recruit proteins to a composite protein–ligand interface. Using sulfur(VI) fluoride exchange-based high-throughput chemistry (HTC) to install 3,163 structurally diverse building blocks onto ENL (eleven-nineteen leukemia) and BRD4 (bromodomain-containing protein 4) ligands, we screened each analog for degrader activity. This revealed dHTC1, an ENL degrader that recruits CRL4^CRBN^ complex through an extended interface of protein–protein contacts and only engages CRBN after pre-forming the ENL:dHTC1 complex. We also identified dHTC3, a molecular glue that selectively dimerizes BRD4 bromodomain 1 to SCF^FBXO3^, an E3 ligase not previously accessible for chemical rewiring. Altogether, this study introduces HTC as a facile tool to discover new CIPs and new effectors for proximity pharmacology.

**Figure:**
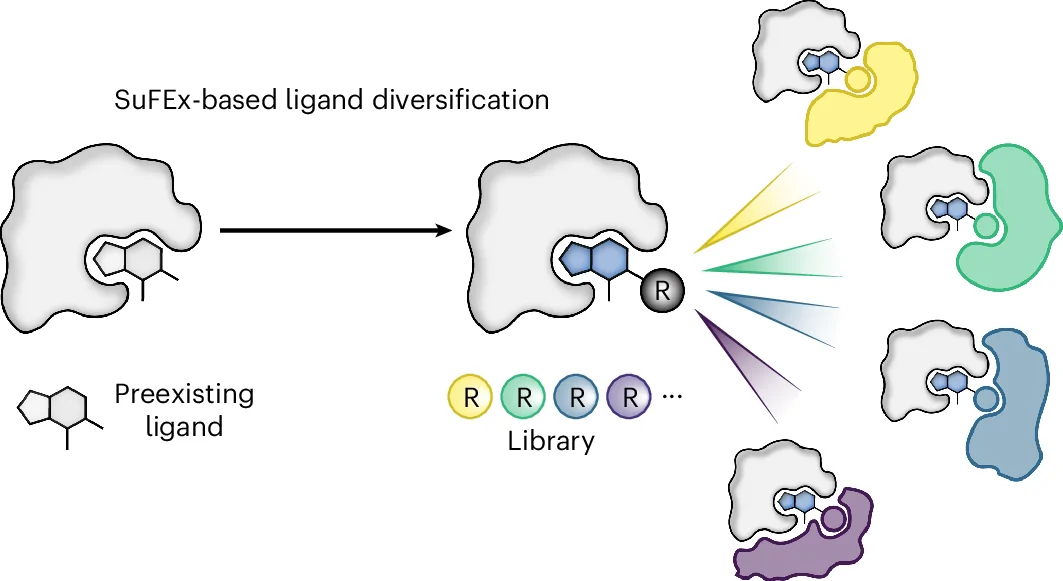

## Abstract (web summary)

Molecular glue degraders have consistently been discovered retrospectively, despite their increasing importance. Herein, a high-throughput approach is described that modifies existing ligands into molecular glue degraders.

## Main

Chemical inducers of proximity (CIPs) include bifunctional small molecules and molecular glues that induce proximity between two targets, rewiring biological circuits in a manner that is difficult to achieve with other pharmacological approaches^1,2^. The first CIPs were discovered serendipitously by studying the mechanisms of action of natural product immunosuppressants^3,4^; however, but heterobifunctional chemistries, exemplified by proteolysis-targeting chimeras (PROTACs), have made it possible to design CIPs prospectively^5,6^. Molecular glues function as CIPs without needing to bind the target and effector independently; instead, they can form a composite protein–ligand surface that cooperatively stabilizes an interfacial protein–protein interaction^7^. For example, thalidomide analogs (or immunomodulatory imide drugs, IMiDs)—anticancer agents that function through this mechanism—resurface the E3 substrate receptor, Cereblon (CRBN), to degrade unligandable neosubstrates^8–17^. These drugs highlight the potential for molecular glues to address difficult classes of drug targets, yet prospective approaches to discover molecular glues remain limited.

Lacking modular design principles like bifunctional CIPs, most molecular glues have been discovered through retrospective mechanism-of-action studies^1,2^. We considered it instructive that several recently discovered molecular glues possess close structural analogs that bind the same target but do not function as glues^18–25^. In several examples, CIP behavior can be attributed to subtle structural differences that project outward toward the solvent, forming a composite protein–ligand surface that can stabilize an interfacial interaction^19,20,25^. However, because of the sporadic and primarily serendipitous nature of these discoveries, it is unclear how often structural alterations can confer glue-type activity to a ligand and whether these alterations can be identified prospectively for a preselected target. To address this, we coupled a high-throughput approach for ligand diversification to a phenotypic readout of neomorphic pharmacological behavior—targeted protein degradation—enabling the prospective discovery of structural alterations that convert ligands into CIPs.

## Results

### High-throughput diversification converts ligand to degraders

**Fig. 1:**
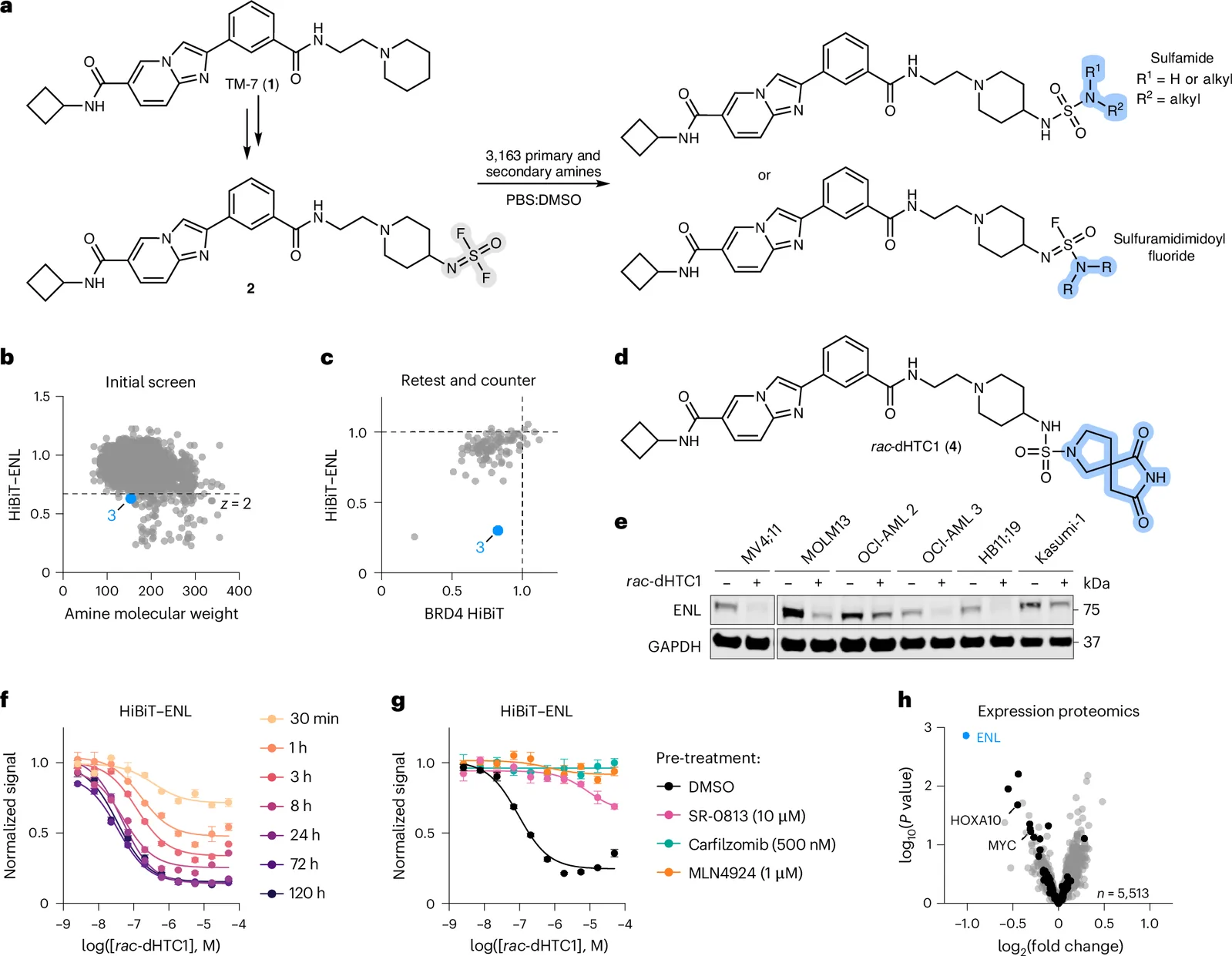
Prospective discovery of *rac*-dHTC1 by HTC diversification. **a**, TM-7 (**1**) with an iminosulfur oxydifluoride SuFEx handle (**2**) was used for the parallel synthesis of 3,163 analogs. **b**, Crude reaction products were screened for ENL degradation (5 µM, 24 h) in MV4;11 cells expressing HiBiT–ENL. HiBiT luminescence normalized to DMSO–PBS vehicle-control-treated cells (single independent experiment). The molecular weight is shown for the free amine coupling partner. Dashed line indicates 2 standard deviations below the mean. **c**, Resynthesized hits were rescreened by HiBiT–ENL and counterscreened against BRD4–HiBiT (*n* = 3 biological replicates). HiBiT luminescence normalized to DMSO–PBS vehicle-control-treated cells. Dashed lines indicate average of DMSO-treated cells. **d**, Chemical structure of *rac*-dHTC1 (**4**). **e**, Immunoblot of ENL in AML cell lines treated with purified *rac*-dHTC1 (10 µM, 24 h) or DMSO vehicle control. Data are representative of two independent experiments. **f**, Time-dependent and dose-responsive loss of HiBiT–ENL signal in MV4;11 cells treated with *rac*-dHTC1. Luminescence is normalized to the DMSO vehicle control (mean ± s.e.m.; *n* = 4 biological replicates). **g**, Dose-responsive effects of *rac*-dHTC1 (6 h) in MV4;11 cells following indicated pretreatments (1 h). HiBiT–ENL luminescence normalized to DMSO vehicle control (mean ± s.e.m.; *n* = 4 biological replicates). Data in Extended Data Fig. 2e were taken from the same experiment; DMSO pretreatment was repeated in both panels. **h**, Quantitative TMT-based expression proteomics analysis of MV4;11 cells treated with *rac*-dHTC1 (1 µM, 16 h) or DMSO vehicle control (5,513 proteins with >2 peptides). Data were filtered using DTAselect 2.0 within IP2. *P* values were calculated using a two-tailed Student’s *t*-test (*n* = 3 biological replicates). Black dots indicate ENL target genes. Source data

**Extended Data Fig. 1:**
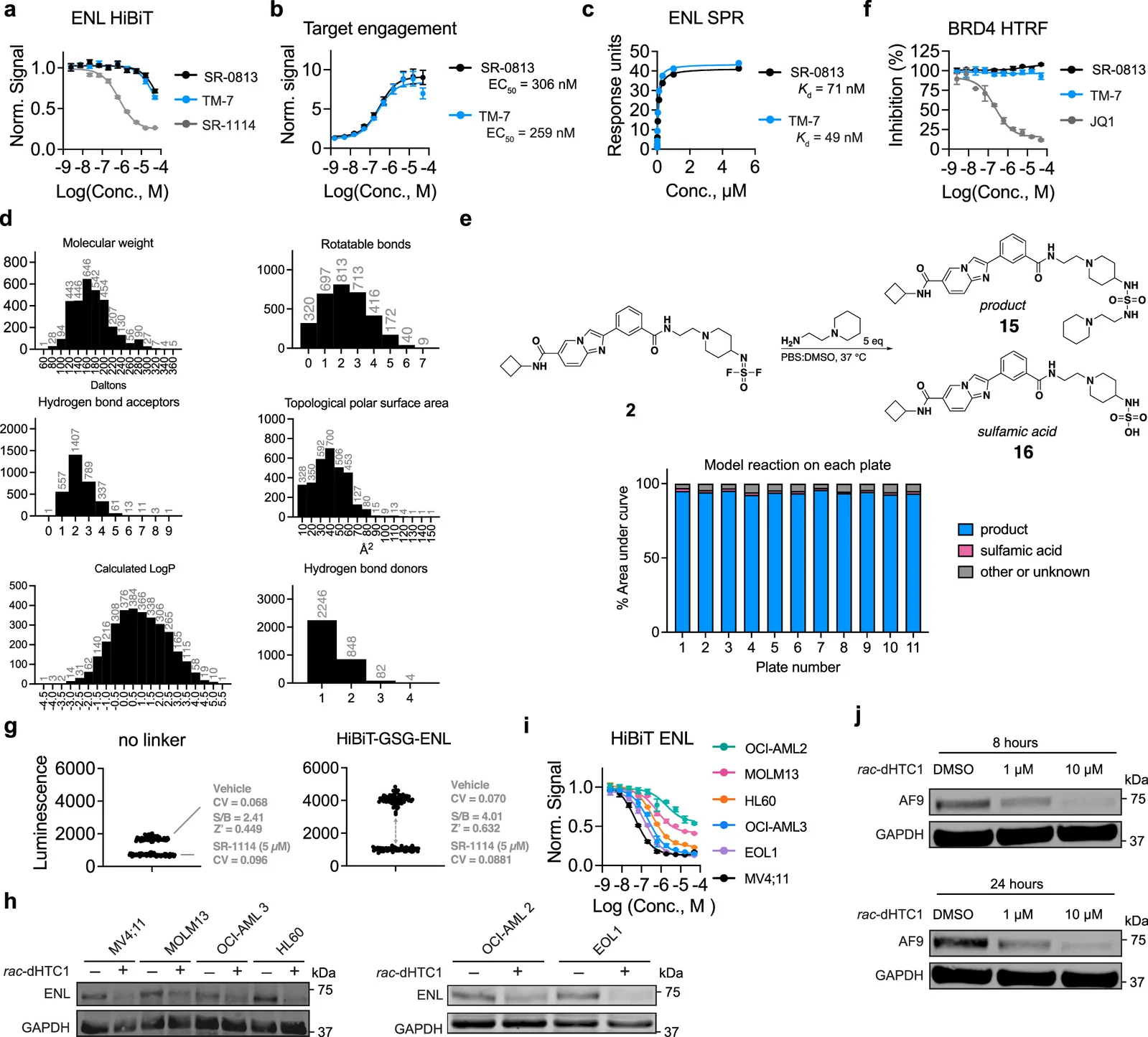
Discovery and characterization of dHTC1. **a**, MV4;11 cells expressing HiBiT-ENL were treated with the indicated compounds for 24 h. HiBiT luminescence signal is normalized to DMSO-treated cells. **b**, Intracellular engagement of ENL YEATS by TM-7 and SR-0813. **c**, ENL YEATS affinity determination using SPR (mean of 2 technical replicates). Same experiment as Extended Data Fig. 9d (TM-7 and SR-0813 are repeated). **d**, Frequency histograms of key physicochemical properties for the library of amine building blocks used in high-throughput derivatization. **e**, Schematic of a model reaction run on each library plate, and the corresponding relative yields (measured as percent integration of total). **f**, BRD4 engagement *in vitro*, as measured by HTRF competitive histone peptide displacement assay (mean of 3 biological replicates ± S.E.M.). **g**, MV4;11 cells were engineered to stably express an amino (N) terminal HiBiT ENL tag. Shown is the raw HiBiT luminescence signal for cells (20,000 cells in 20 µL of media) treated with vehicle control or SR-1114 (5 µM) for 24 h, demonstrating signal window. Left demonstrates cells bearing a direct fusion of the HiBiT tag to ENL. Right demonstrates cells with a flexible GSG linker. **h**, Immunoblot analysis of ENL in a panel of AML cell lines treated with *rac*-dHTC1 (10 µM) or DMSO control (24 h). Representative of 2 independent replicates. **i**, ENL-HiBiT expressing AML cell lines treated with *rac*-dHTC1 for 24 hours. Signal is normalized to DMSO treated cells. **j**, Immunoblot analysis of AF9 levels in Jurkat cells treated with *rac*-dHTC1 or DMSO control. Single independent experiment. **a,b,i** mean, 4 biological replicates ± S.E.M. Source data

To establish an initial proof of concept, we selected SR-0813, a ligand we previously discovered that binds to the YEATS domain of the leukemia target and transcriptional coactivator eleven-nineteen leukemia (ENL)^26^. As SR-0813 does not affect ENL stability (Extended Data Fig. 1a), we reasoned that high-throughput diversification could be coupled to cellular ENL degradation screens to identify structural modifications that convert it into a CIP. To test this, we used sulfur(VI) fluoride exchange (SuFEx)-based ‘click chemistry’ transformations, in which an iminosulfur oxydifluoride group reacts with primary and secondary aliphatic amines to completion in DMSO–PBS mixtures, forming sulfamides or sulfuramidimidoyl fluorides (Fig. 1a)^26–30^. These mild, biocompatible reaction conditions allow crude products to be tested directly in living cells, thus marrying high-throughput chemistry (HTC) with miniaturized cell-based screens^26,29,30^.

We first furnished TM-7 (**1**), a synthetically simplified analog of SR-0813 (Fig. 1a and Extended Data Fig. 1b,c), with an iminosulfur oxydifluoride SuFEx hub to afford TM-7 difluoride (**2**) and sourced a library of 3,163 commercially available amine building blocks encompassing diverse chemical structures, molecular weights and physiochemical properties (Extended Data Fig. 1d and Supplementary Table 1). We then synthesized more than 3,000 TM-7 analogs in parallel and screened each crude product for degradation of HiBiT–ENL (Fig. 1a,b, Extended Data Fig. 1e and Supplementary Table 2)^31,32^. A total of 93 hits scoring more than 2 s.d. below the mean were promptly resynthesized, retested for ENL degradation in a newly optimized MV4;11 HiBiT–ENL cell line and counterscreened against MV4;11 cells expressing bromodomain-containing protein 4 (BRD4)–HiBiT to remove false positives, as TM-7 itself does not bind BRD4 (Fig. 1c and Extended Data Fig. 1f,g). One hit, the crude product of TM-7 difluoride and a pyrrolidine spirosuccinimide building block JS-1 (**3**), selectively decreased HiBiT–ENL signal (Fig. 1c,d and Supplementary Table 3). It was resynthesized and purified to enable further validation, yielding *rac*-dHTC1 (**4**) as a 1:1 mix of enantiomers (Supplementary Methods and Fig. 1d).

Using a panel of acute myeloid leukemia (AML) cell lines, degradation of native ENL was confirmed by immunoblot (Fig. 1e and Extended Data Fig. 1h) and dose-responsive degradation was assessed by HiBiT, determining half-maximal degradation concentration (DC_50_) values ranging from 50 nM to 1.85 µM and maximal degradation (*D*_max_) ranging from 91% to 58% (Extended Data Fig. 1i and Supplementary Fig. 1a). In MV4;11 cells, degradation was observed within 30 min, maximized after 24 h and sustained for at least 5 days (Fig. 1f). Pretreatment with a saturating concentration of the ENL/AF9 YEATS ligand, SR-0813, prevented loss of HiBiT–ENL signal upon *rac*-dHTC1 treatment (Fig. 1g), which established that on-target engagement of ENL is necessary for its activity. Furthermore, loss of HiBiT–ENL signal could also be rescued by the neddylation inhibitor, MLN4924, and the proteasome inhibitor, carfilzomib (Fig. 1g), indicating that *rac*-dHTC1 prompts proteasome-dependent degradation of ENL through a Cullin–RING ligase (CRL) complex. We used an expression proteomics analysis to evaluate the selectivity of *rac*-dHTC1 in MV4;11 cells, finding that it exclusively degrades ENL while also decreasing the abundance of proteins encoded by transcriptional ENL target genes (for example, *HOXA10* and *MYC*) (Fig. 1h and Supplementary Table 4), a known effect of ENL degradation in MV4;11 cells^26^. AF9, a paralog of ENL that is also bound by this chemical series^26^, was not detected by proteomics; however, an immunoblot analysis confirmed that it can also be degraded by dHTC1 (Extended Data Fig. 1j).

### ENL degradation by dHTC1 proceeds through CRL4^CRBN^

**Fig. 2:**
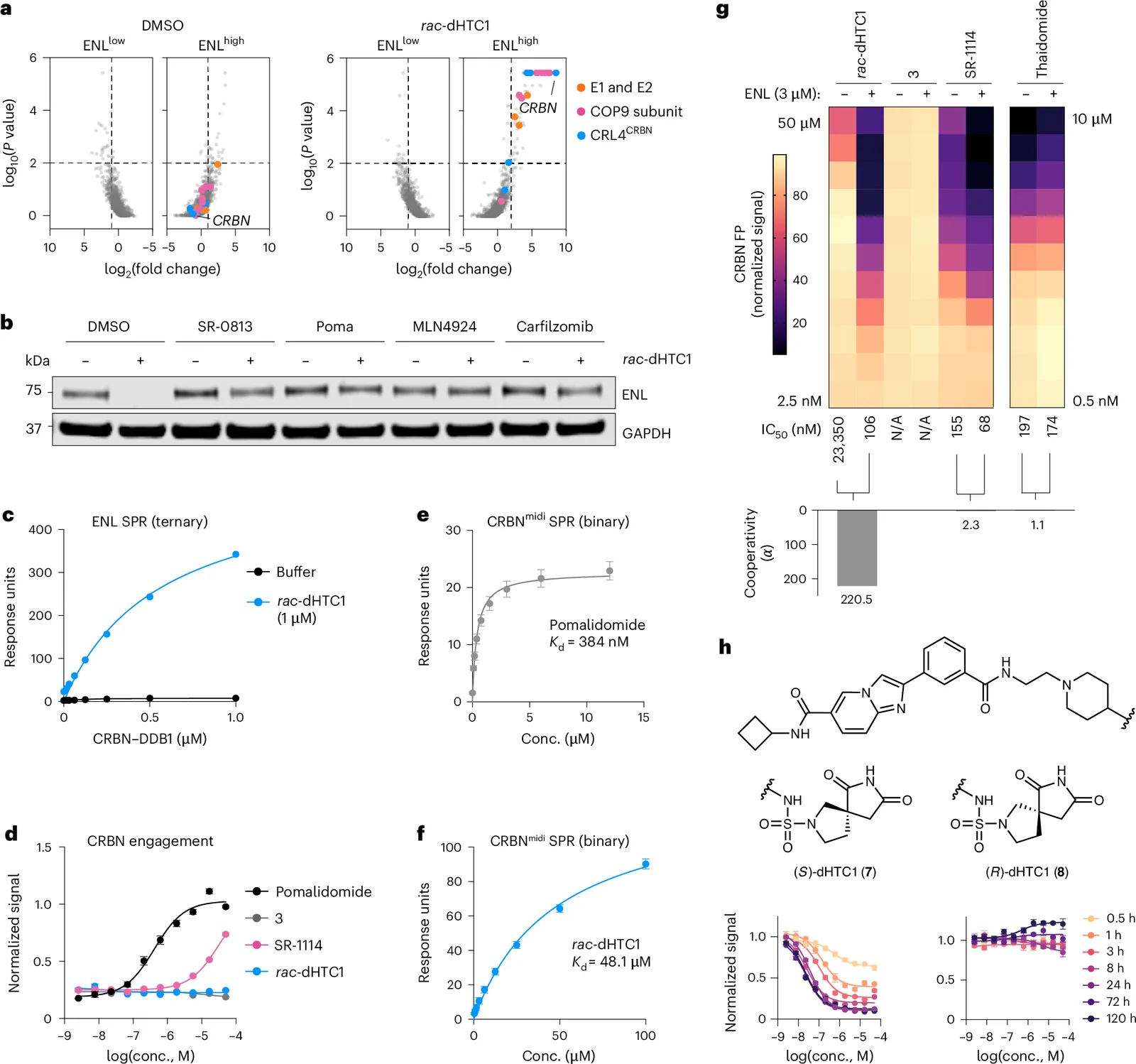
ENL degradation by *rac*-dHTC1 proceeds through CRL4^CRBN^ despite minimal CRBN affinity. **a**, FACS-based CRISPR screens for UPS components affecting ENL stability in KBM7 reporter cells (ENL–BFP–P2A–mCherry) treated with DMSO or *rac*-dHTC1 (10 µM) for 8 h (6 sgRNAs targeting 1,301 UPS-associated genes). Gene-level fold changes and *P* values were determined by one-sided MAGeCK analysis^114^. **b**, Immunoblot analysis of WT treated with *rac*-dHTC1 (10 µM, 6 h) or DMSO vehicle control. Data are representative of two independent experiments. **c**, SPR analysis of CRBN–DDB1 binding to immobilized ENL YEATS in the presence of *rac*-dHTC1 or buffer control (mean; *n* = 2 technical replicates). The same experiment is presented in Extended Data Fig. 3g; *rac*-dHTC1 treatment was repeated in both panels. **d**, Rescue of dBET6-mediated BRD4 degradation to assess CRBN target engagement. MV4;11 cells expressing BRD4–HiBiT were pretreated with compounds in a dose–response series for 2 h before a 1-h treatment with dBET6 (500 nM). HiBiT luminescence signal is normalized to DMSO-treated cells (mean ± s.e.m.; *n* = 3 biological replicates). Data for pomalidomide are repeated in Extended Data Fig. 4e, which originated from the same experiment. Conc., concentration. **e**, SPR analysis of pomalidomide binding to immobilized CRBN^midi^ (mean ± s.d.; *n* = 3 technical replicates). **f**, Same as **e**, but for *rac*-dHTC1. **g**, Heat map depicting CRBN FP signal (background-subtracted and normalized to DMSO) following treatment with the indicated compounds, in the presence of ENL (3 µM) or buffer control (mean; *n* = 3 biological replicates). Cooperativity values are calculated from the ratio of IC_50_ values with and without ENL. **h**, Structures of dHTC1 enantiomers and their time-dependent and dose-responsive loss of HiBiT–ENL signal in MV4;11 cells treated with *rac*-dHTC1. The HiBiT luminescence signal was normalized to the DMSO vehicle control (mean ± s.e.m.; *n* = 4 biological replicates). Source data

**Extended Data Fig. 2:**
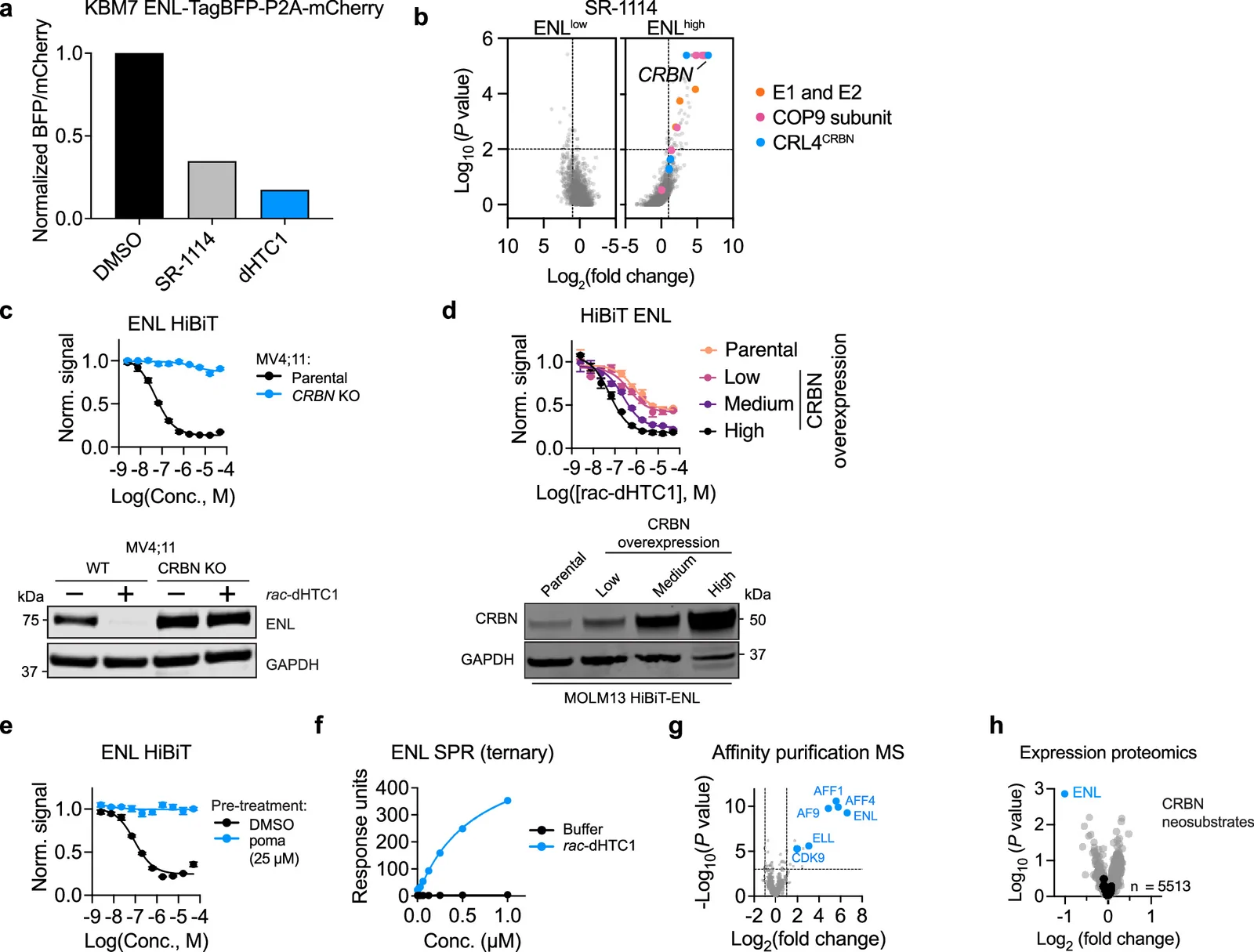
CRBN is required for dHTC1-induced ENL degradation. **a**, Validation of an ENL reporter construct. KBM7 cells expressing an ENL-TagBFP-P2A-mCherry dual fluorescence stability reporter were treated with 10 µM compound or DMSO control for 8 h (1 independent experiment). **b**, FACS-based CRISPR screens for UPS components affecting ENL stability in KBM7 reporter cells (ENL-BFP-P2A-mCherry) treated with SR-1114 (10 µM) for 8 h (6 sgRNAs targeting 1301 UPS-associated genes). Gene-level fold change and *P* values were determined by one-sided MAGeCK^114^ analysis. Same experiment as Fig. 2a. **c**, Top: HiBiT luminescence for MV4;11-HiBiT-ENL cells treated with *rac*-dHTC1 in dose response (normalized to DMSO, mean of 4 biological replicates ± S.E.M.). Bottom: immunoblot analysis of MV4;11 cells treated with *rac*-dHTC1 (10 µM, 6 h) or DMSO vehicle control. Single independent experiment. **d**, Top: immunoblot analysis of MOLM13-HiBiT-ENL cells confirming CRBN overexpression. Single independent experiment. Bottom: treatment of MOLM13-HiBiT-ENL cells with *rac*-dHTC1 in dose response. HiBiT luminescence is normalized to DMSO (mean of 3 biological replicates ± S.E.M.). Single independent experiment. **e**, MV4;11-HiBiT-ENL cells pretreated with pomalidomide (25 µM, 1 h) or DMSO control prior to *rac*-dHTC1 treatment (24 h). Luminescence normalized to DMSO vehicle control (mean of 4 biological replicates ± S.E.M.). Data in Fig. 1g are taken from the same experiment (DMSO pre-treatment is repeated in both panels). **f**, SPR analysis of CRBN^midi^ binding to immobilized ENL in the presence of a fixed concentration of *rac*-dHTC1 (1 µM) or running buffer (mean of 2 technical replicates). **g**, Scatterplot of APMS results showing relative protein abundance by label-free proteomics following enrichment of recombinant Flag-CRBN-DDB1ΔB from MOLT4 cell lysates treated with 1 µM dHTC1 or DMSO. *P* values were calculated using a moderated two-sided t-test implemented in the limma package (4 independent replicates of each treatment group). Select super elongation complex members are shown in blue. **h**, Same data presented in Fig. 1h, but with black dots indicating common CRBN neosubstrates^43^ detected by proteomics mass spectrometry. Source data

We next used a forward genetic screen to identify the effectors responsible for dHTC1 activity, which was enabled by a dual fluorescence ENL stability reporter (ENL–TagBFP–P2A–mCherry) expressed in KBM7 cells (Extended Data Fig. 2a)^33–36^. A ubiquitin proteasome system (UPS)-focused library of single guide RNAs (sgRNAs) was screened in these cells for the ability to modify the activity of *rac*-dHTC1 or SR-1114 (Fig. 2a and Extended Data Figs. 1a and 2b), a CRBN-based ENL/AF9 PROTAC that we previously disclosed^26^. Consistent with the known mechanisms of CRBN-based PROTACs^37^, SR-1114 activity was most impacted by sgRNAs targeting CRL4^CRBN^, the proteasome and the constitutive photomorphogenesis 9 signalosome (Extended Data Fig. 2b). Strikingly, the same machinery was identified for *rac*-dHTC1, with CRBN being the most substantially enriched hit (Fig. 2a).

Using population-level *CRBN*-knockout (KO) MV4;11 cells^30^, we confirmed that CRBN is required for the degradation of ENL by *rac*-dHTC1 (Extended Data Fig. 2c). We also tested whether overexpression of CRBN can improve *rac*-dHTC1 activity in MOLM-13 cells (Extended Data Fig. 2d and Supplementary Fig. 1b), where its activity was found to be relatively weak (Fig. 1e and Extended Data Fig. 1h,i). Indeed, the potency and depth of degradation were both improved in proportion to the extent of CRBN overexpression (Extended Data Fig. 2d). Furthermore, a saturating concentration of pomalidomide rescued ENL degradation (Fig. 2b and Extended Data Fig. 2e), pointing toward a mechanism whereby *rac*-dHTC1 contacts the IMiD-binding site of CRBN. This would presumably rely on its spirosuccinimide moiety, which bears resemblance to both the glutarimide motif present in thalidomide analogs and the asparagine cyclic imide modification proposed to be a natural degron recognized by CRBN^38–40^.

In vitro, we could demonstrate that the ENL YEATS domain interacts with CRBN–DDB1 (damage-specific DNA-binding protein 1) and the newly reported CRBN^midi^ construct^41^ but only in the presence of the compound (Fig. 2c and Extended Data Fig. 2f). We also used affinity purification–mass spectrometry (AP–MS) to map the interactions of CRBN globally^42^, which revealed an exquisitely selective and dHTC1-dependent enrichment of ENL, AF9 and other members of the super elongation complex (SEC) (Extended Data Fig. 2g and Supplementary Table 5). Interestingly, we found that dHTC1 did not enrich for any common CRBN neosubstrate targets (using a previously collated list^43^), nor did it degrade any (Extended Data Fig. 2g,h). This contrasts with SR-1114, which induces degradation of Ikaros-family zinc-finger protein 1 (IKZF1), as we previously reported^26^.

### dHTC1 engagement of CRBN is dependent on initial ENL binding

Several observations indicated that dHTC1 does not function like a conventional CRBN-based degrader. First, using a CRBN engagement assay based on the competitive displacement of the CRBN-based BRD4 PROTAC dBET6 (refs. ^44–46^), we failed to detect target engagement in cells (Fig. 2d). Whereas SR-1114 and the CRBN ligand pomalidomide both blocked dBET6-induced BRD4–HiBiT degradation by engaging CRBN, neither *rac*-dHTC1 nor its spirosuccinimide building block JS-1 could do so (Fig. 2d). Second, in contrast to the submicromolar affinity we observed for pomalidomide binding to CRBN^midi^ by surface plasmon resonance (SPR) (Fig. 2e), dHTC1 showed weak and nonspecific (that is, unsaturable) binding and an estimated *K*_D_ of nearly 50 µM (Fig. 2f). To further assess target engagement in vitro, we used a competitive fluorescence polarization (FP) assay that reports on the displacement of pomalidomide–BODIPY from purified preparations of recombinant CRBN–DDB1 (ref. ^14^). Unlike thalidomide, which showed submicromolar potency in dose–response assays, we did not detect inhibition by *rac*-dHTC1 until it reached a concentration of 50 µM (Fig. 2g and Supplementary Table 6). Even at this high concentration—orders of magnitude above its DC_50_—we did not observe complete inhibition of the FP (Fig. 2g). Likewise, JS-1 showed no inhibition of the CRBN FP assay, even at 50 µM (Fig. 2g).

On the basis of these data, we hypothesized that *rac*-dHTC1 might require a preformed complex with ENL to engage the IMiD-binding site with high affinity. Indeed, addition of purified recombinant ENL YEATS domain to the FP assay shifted the half-maximal inhibitory concentration (IC_50_) of *rac*-dHTC1 from 23 µM to 106 nM (Fig. 2g), resulting in a calculated 220-fold cooperativity *α*-factor^47^. By comparison, the thalidomide-based ENL PROTAC, SR-1114, also showed a shift in the presence of ENL YEATS but the cooperativity was substantially lower (*α* = 2.3) and it was fully capable of inhibiting FP signal in the absence of ENL YEATS (Fig. 2g). In fact, without ENL YEATS, the activity of SR-1114 (IC_50_ = 155 nM) closely matched its parent ligand, thalidomide (IC_50_ = 197 nM), highlighting the difference between dHTC1 and traditional CRBN-based degraders that engage CRBN independently.

### Structure–activity relationship of ENL-driven CRBN binding

**Extended Data Fig. 3:**
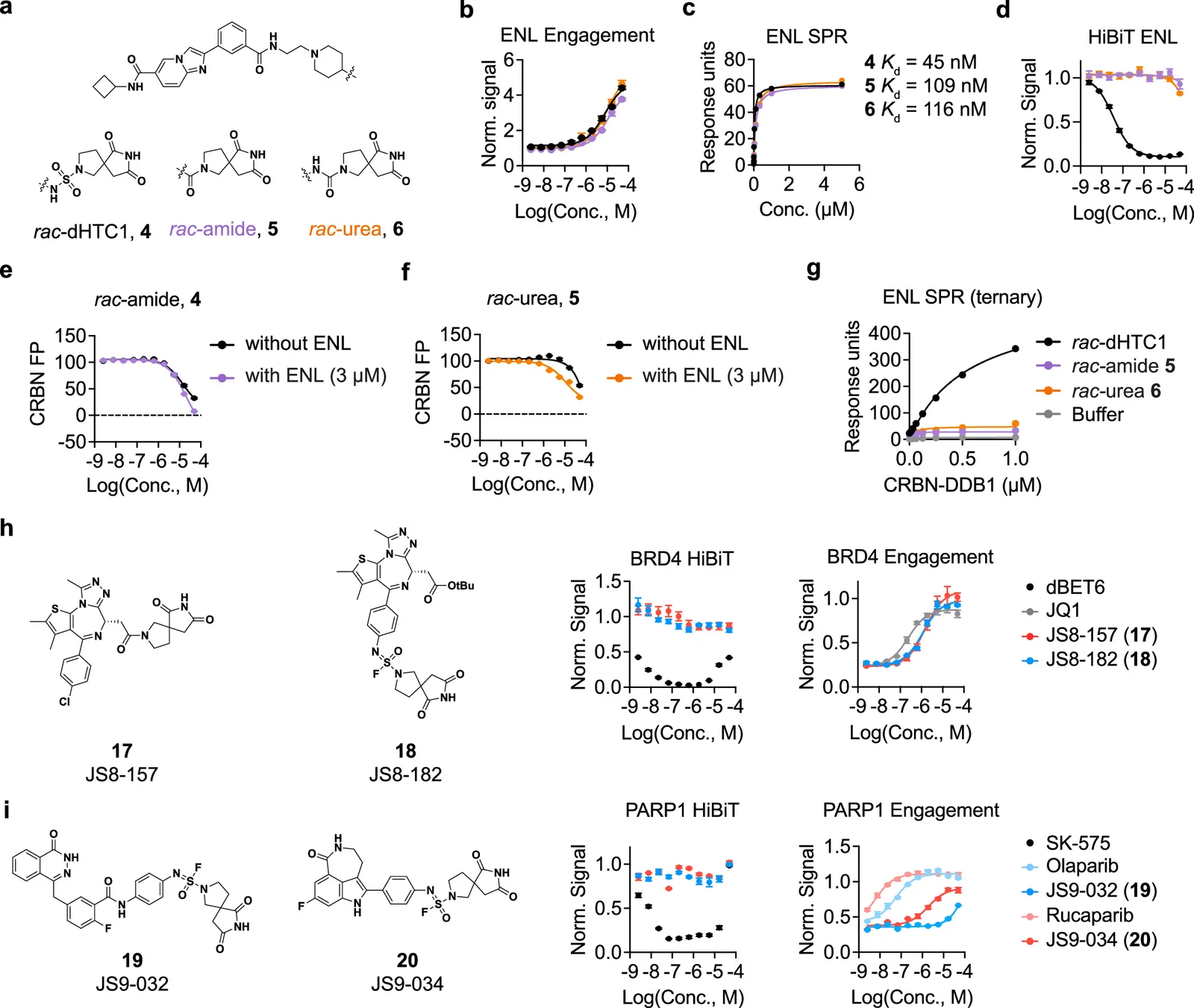
dHTC1 structure-activity relationship. **a**, Structure of dHTC1 analogs. **b**, HiBiT-based intracellular ENL YEATS target engagement assay (3 h). MLN4924 pre-treatment (1 µM, 1 h) was used to prevent the potential for compound-induced degradation. Signal is normalized to DMSO-treated cells (mean, 4 biological replicates ± S.E.M). Data in Extended Data Fig. 4d is taken from the same experiment (*rac*-dHTC1 data are repeated in both panels). **c**, Binding to immobilized ENL YEATS as measured by SPR (mean of 2 technical replicates.). Data in Extended Data Fig. 4c is taken from the same experiment (*rac*-dHTC1 data are repeated in both panels). **d**, HiBiT-based ENL degradation assay (24 h). HiBiT luminescence signal is normalized to DMSO-treated cells (mean, 4 biological replicates ± S.E.M.). **e**, CRBN FP assay for **7** in the presence of recombinant ENL YEATS domain (3 µM) or buffer (mean of 3 biological replicates ± S.E.M., background subtracted and normalized to DMSO). **f**, Urea analog **8** (same as panel **e**). **g**, SPR analysis of CRBN-DDB1 binding to immobilized ENL in the presence of a fixed concentration of compound (1 µM) or running buffer (mean of 2 technical replicates). Same experiment as Fig. 2c (*rac*-dHTC1 and buffer are repeated in both panels). **h**, Structure of two JQ1 analogs functionalized with the spirosuccinimide building block, BRD4 intracellular target engagement in MV4;11-BRD4-HiBiT cells (1 h, HiBiT luminescence normalized to DMSO, 3 biological replicates ± S.E.M.), and BRD4 degradation assay in MV4;11-BRD4-HiBiT cells (16 h, HiBiT luminescence normalized to DMSO, mean, 4 biological replicates ± S.E.M.). **i**, Structure of PARP1/2 inhibitor scaffolds functionalized with the spirosuccinimide building block, PARP1 intracellular target engagement in MV4;11-PARP1-HiBiT cells after 4 h treatment (HiBiT luminescence signal normalized to DMSO, mean of 3 biological replicates ± S.E.M.), and HiBiT degradation assay in MV4;11-PARP1-HiBiT cells after 16 h treatment (HiBiT luminescence signal normalized to DMSO, mean of 4 biological replicates ± S.E.M.). Compounds are compared to SK-575, a known PARP1 PROTAC degrader, or rucaparib and olaparib, which are known PARP1 ligands. Source data

To evaluate the mechanism of CRBN engagement further, we assessed the dHTC1 structure–activity relationship (SAR) by replacing the sulfamide connection with an amide, yielding *rac*-dHTC1-amide (**5**), or with a urea, yielding *rac*-dHTC1-urea (**6**). These analogs showed relatively similar binding to ENL in vitro and in cells but neither could provoke ENL degradation (Extended Data Fig. 3a–d). Both analogs showed weak inhibition of the CRBN FP assay—in both the presence and the absence of ENL—and neither compound could mediate ternary complex formation (Extended Data Fig. 3e–g), demonstrating that dHTC1 activity is dependent on its ability to cooperatively engage CRBN. Therefore, we would not expect that its spirosuccinimide modification would yield an active degrader when installed onto ligands for other targets. To test this, we synthesized two bromodomain and extraterminal (BET) ligands and two poly(ADP-ribose) polymerase (PARP1/2) ligands harboring JS-1 at previously established exit vector positions, none of which succeeded in degrading their targets (Extended Data Fig. 3h,i).

**Extended Data Fig. 4:**
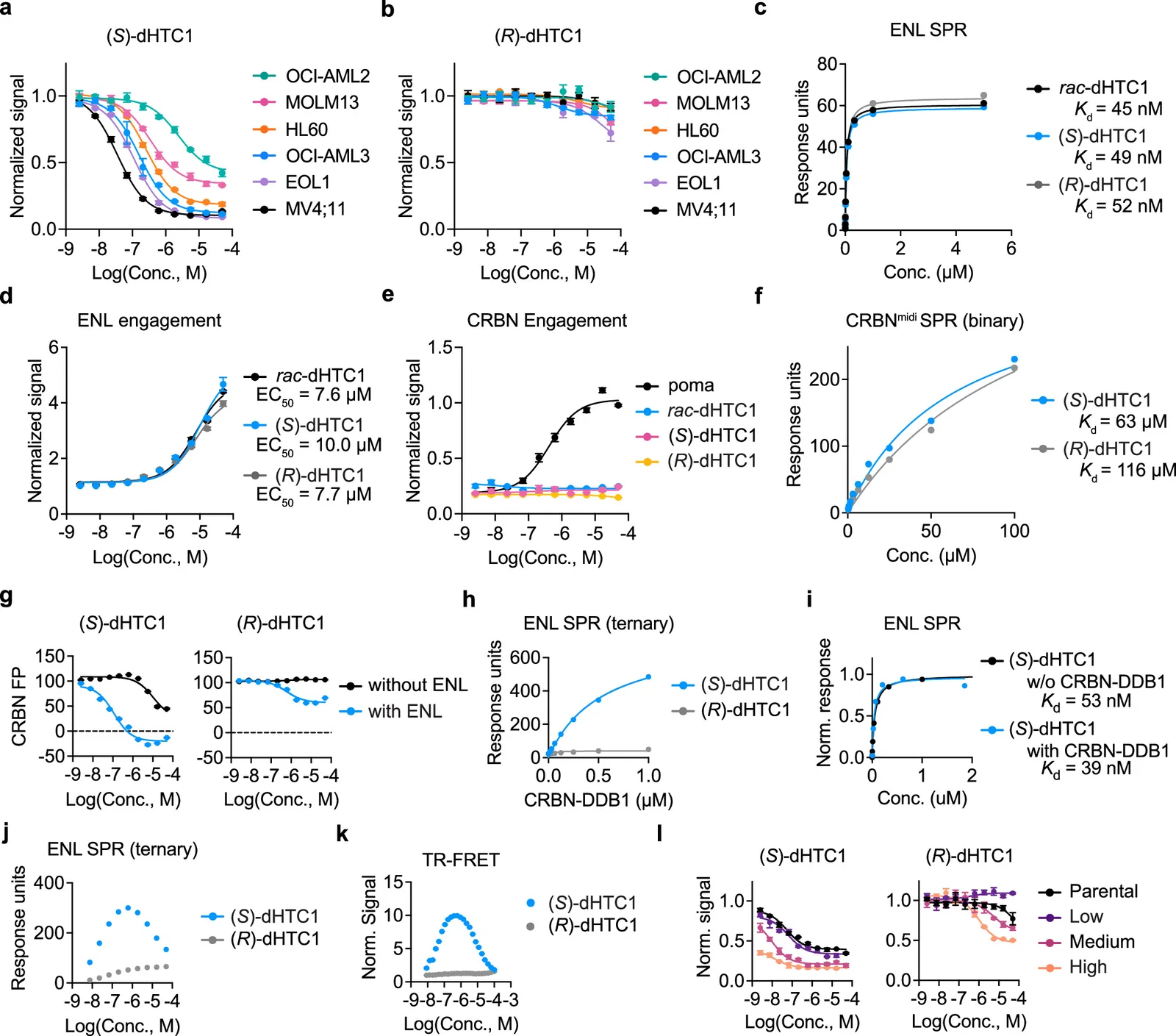
Stereoselective effects of (*S*)-dHTC1. **a**, HiBiT degradation assay in MV4;11-HiBiT-ENL cells (signal normalized to DMSO treated cells, 4 biological replicates). **b**, (*R*)-dHTC1, same as panel **a**. **c**, SPR analysis of binding to immobilized ENL (mean of 2 technical replicates). Data in Extended Data Fig. 3c is taken from the same experiment (*rac*-dHTC1 data is repeated in both panels). **d**, HiBiT-based intracellular ENL YEATS target engagement assay (3 h). OCI-AML2 cells were pretreated with MLN4924 (1 µM, 1 h) to prevent compound-induced degradation. Signal is normalized to DMSO-treated cells (4 biological replicates). Data in Extended Data Fig. 3b is taken from the same experiment (*rac*-dHTC1 data is repeated in both panels). **e**, Intracellular CRBN target engagement assessed in MV4;11-BRD4-HiBiT via rescue of dBET6-induced BRD4 degradation. Cells pretreated with compounds (2 h) in dose response before dBET6 (500 nM, 1 h, 3 biological replicates). Data for pomalidomide is repeated in Fig. 2d, which originated from a single experiment. **f**, SPR analysis of binding to immobilized CRBN^midi^ (3 technical replicates). **g**, CRBN FP signal following treatment with the indicated compound in the presence of ENL YEATS (3 µM) or buffer control (FP signal is background subtracted and normalized to DMSO, 3 biological replicates). **h**, SPR analysis of CRBN–DDB1 binding to immobilized ENL YEATS in the presence of 1 µM (*S*)- or (*R*)-dHTC1 (2 technical replicates). **i**, (*S*)-dHTC1 binding to ENL measured by SPR, with or without CRBN-DDB1 (500 nM) present (2 technical replicates). Each trace is normalized to response at saturation. (*S*)-dHTC1 without CRBN-DDB1 data is from panel **c**. (S)-dHTC1 with CRBN-DDB1 is from panel **j**. **j**, SPR analysis of CRBN-DDB1 (500 nM) binding to immobilized ENL YEATS in the presence of an (*S*)- or (*R*)-dHTC1 dose response (2 technical replicates). **k**, HTRF-based assay to measure induced proximity of ENL YEATS and CRBN-DDB1 with (*S*)- and (*R*)-dHTC1 (HTRF signal normalized to DMSO, 3 technical replicates). **l**, HiBiT degradation assay in MOLM13-HiBiT-ENL cells overexpressing CRBN (HiBiT luminescence signal normalized to DMSO, 3 biological replicates). **a**,**b**,**d**-**g**,**k**,**l**, mean ± S.E.M. **c**,**h**-**j**, mean. Source data

To further probe dHTC1 SAR, we separated its two enantiomers by preparative supercritical fluid chromatography (SFC) and assigned absolute stereochemical configurations using vibrational circular dichroism (VCD) (Fig. 2h and Supplementary Methods). Whereas (*S*)-dHTC1 (**7**) was able to elicit potent ENL degradation, (*R*)-dHTC1 (**8**) was completely inactive (Fig. 2h and Extended Data Fig. 4a,b). Both enantiomers bound to ENL identically in vitro (*K*_D_ = 49–52 nM) and in cells (half-maximal effective concentration = 8 µM) and both showed the same weak CRBN engagement as the racemate (Extended Data Fig. 4c–f). However, (*S*)-dHTC1 showed potent and cooperative engagement of CRBN in the presence of ENL (Extended Data Fig. 4g), resulting in the stereoselective formation of a ternary complex (Extended Data Fig. 4h,i). Therefore, although CRBN shows low affinity for dHTC1 and no detectable binding to ENL up to a concentration of 1 µM, the stable, preformed ENL:(*S*)-dHTC1 complex can cooperatively engage CRBN with high apparent affinity to induce stereochemistry-dependent ENL degradation.

Interestingly, we observed a strong hook effect for ternary complex formation in vitro using SPR and time-resolved fluorescence resonance energy transfer (TR-FRET) assays (Extended Data Fig. 4j,k). This might relate to the lack of cooperativity seen for ENL engagement by dHTC1 (Extended Data Fig. 4i), which contrasts with its highly cooperative engagement of CRBN. However, the observation of a hook effect is context dependent and influenced by the relative concentrations of all three members of a ternary complex^48^, which may differ between native cellular environments and reconstituted biochemical assays. Therefore, it is unclear how the strong hook effect we observed in vitro is relevant to the cooperative formation of a ternary complex in cells. (*S*)-dHTC1 produces only a weak hook effect in degradation assays (Fig. 2h and Extended Data Fig. 4a), suggesting that the formation of a ternary complex is favored over the binary complexes under physiologic conditions. Indeed, in the presence of CRBN overexpression, (*S*)-dHTC1 induces maximal degradation at concentrations spanning more than three orders of magnitude with no apparent hook effect at any time point (Extended Data Fig. 4l). While the racemate produces a stronger hook effect in degradation assays, this occurs only at early time points and high concentrations (Fig. 1f), which could possibly be attributed to competition from the equimolar presence of (*R*)-dHTC1, which binds to ENL with equal affinity. Given the weak independent CRBN engagement by dHTC1 (Extended Data Fig. 4e), we interpret the totality of data to suggest that degradation by dHTC1 is dependent on the cooperative engagement of CRBN.

### Genetic determinants of CRBN-dependent dHTC1 activity

**Extended Data Fig. 5:**
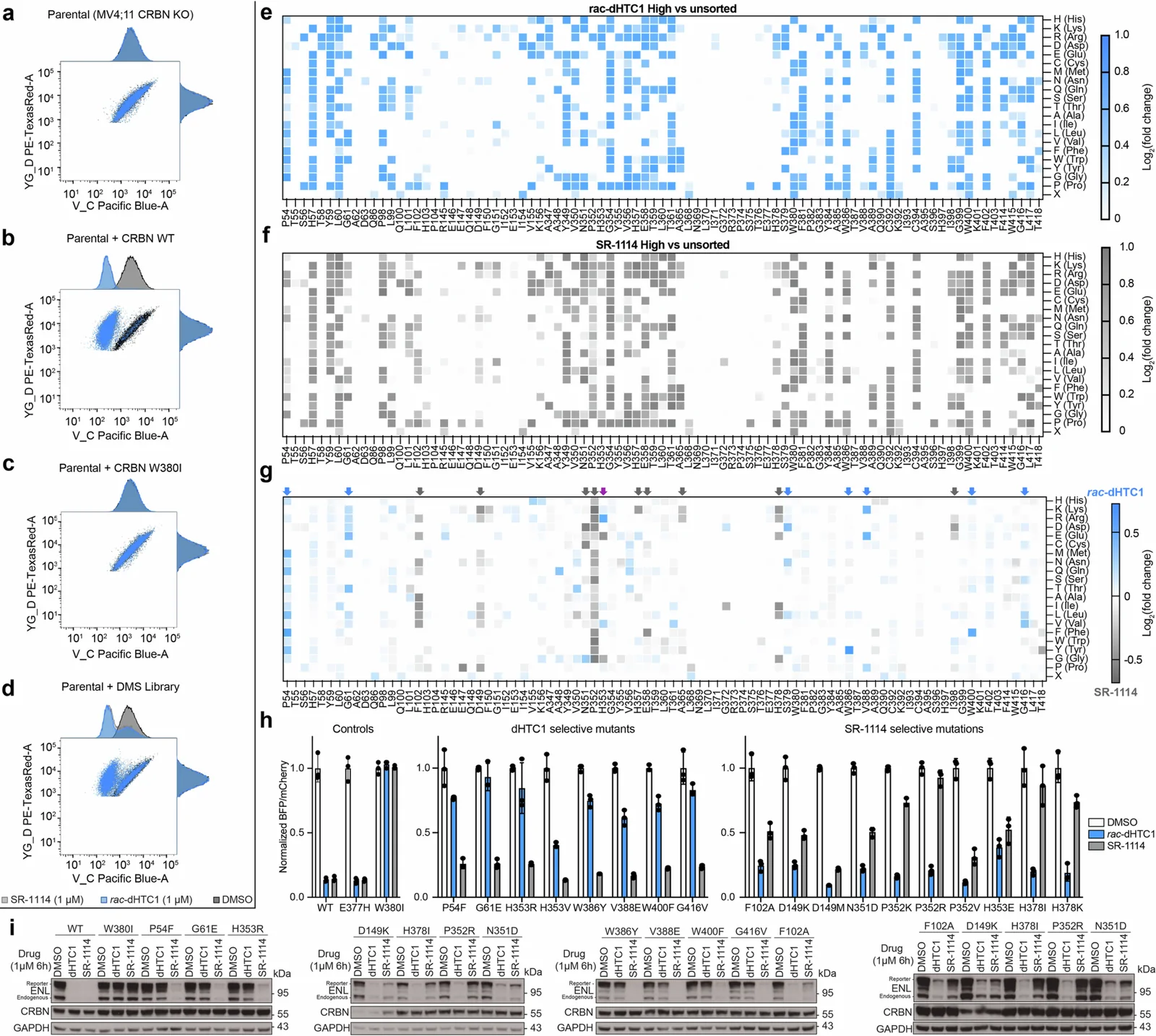
CRBN DMS screen and validation of results. **a**, Flow cytometry following treatment in MV4;11 CRBN KO “parental” cells expressing ENL-TagBFP-P2A-mCherry. Histograms for SR-1114, *rac*-dHTC1 and DMSO treatments overlap as ENL degradation is CRBN-dependent. **b**, Reintroduction of wild-type CRBN restores ENL degradation. **c**, Reintroduction of CRBN with inactivating mutation (W380I) fails to mediate ENL degradation. **d**, Cells transduced with CRBN DMS library regained activity; roughly 70% of cells showing ENL degradation. **e**, MV4;11 CRBN KO cells expressing ENL-TagBFP-P2A-mCherry were transduced with a DMS library, which reintroduced CRBN with single amino acid mutations within a 10 Å radius of the thalidomide binding site. After these cells were treated with *rac*-dHTC1 (1 µM, 8 h), endpoint ENL levels were quantified by flow cytometry. Results are depicted indicating log_2_ fold change of ENL^high^ over the unsorted pools. **f**, SR-1114 (same as **e**). **g**, Heatmap comparing CRBN DMS results for *rac*-dHTC1 and SR-1114. Arrows above indicate residues showing selectivity for dHTC1 (blue), SR-1114 (gray), or mixed (purple) that were selected for follow-up assays. **h**, Validation of altered sensitivity to ENL degraders by individual CRBN mutants. MV4;11 *CRBN* knockout cells expressing ENL-TagBFP-P2A-mCherry reporter with 1 µM of *rac*-dHTC1 or 1 µM of SR-1114 (8 h), assessed by flow cytometry (BFP/mCherry normalized to DMSO, 3 technical replicates, 2 independent experiments ± S.D.). **i**, Immunoblot analyses of ENL levels in MV4;11 *CRBN* knockout cells expressing ENL-TagBFP-P2A-mCherry. Wild-type or mutant CRBN was reintroduced by lentiviral expression to evaluate mutations that selectively impact the ability of dHTC1 or SR-1114 to degrade ENL (1 µM, 6 h). For ENL, the lower molecular weight band is endogenous protein and higher molecular weight band is reporter construct. Representative of 2 independent experiments. Source data

The ENL-dependent binding of dHTC1 to CRBN invokes a distinct mechanism of action relative to prior CRBN-based degraders. To further characterize this mechanism, we coupled deep mutational scanning (DMS) of *CRBN* to a fluorescence-activated cell sorting (FACS)-based assessment of ENL degradation in the presence of *rac*-dHTC1 or the ENL PROTAC, SR-1114 (Extended Data Fig. 5a–d). Using our previously described DMS library that covers all possible point mutations within 10 Å of the IMiD-binding site of CRBN^49^, we found that *rac*-dHTC1 and SR-1114 are sensitive to similar *CRBN* mutations (Extended Data Fig. 5e,f, and Supplementary Tables 7 and 8). However, several mutations were suggested by the screen to preferentially confer resistance to either *rac*-dHTC1 or SR-1114, which we validated by immunoblot and flow cytometry (Extended Data Fig. 5g–i).

**Extended Data Fig. 6:**
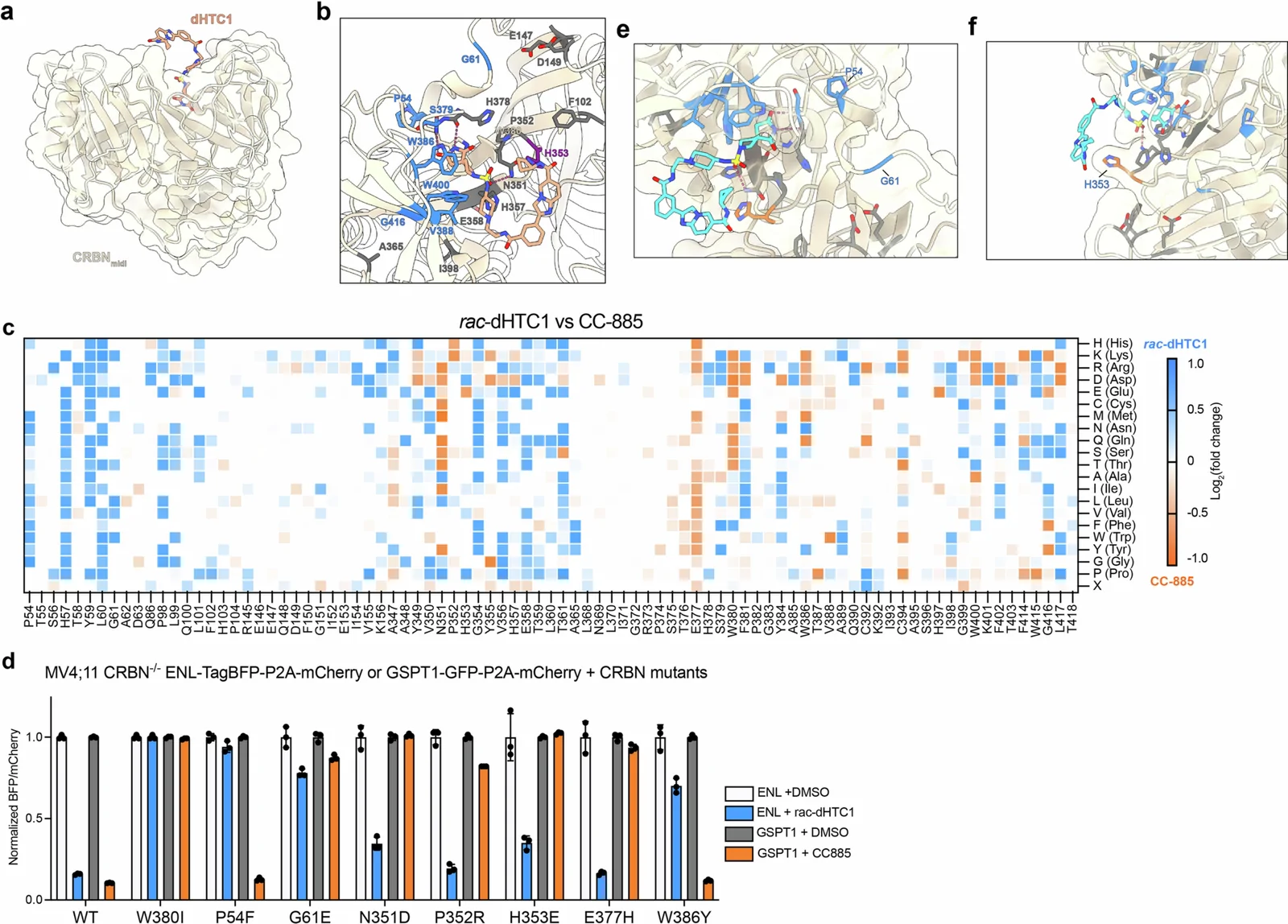
Structural features of dHTC1 bound to CRBN. **a**, Co-crystal structure of (*S*)-dHTC1 bound to CRBN^midi^. **b**, View of the IMiD-binding site occupied by (*S*)-dHTC1. Residues indicated with an arrow in Extended Data Fig. 5g are colored accordingly. **c**, Differential activity between CRBN DMS screen for RKO cells treated with CC-885 (500 nM, 7 d)^49^ and MV4;11-ENL-TagBFP-P2A-mCherry cells *rac*-dHTC1 (1 µM, 8 h). **d**, Validation of DMS screening results, comparing CRBN mutations in MV4;11 CRBN^−/−^ GSPT1-GFP-P2A-mCherry cells treated with CC-885 versus MV4;11 CRBN^−/−^ ENL-TagBFP-P2A-mCherry cells treated with *rac*-dHTC1. BFP/mCherry signal is normalized to respective DMSO control (3 technical replicates, 2 independent experiments ± S.D.). **e**, View of P54 and G61 residues distal ( > 10 Å) to the IMiD-binding pocket. **f**, View of CRBN H353. Source data

To investigate how these mutations might impact dHTC1 activity selectively, we determined the cocrystal structure of (*S*)-dHTC1 bound to CRBN at a resolution of 2.2 Å using CRBN^midi^ (Extended Data Fig. 6a and Supplementary Table 9)^41^. This showed (*S*)-dHTC1 occupying the IMiD-binding site of CRBN, positioning its spirosuccinimide moiety within the tritryptophan cage formed by W380, W386 and W400 (Extended Data Fig. 6b). The succinimide of dHTC1 makes hydrogen-bonding interactions with the side chain of H378, backbone carbonyl of H378 and backbone nitrogen of W380, which are all similar to the contacts made by the glutarimide of lenalidomide^50^. However, apart from the sulfamide making hydrogen-bonding contacts with the side chains of N351 and W400, the remainder of dHTC1, which is responsible for binding ENL, makes minimal contacts with CRBN.

Focusing on mutations that selectively affected the activity of dHTC1 or SR-1114, we found that these residues generally mapped to distinct hemispheres of the IMiD-binding site (Extended Data Fig. 6b). As several of these residues are buried within the IMiD-binding site, we hypothesized that they might reflect differences in the binding mode of the dHTC1 spirosuccinimide compared to traditional glutarimide-based CRBN ligands. To evaluate this, we compared our DMS results to a previously published screen of CC-885, a thalidomide analog that degrades G1 to S phase transition 1 (GSPT1) (Extended Data Fig. 6c)^13,49^. This comparison indicated that a W386Y substitution, which impacts the activity of dHTC1 but not SR-1114, also does not impact CC-885 activity (Extended Data Fig. 6c,d). Likewise, several P352 substitutions that affected the activity of SR-1114 but not dHTC1 also affected CC-885 (Extended Data Fig. 6c,d).

We also identified compound-selective mutations mapping to regions outside of the direct IMiD-binding site. For example, P54 and G61 substitutions, which affect dHTC1 more broadly than SR-1114 (Extended Data Fig. 5e,f), are located far from the IMiD-binding site on another face of CRBN (Extended Data Fig. 6e). Another, H353, which is adjacent to the IMiD-binding site but presents outward into the solvent (Extended Data Fig. 6f), showed varying behavior, with some substitutions impairing dHTC1 but not SR-1114 or CC-885 and others affecting SR-1114 and CC-885 but not dHTC1 (Extended Data Figs. 5e–i and 6c,d). These data pointed to the possibility that the cooperative engagement of CRBN might depend on contacts made outside of the 10-Å radius included in the DMS screen.

### Extensive contacts stabilize dHTC1-bound ENL on CRBN

**Fig. 3:**
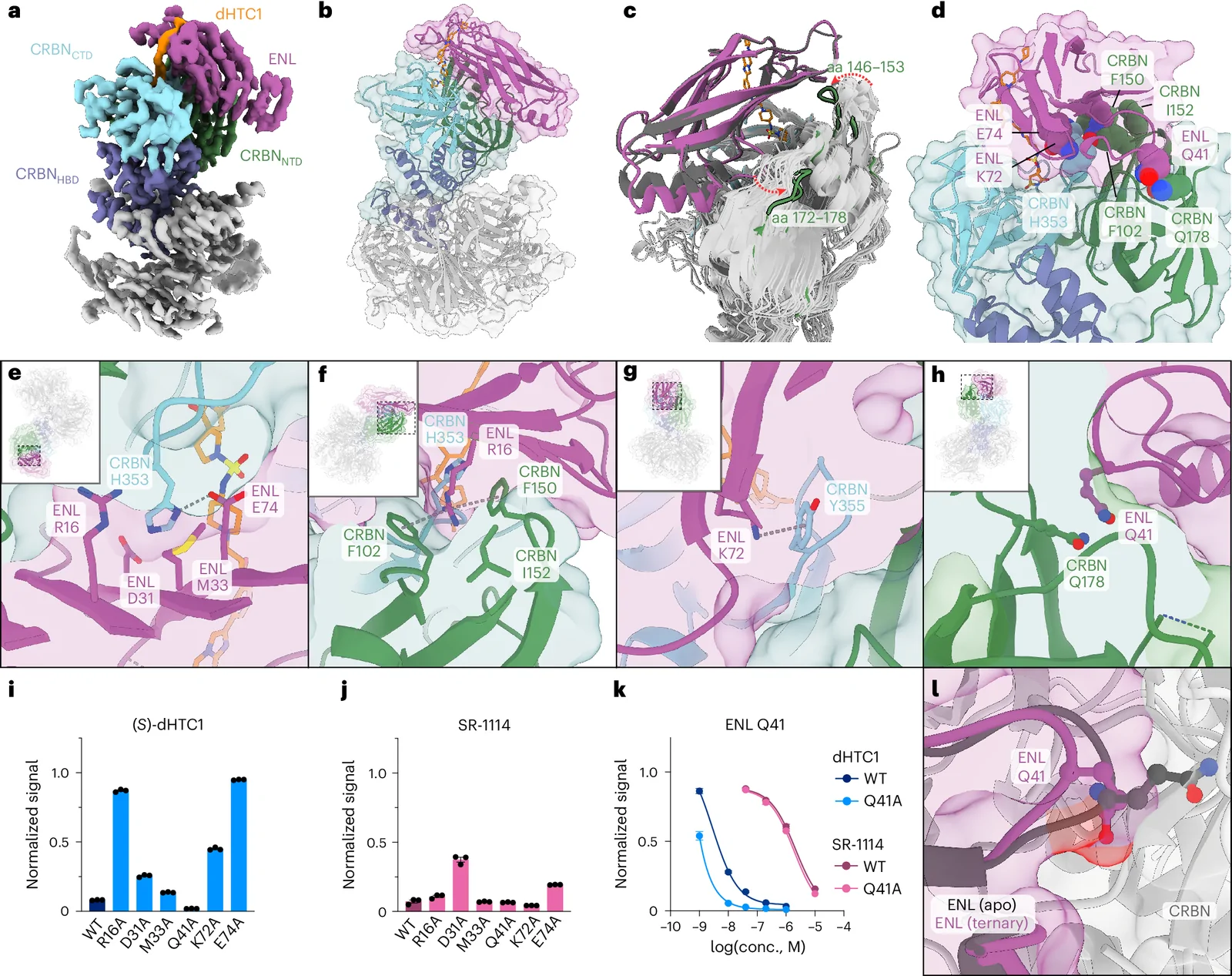
(*S*)-dHTC1 binding is stabilized by extensive protein–protein contacts between ENL and CRBN. **a**, EM map of DDB1:CRBN bound to (*S*)-dHTC1 and ENL colored by protein and domain. CTD, C-terminal domain; NTD, N-terminal domain; HBD, helical bundle domain. **b**, Model of DDB1:CRBN bound to (*S*)-dHTC1 and ENL complex. **c**, Superposition of reported ligand-bound CRBN structures (gray) reveals a movement of CRBN loops (red arrows). **d**, View of CRBN–ENL interface with select contacting residues shown with space-filling representations. **e**–**h**, Views of CRBN and ENL residues making intermolecular protein–protein contacts, focused on CRBN H353 (**e**), ENL R16 (**f**), ENL K72 and CRBN Y355 (**g**), and ENL Q41 and CRBN Q178 (**h**). Dashed lines indicate interactions. **i**, Degradation of WT or mutant ENL–BFP reporter in MV4;11 cells. ENL–TagBFP and internal mCherry control were measured by flow cytometry following dHTC1 (1 µM) treatment for 24 h (BFP or mCherry, normalized to DMSO-treated cells; mean ± s.e.m.; *n* = 3 biological replicates). **j**, Same as **i**, but for treatment with SR-1114 (10 µM). **k**, ENL–TagBFP (WT or Q41A) degradation in MV4;11 cells (BFP or mCherry, normalized to DMSO control; mean ± s.e.m.; *n* = 3 biological replicates). **l**, Superposition of the apo ENL YEATS domain crystal structure (PDB 6HQ0 (ref. ^115^), dark gray) with the dHTC1-bound ENL YEATS–CRBN–DDB1 structure showing the Q41 side chain. Source data

**Extended Data Fig. 7:**
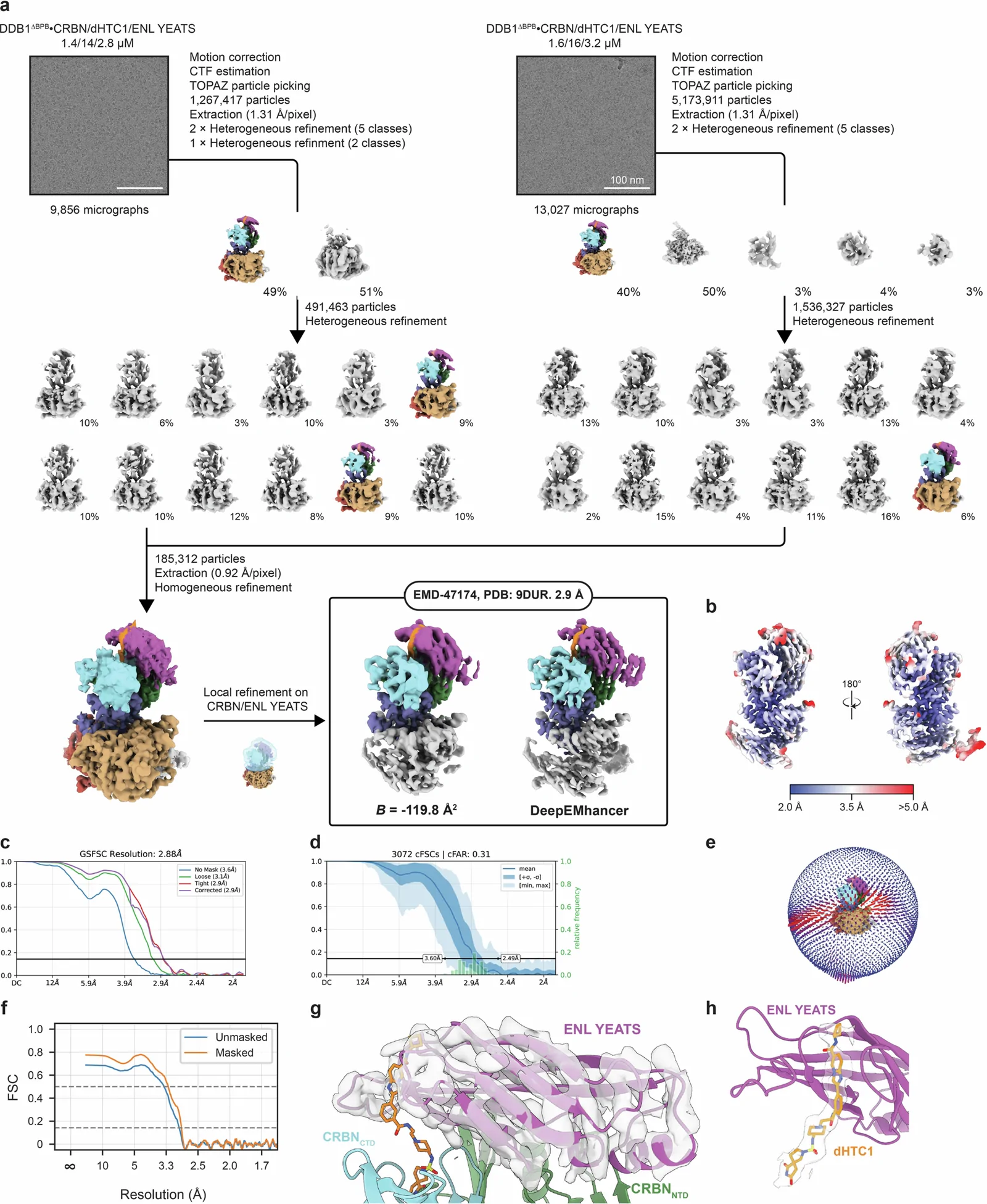
Cryo-EM processing workflow for the CRBN•ENL YEATS structure. **a**, Overview of processing workflow from raw micrograph (scale bar indicated) to final maps. Particles belonging to colored volumes were taken into subsequent steps. Contour levels of maps after homogeneous refinement, local refinement, and postprocessing with DeepEMhancer are 0.025, 0.03, and 0.10 respectively. **b**, Local resolution mapped onto final map postprocessed by DeepEMhancer, **c**, FSC plot, **d**, Directional FSC plot. **e**, Viewing distribution. **f**, Model-to-map FSC for 9DUR in EMD-47174. **g, h**, Cryo-EM density examples (threshold 0.10) for ENL YEATS and dHTC1 (map postprocessed by DeepEMhancer).

**Extended Data Fig. 8:**
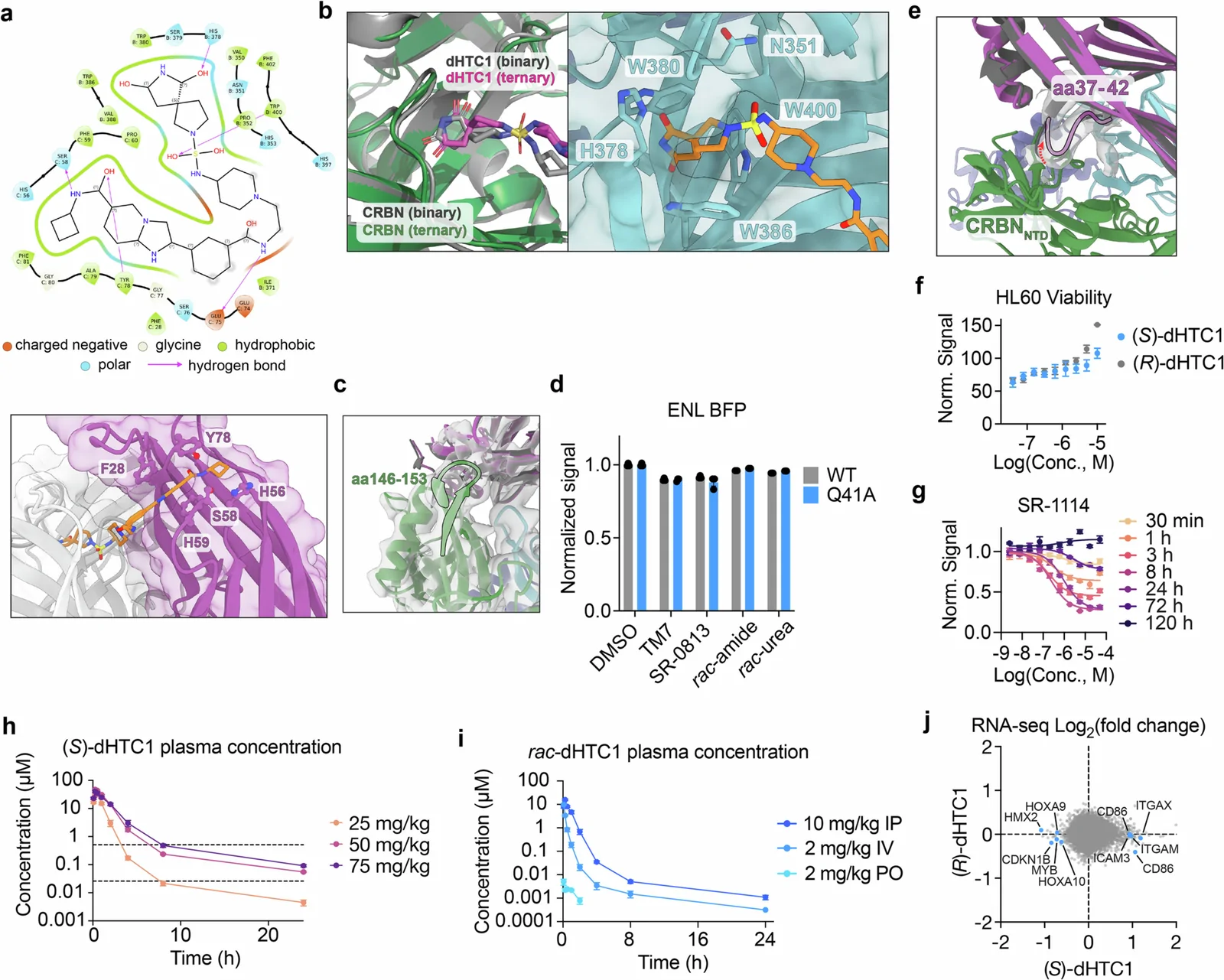
Characterization of dHTC1 and SR-1114 activity. **a**, Top, a two-dimensional interaction map of dHTC1 bound to CRBN and ENL and, bottom, dHTC1 bound to ENL YEATS with key interactions highlighted. **b**, Left, an overlay of dHTC1 binding pose with CRBN in binary and ternary complex structures and, right, binding pose of dHTC1 with CRBN in the ternary complex with key amino acid residues highlighted. **c**, Peripheral CRBN loop (aa146-153) and its density (silver, threshold). **d**, MV4;11 ENL-TagBFP-P2A-mCherry cells (wild type or Q41A) treated with 1 µM of the indicated compounds or DMSO. BFP percentage normalized to DMSO (mean, 3 biological replicates ± S.E.M.)**. e**, Superposition of the apo ENL YEATS domain crystal structure (6HQ0^115^, dark gray) to the dHTC1-bound ENL YEATS-CRBN/DDB1 structure showing ENL density (silver, threshold 0.03). Red arrow indicates movement of the ENL loop relative to the apo structure (aa 37-42). **f**, Viability of HL60 cells after 12-day treatment (cells were split 1:10 and fresh drug was added every 3 days) with (*S*)-dHTC1 or (*R*)-dHTC1 measured by ATP-dependent luminescence (normalized to DMSO, 3 independent experiments, mean of 3 biological replicates ± S.E.M.). **g**, Time-dependent and dose-responsive loss of HiBiT-ENL signal in MV4;11 cells treated with SR-1114. Luminescence normalized to DMSO vehicle control (mean, 4 biological replicates ± S.E.M.). **h**, Mean ± S.E.M. plasma concentration and pharmacokinetic properties of male C57BL/6 mice dosed with (*S*)-dHTC1 (25, 50 or 75 mg/kg) via intraperitoneal injection (*n* = 3 mice). 50 mg/kg data is plotted in Fig. 4b. **i**, Mean ± S.E.M of plasma concentration of male C57BL/6 mice dosed with *rac*-dHTC1 at 10 mg/kg via intraperitoneal (IP) injection, 2 mg/kg via intravenous (IV) injection or 2 mg/kg *per os* (PO) (*n* = 9 mice). **j**, Scatter plot of RNA-seq analysis of mouse-cell-depleted bone marrow lysates from MV4;11 xenograft (same experiment as data from Fig. 4e). Source data

To understand how dHTC1 recruits CRBN when bound to ENL, we determined a ternary complex structure of DDB1^∆BPB^•CRBN•(*S*)-dHTC1•ENL YEATS by cryo-electron microscopy (cryo-EM) refined to a global resolution of 2.6 Å (Supplementary Table 10). To obtain insight into the CRBN–ENL interface, we conducted local refinements focusing on CRBN•(*S*)-dHTC1•ENL YEATS leading to a resolution of 2.9 Å (Fig. 3a,b and Extended Data Fig. 7). The amido-imidazopyridine core of dHTC1 inserts into the acetyllysine-binding channel of ENL and makes canonical contacts with F28, H56, H59, S58 and Y78 (Extended Data Fig. 8a)^51^. The spirosuccinimide of (*S*)-dHTC1 engages the IMiD-binding site of CRBN but, interestingly, it adopts a slightly different pose in the ternary complex compared to what was revealed by the cocrystal structure of the binary complex (Extended Data Fig. 8b). This suggests that contacts outside of the ligand-binding site contribute to its improved affinity in the presence of ENL and, indeed, our model revealed an extensive interfacial interaction between CRBN and ENL YEATS, with a buried solvent-accessible surface area of ~2,258 Å^2^ and a high degree of shape complementarity (0.64 out of 1)^52^. The subtle change in binding pose of the spirosuccinimide warhead—sacrificing a hydrogen bond in the tritryptophan cage and reorientation of the sulfamide (Extended Data Fig. 8a,b)—is likely necessary to accommodate ENL engagement and, in turn, necessitates a rather weak primary affinity for CRBN.

Interestingly, ENL binds to CRBN primarily along its N-terminal domain, differing from previously reported ternary complex structures of CRBN-based molecular glues, which mostly rely on interactions with the C-terminal domain of CRBN^14,15,53^. On the basis of this finding, we queried for conformational rearrangements of CRBN that might stabilize its interaction with ENL by superimposing every reported structure of CRBN bound to a molecular glue in the closed conformation (Fig. 3c). This revealed that a peripheral loop in the N-terminal domain of CRBN (amino acids 146–153) is brought closer to the ENL YEATS domain than in any other structure (median root-mean-square deviation (r.m.s.d.) = 3.8 Å) and contributes to the high degree of shape complementarity between CRBN and ENL (Fig. 3c and Extended Data Fig. 8c).

Protein–protein contacts are widely distributed across the CRBN–ENL binding interface (Fig. 3d–h). CRBN H353, which is solvent exposed in the binary complex structure (Extended Data Fig. 6f), contacts several ENL residues in the ternary complex structure, most notably making a hydrogen bond with ENL E74 where it shows connected density in the EM map (Fig. 3e). Substituting ENL E74 to alanine fully blocked ENL degradation by (*S*)-dHTC1 while only modestly impacting SR-1114 activity (Fig. 3i,j). The close ENL contacts made by CRBN H353 might explain why the more extended H353R allele was found to disrupt dHTC1 activity in the DMS screen but not SR-1114 (Extended Data Fig. 5h,i). ENL R16 is surrounded by several CRBN residues in the N-terminal domain (F102, F150 and I152) and stacks between CRBN F102 and F150, whose relative positions suggest formation of a cation–*π* interaction (Fig. 3f). Notably, F150 is located within the loop on CRBN (amino acids 146–153) that shows a substantial conformational rearrangement in the ternary complex with ENL (Fig. 3c). Consistent with this interaction being essential for CRBN–ENL contacts, we found ENL-R16A to be completely resistant to (*S*)-dHTC1-mediated degradation but sensitive to SR-1114 (Fig. 3i,j). We also observed a cation–*π* interaction between CRBN Y355 and ENL K72 (Fig. 3g), with the ENL-K72A mutant showing less sensitivity to (*S*)-dHTC1 but unchanged sensitivity to SR-1114 (Fig. 3i,j). Altogether, these results demonstrate that ENL–CRBN contacts outside of their ligand-binding sites are critical for dHTC1 activity, consistent with a cooperativity-dependent mechanism of CRBN engagement by dHTC1.

We also observed a polar–polar interaction between CRBN Q178 and ENL Q41 (Fig. 3h). Surprisingly, we found that ENL-Q41A is more sensitive to (*S*)-dHTC1 but equivalently sensitive to SR-1114 and unaffected by other YEATS domain ligands or inactive analogs of dHTC1 (Fig. 3i–k and Extended Data Fig. 8d). These data suggest that the Q41A substitution may assist ENL in making more favorable contacts with CRBN in the presence of (*S*)-dHTC1. Interestingly, when we compared our cryo-EM model to the isolated crystal structure of ENL (PDB 6HQ0), we observed an inward movement of the loop containing ENL Q41 (amino acids 37–42; backbone r.m.s.d. = 1.9), which is wedged into a groove formed by the CRBN N-lobe (Extended Data Fig. 8e). Compared to other observed CRBN structures, this groove is enlarged by the β-barrel loop of CRBN (amino acids 172–178) moving outward (Fig. 3c), allowing for the formation of a polar–polar interaction between CRBN Q178 and ENL Q41 (Fig. 3h). These movements likely avoid clashes between CRBN and ENL Q41 (Fig. 3l), rationalizing our observation that ENL-Q41A is more readily degraded by dHTC1.

### HTC affords a glue with favorable biological properties

**Fig. 4:**
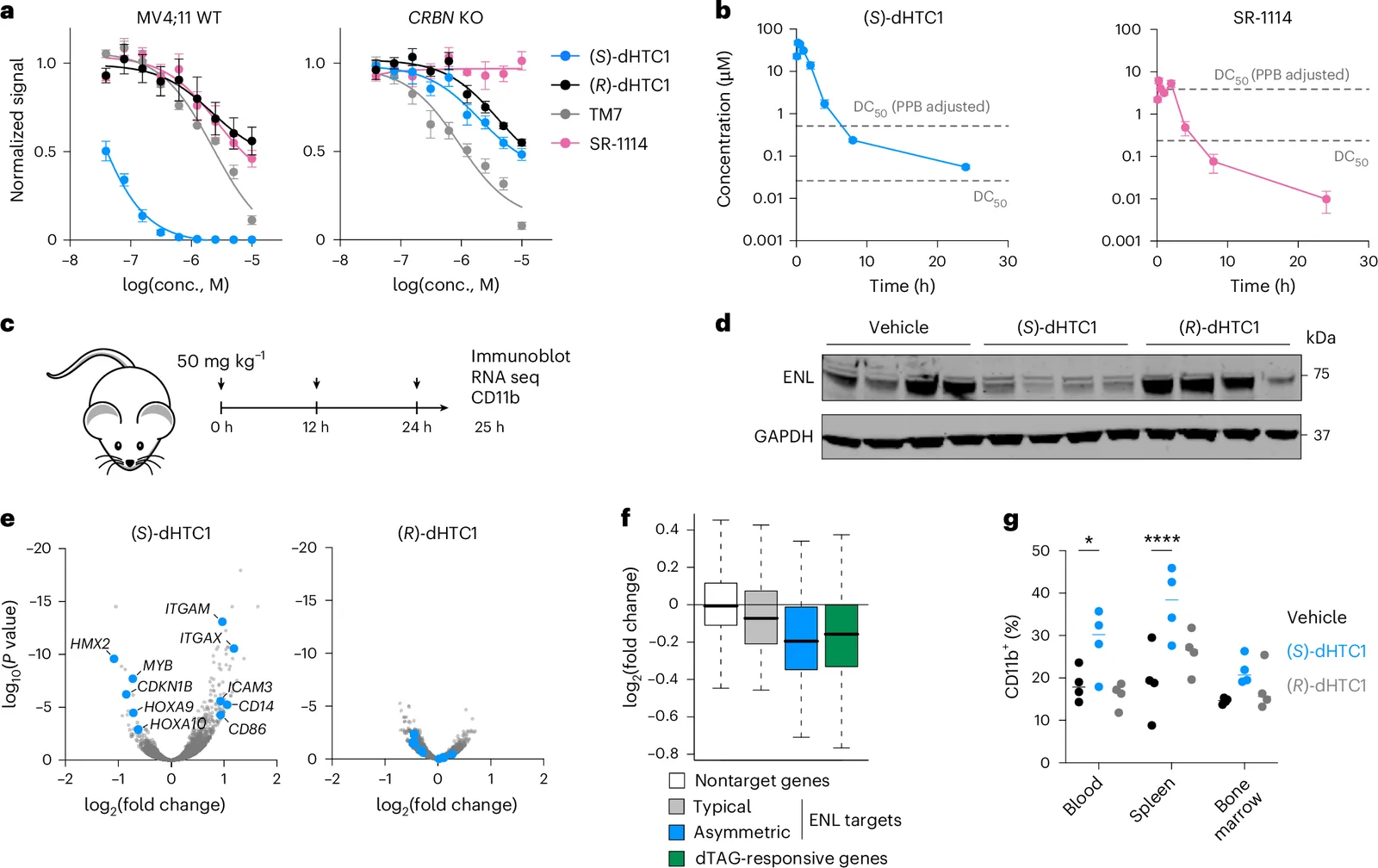
HTC affords a stereoselective degrader with enhanced in vivo properties. **a**, Viability of MV4;11 cells after 12-day treatments with (*S*)-dHTC1 or (*R*)-dHTC1. Cells were split 1:10 and fresh drug was added every 3 days; then, viability was measured by ATP-dependent luminescence (normalized to DMSO; *n* = 3 independent experiments; mean ± s.e.m.; *n* = 3 biological replicates). **b**, Mean plasma concentration of (*S*)-dHTC1 and SR-1114 in male C57BL/6 mice dosed with 50 mg kg^−1^ through intraperitoneal injection (*n* = 3 mice). Cellular DC_50_ (MV4;11 at 8 h) is depicted with and without adjustment for PPB in both plots. (*S*)-dHTC1 data are repeated in Extended Data Fig. 8h. **c**, Diagram representing the timeline of dosing and data collection for in vivo studies. **d**, Immunoblot analysis of mouse-cell-depleted bone marrow from MV4;11 xenotransplantation following treatment with vehicle, (*S*)-dHTC1 (50 mg kg^−1^) or (*R*)-dHTC1 (50 mg kg^−1^) through intraperitoneal injection (*n* = 4 mice per group). **e**, RNA-seq analysis of mouse-cell-depleted bone marrow lysates from MV4;11 xenograft (*n* = 3 individual mice per treatment condition from xenograft experiment shown in **c**). *P* values were calculated using DESeq2 (two-sided Wald’s test). Data are reproduced in Extended Data Fig. 8j. **f**, Box-and-whisker plot depicting the effect of (*S*)-dHTC1 on the mRNA expression of ENL target genes compared to vehicle control. ENL target genes, defined by typical (gray; *n* = 176 genes) and asymmetric (blue; *n* = 56 genes) ENL ChIP-seq signal strength within the gene promoter region (typical and asymmetric target genes) or by responsiveness to ENL degradation through dTAG-enabled chemically induced degradation (green; *n* = 150 genes). Nontargets include all other expressed transcripts (*n* = 14,854 genes). Center lines indicate the median, boxes indicate the first to third quartiles and whiskers represent 1.5× the interquartile range. **g**, Flow-cytometry-based quantification of hCD11b expression in indicated tissues (*n* = 3 mice; same mice as in **d**). Statistical analysis was conducted using an ordinary two-way analysis of variance with uncorrected Fisher’s least significant difference multiple-comparisons test (two-sided). *P < 0.05 and ****P < 0.0001 (for blood, *P* = 0.0182; for spleen, *P* < 0.0001.) Source data

Altogether, these data demonstrate that HTC afforded the discovery of a highly cooperative molecular glue degrader of ENL. Improved physiochemical and pharmacological properties are often cited as a key motivation for the discovery of molecular glues over heterobifunctionals. Therefore, we evaluated dHTC1 alongside SR-1114 in translationally relevant models of AML that require ENL for survival^26,54,55^. In cell culture, we found that (*S*)-dHTC1 potently and stereoselectively suppressed the growth of ENL-dependent MV4;11 cells but did not impact ENL-insensitive HL-60 cells (Fig. 4a and Extended Data Fig. 8f)^54,55^. Importantly, (*R*)-dHTC1 did not impact either cell line and KO of *CRBN* conferred resistance to (*S*)-dHTC1 in MV4;11 cells, demonstrating on-target antiproliferative activity (Fig. 4a). (*S*)-dHTC1 showed greater antiproliferative effects than ENL/AF9 YEATS domain inhibitors in wild-type (WT) MV4;11 cells but not in CRBN-deficient cells, demonstrating that ENL degradation is more effective than inhibition (Fig. 4a). However, as SR-1114 could not sustain ENL degradation beyond 24 h (Extended Data Fig. 8g), it showed weak antiproliferative activity (Fig. 4a). As we previously demonstrated that ENL abundance rebounds to normal levels 24 h after washout of SR-1114 (ref. ^26^), we can conclude that the less durable degradation of ENL by SR-1114 (24 h versus 5 days for dHTC1) is because of its inferior stability in cell culture—a known liability of the glutarimide scaffold in many CRBN degraders^56^.

Next, we evaluated the pharmacokinetic behavior of dHTC1 in mice, finding that it shows a highly favorable profile for in vivo experimentation (Fig. 4b, Extended Data Fig. 8h,i and Supplementary Fig. 1c,d). After delivery by intraperitoneal injection, a single 50 mg kg^−1^ dose of (*S*)-dHTC1 produced a maximal plasma concentration (*C*_max_) of nearly 50 µM within 20 min (2.5 µM adjusted for 94.9% plasma protein binding, PPB) (Fig. 4b). This exposure of dHTC1 greatly exceeds its DC_50_ value in MV4;11 cells, whereas the same dose of SR-1114 produces a *C*_max_ that only narrowly reaches its PPB-adjusted DC_50_ in MV4;11 cells (PPB = 93.9%). These data indicate that dHTC1 afforded a probe with more suitable properties for experimentation in vivo compared to a similarly unoptimized PROTAC.

To assess pharmacodynamics, we used an MV4;11 orthotopic xenotransplantation model of AML and observed stereoselective ENL degradation after three administrations of (*R*)-dHTC1 or (*S*)-dHTC1 (50 mg kg^−1^) (Fig. 4c,d). (*S*)-dHTC1 stereoselectively inhibited the expression of ENL target genes (Fig. 4d–f and Extended Data Fig. 8j), as defined by the binding of ENL to gene promoters in chromatin immunoprecipitation and sequencing (ChIP-seq) experiments or by their responsiveness to dTAG-mediated degradation^54^. Consistent with the prodifferentiation effects of ENL inhibitors and degraders on leukemia cells^54,57,58^, we also noted an increase in transcription of the myeloid differentiation marker *ITGAM* (encoding CD11b), which was confirmed by measuring cell surface expression of CD11b in the spleen and peripheral blood (Fig. 4g). Altogether, these data demonstrate that HTC diversification can deliver CIPs with properties that are useful for in vivo studies.

### HTC-based conversion of BRD4 ligands into degraders

**Fig. 5:**
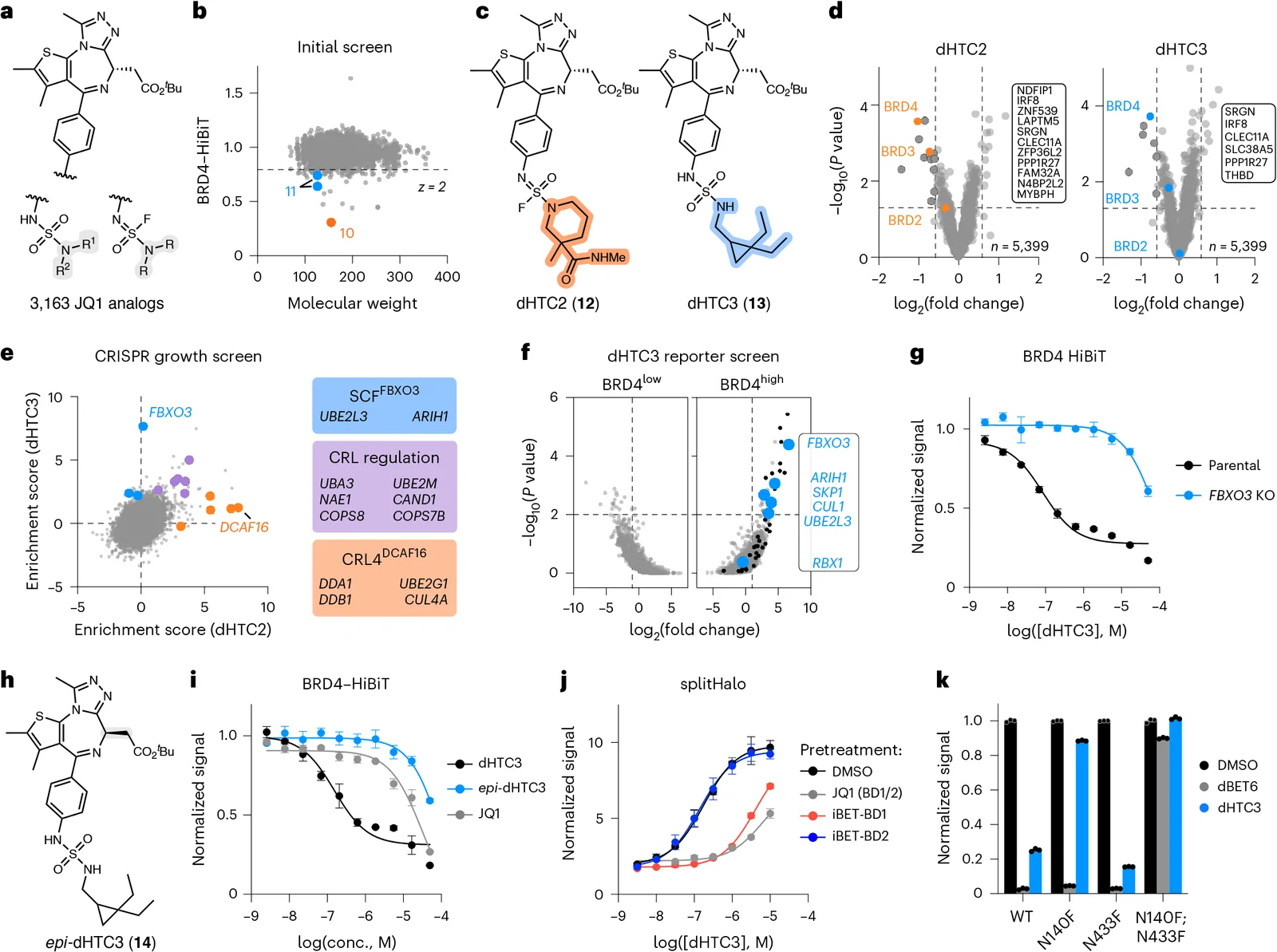
HTC reveals an FBXO3-dependent and BD1-specific BRD4 degrader. **a**, Structure of JQ1 analogs synthesized by HTC. Products were formed as a 9:1 mix of enantiomers (*S*:*R*). **b**, Crude reaction products were screened for BRD4 degradation (2 µM, 16 h) in MV4;11 cells expressing BRD4–HiBiT. HiBiT luminescence is normalized to DMSO:PBS vehicle control (single independent experiment). Molecular weight is shown for the free amine building block. **c**, Structure of dHTC2 (**12**) and dHTC3 (**13**). **d**, Quantitative TMT-based expression proteomics analysis of MV4;11 cells treated with dHTC2 and dHTC3 (1 µM, 16 h) or DMSO vehicle control (5,399 proteins with >2 peptides depicted; data were filtered using DTAselect 2.0 within IP2). *P* values were calculated using a two-tailed Student’s *t*-test, (*n* = 3 biological replicates). **e**, Gene effect scores in CRISPR–Cas9-based KO screen (18,993 genes) in NALM6 cells treated with either dHTC2 (150 nM) or dHTC3 (400 nM). **f**, FACS-based CRISPR screens for UPS components affecting BRD4 stability in KBM7 reporter cells (KBM7 iCas9 BRD4s–TagBFP–P2A–mCherry) treated with DMSO or dHTC3 at 2 µM for 16 h (six sgRNAs targeting 1,301 UPS-associated genes). Gene-level fold change and *P* values were determined by one-sided MAGeCK analysis^114^. Members of the 26S proteasome are indicated in black. **g**, HEK293T BRD4–HiBiT cells with and without *FBXO3* KO treated with dHTC3 for 16 h. HiBiT luminescence normalized to DMSO vehicle control (mean ± s.e.m.; *n* = 3 biological replicates). **h**, Structure of *epi*-dHTC3. **i**, MV4;11 BRD4–HiBiT cells treated for 16 h. HiBiT luminescence was normalized to the DMSO vehicle control (mean ± s.e.m.; *n* = 3 biological replicates). **j**, RPE1 cells ectopically expressing FBXO3–GFP–cpHalo and Hpep6–BRD4s–EF1as–TagBFP constructs were pretreated with carfilzomib and the indicated compounds (20 µM) or DMSO control for 1 h and then treated with dHTC3 for the dose–response assay and with 100 nM TAMRA-CA for 3 h before flow cytometry analysis. Results are presented as the ratio of TAMRA to cpHalo and then normalized to DMSO (mean ± s.e.m.; *n* = 3 biological replicates from two independent experiments). **k**, Degradation of BRD4 with WT and mutant bromodomains. KBM7 iCas9 cells expressing the tandem bromodomains of BRD4 (BD1–BD2) tagged with TagBFP–P2A–mCherry were treated with DMSO, 10 nM dBET6 or 2 µM dHTC3 for 16 h. The signal is BFP–mCherry, normalized to DMSO-treated cells (mean ± s.e.m.; *n* = 3 biological replicates from two independent experiments). Source data

**Extended Data Fig. 9:**
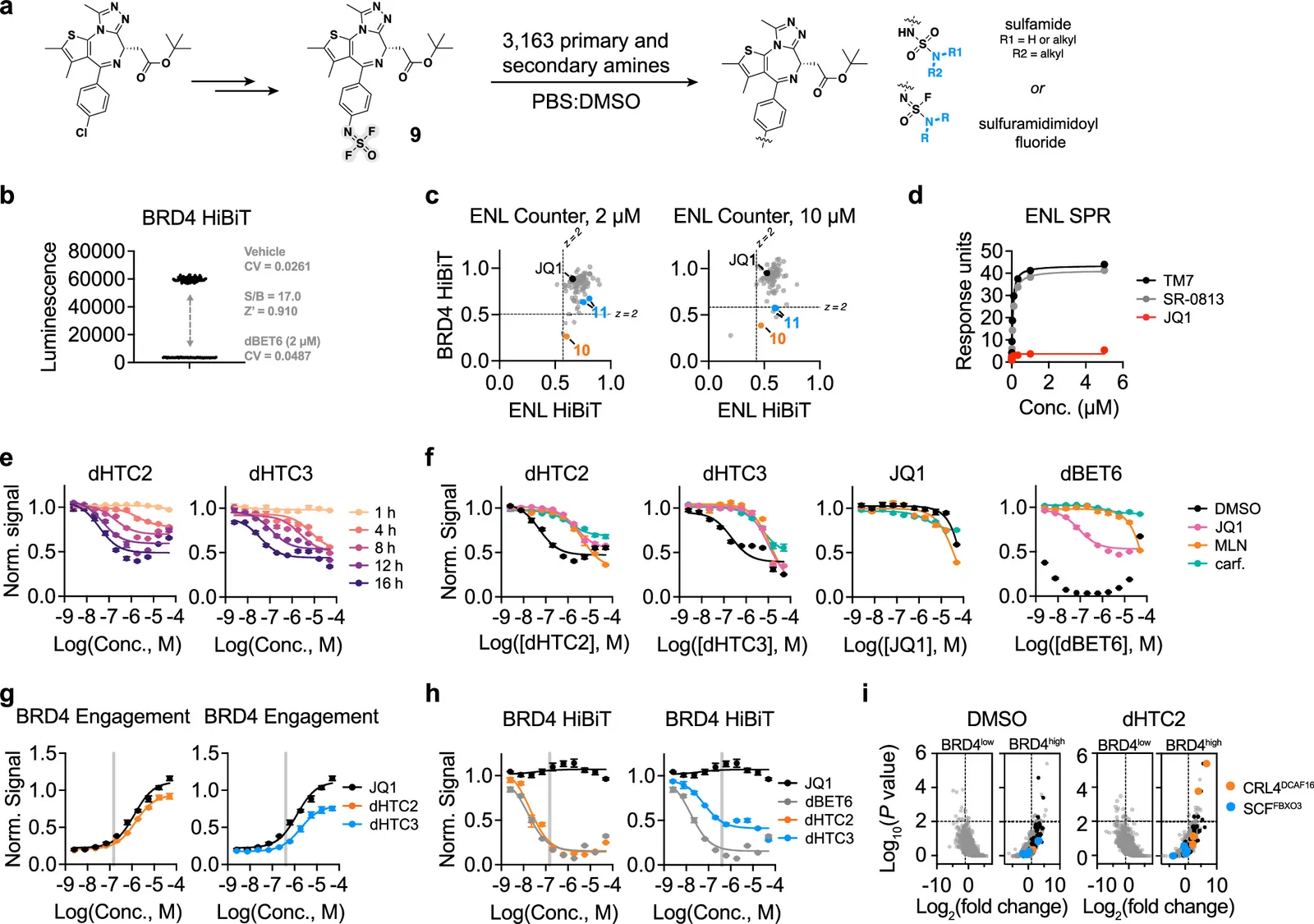
Discovery and validation of dHTC2 and dHTC3 BRD4 degraders. **a**, JQ1 was functionalized with an iminosulfur oxydifluoride SuFEx handle and used for parallel synthesis of 3,163 analogs. Products were formed as a 9:1 mix of enantiomers (*S*:*R*). **b**, Assay performance of MV4;11-BRD4-HiBiT cells for BRD4 degradation. Data is raw luminescence in MV4;11-BRD4-HiBiT cells treated with vehicle or dBET6. **c**, Fig. 5b hits were rescreened at 2 or 10 µM (16 h) in MV4;11-BRD4-HiBiT cells, counter screened against MV4;11-HiBiT-ENL cells (HiBiT luminescence normalized to DMSO:PBS vehicle control, 3 biological replicates). **d**, SPR analysis of binding to immobilized ENL-YEATS (mean of 2 technical replicates). Same experiment as Extended Data Fig. 1c (TM7 and SR-0813 repeated). **e**, Time-dependent and dose-responsive loss of BRD4-HiBiT signal in MV4;11 cells treated with **12** and **13**. Luminescence normalized to DMSO (mean of 4 biological replicates ± S.E.M.). **f**, Dose-responsive effects of dHTC2, dHTC3, JQ1, and dBET6 (12 h treatment) in MV4;11 BRD4 HiBiT cells following indicated pretreatments (1 h). BRD4-HiBiT luminescence normalized to DMSO vehicle control (mean of 4 biological replicates ± S.E.M.). Cells are pretreated with DMSO, JQ1 (10 µM), MLN4924 (1 µM) or carfilzomib (500 nM). **g-h**, BRD4 degradation and target engagement assays were performed to select concentrations for growth-based CRISPR knockout screen presented in Fig. 5e. Left, target engagement by rescue of dBET6-induced BRD4-HiBiT degradation. NALM6-BRD4-HiBiT cells were treated with the indicated compounds in dose response for 1 h, prior to dBET6 treatment (500 nM final concentration) for 1 h (mean of 4 biological replicates ± S.E.M., normalized to DMSO treated cells). Right, a 16 h dose-responsive loss of BRD4-HiBiT signal in NALM6 cells treated with **12** and **13** (HiBiT luminescence normalized to DMSO vehicle control, mean of 4 biological replicates ± S.E.M.). Gray bands indicate concentrations selected for genetic screen, which induce near-maximal degradation with minimal BRD4 engagement. **i**, FACS-based CRISPR screens for UPS components affecting BRD4 stability in KBM7 reporter cells (KBM7 iCas9 BRD4s-TagBFP-P2A-mCherry) treated with DMSO or dHTC2 at 2 µM for 16 h (6 sgRNAs targeting 1301 UPS-associated genes). Gene-level fold change and *P* values determined by one-sided MAGeCK^114^ analysis. 26S proteasome members are indicated in black. Source data

To evaluate the extensibility of this approach, we sought to convert JQ1, a potent and selective BET (BRD2, BRD3 and BRD4) bromodomain ligand^59^, into molecular glue degraders, several of which have been reported recently^23–25,34,60,61^. JQ1 was first converted to an anilino intermediate through Buchwald–Hartwig amination assisted by a newly reported Pd(COD)(DQ) catalyst^62^, which was then furnished with an iminosulfur oxydifluoride SuFEx hub to yield JQ1-difluoride (**9**) facilitated by a silver pentafluorooxosulfate salt (Extended Data Fig. 9a)^63^. Using the same 3,163 amines that were previously used to discover dHTC1, we synthesized more than 3,000 JQ1 analogs and screened each for the ability to induce BRD4–HiBiT degradation (Fig. 5a,b, Extended Data Fig. 9b and Supplementary Table 11). This produced 82 hits (Fig. 5b), which were retested in triplicate and counterscreened against HiBiT–ENL, as JQ1 does not bind to ENL (Extended Data Fig. 9c,d and Supplementary Table 12). We prioritized two building blocks for follow-up studies, nipecotamide JS-2 (**10**) and cyclopropylmethanamine JS-3 (**11**), the latter of which was unintentionally included in the library twice (Fig. 5b and Extended Data Fig. 9c). These were used to synthesize and purify dHTC2 (**12**) and dHTC3 (**13**), which were confirmed by high-resolution MS and nuclear magnetic resonance (NMR) to be formed by sulfuramidimidoyl fluoride and sulfamide connections, respectively (Fig. 5c). Both compounds induced selective and CRL-dependent degradation of BRD4 (Fig. 5d, Extended Data Fig. 9e,f and Supplementary Table 13). Blockade of BRD4–HiBiT degradation by MLN4924 and carfilzomib was not observed at higher concentrations approaching 10 µM, which we suspect is because of the transcriptional consequences of BET bromodomain inhibition, as JQ1 itself decreases BRD4–HiBiT signal at these high concentrations (Extended Data Fig. 9f). We cannot rule out other explanations, including off-target effects at high concentrations of dHTC2 and dHTC3. Nevertheless, the full rescue of BRD4–HiBiT levels at lower concentrations of dHTC2 and dHTC3 demonstrate that their activity is CRL dependent, motivating effector discovery by forward genetics.

### F-box protein 3 mediates BRD4 degradation by dHTC3

We performed two orthogonal CRISPR screens to identify the cellular effectors of BRD4 degradation. In the first screen, we leveraged the toxicity of BRD4 degradation to perform a growth-based drug resistance screen in NALM6 cells. As BET degradation elicits stronger antiproliferative effects than bromodomain inhibition^46,64^, we selected concentrations of each compound that are sufficient to induce BRD4 degradation but show minimal engagement of BRD4 bromodomains (Extended Data Fig. 9g,h). From a genome-scale sgRNA library, we primarily identified members of the UPS, with CRL regulators being shared as hits by both dHTC2 and dHTC3 (Fig. 5e). Subunits of CRL4^DCAF16^ (DDB1-associated and Cullin 4-associated factor 16) were identified only for dHTC2, whereas F-box protein 3 (FBXO3), a substrate receptor of the Skp1–Cullin–F-box (SCF) complex, was identified only for dHTC3 (Fig. 5e). The SCF regulators, ubiquitin-conjugating enzyme E2 L3 (UBE2L3) and Ariadne RBR E3 ubiquitin protein ligase 1 (ARIH1)^65,66^, were also enriched by dHTC3, albeit to a weaker extant than FBXO3.

**Extended Data Fig. 10:**
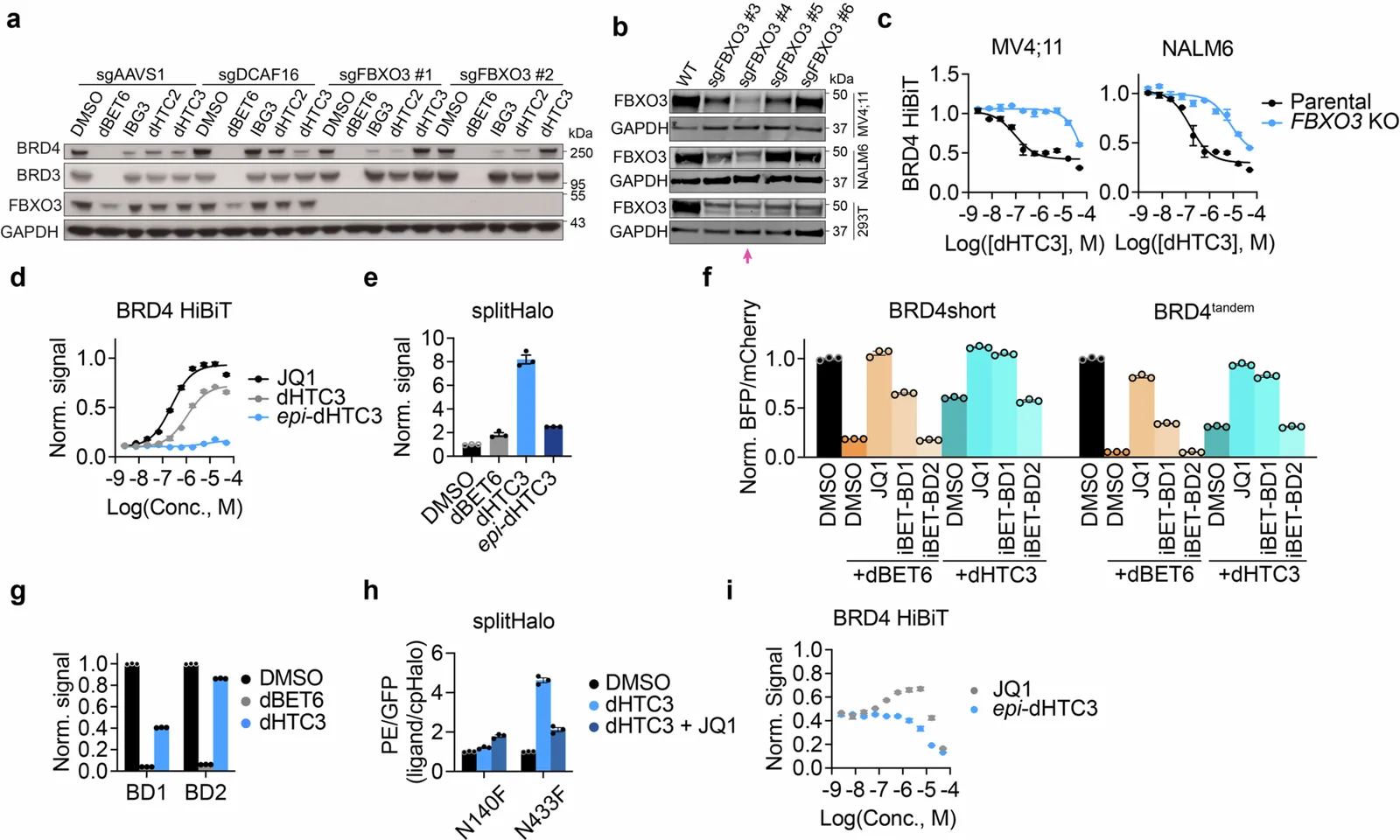
dHTC3 induces BRD4 degradation through BD1. **a**, KBM7-iCas9-BRD4(TandemBD)-TagBFP-P2A-mCherry reporter cells transduced with sgRNAs targeting indicated genes and co-expressing iRFP720 to identify sgRNA-positive cells. Cas9 was induced with doxycycline 4 days before treatment. Cells treated with DMSO, 10 nM dBET6, 100 pM IBG3, 2 µM dHTC2, or 10 µM dHTC3 (16 h). Representative of 2 independent experiments. **b**, CRISPR/Cas9 RNPs containing one of four guides delivered to cells by electroporation. sgFBXO3 #4, was selected for further studies. Single experiment. **c**, Left, MV4;11-BRD4-HiBiT and, right, NALM6-BRD4-HiBiT cells (parental) or those bearing an *FBXO3* knockout treated with dHTC3 (16 h). BRD4-HiBiT luminescence normalized to DMSO-treated cells (mean, 4 biological replicates). **d**, MV4;11-BRD4-HiBiT were pre-treated with compounds (1 h) prior to dBET6 treatment (1 h, 500 nM). HiBiT luminescence normalized to DMSO (mean, 4 biological replicates). **e**, SplitHalo assays in RPE1 cells ectopically expressing FBXO3-GFP-cpHalo and Hpep6-BRD4s-EF1αs-TagBFP constructs. Cells pre-treated with carfilzomib (1 h), and then treated with indicated compounds (10 nM dBET6, 2 µM dHTC3 and 2 µM *epi*-dHTC3, 3 h) and 100 nM TAMRA-CA. GFP/BFP double-positive cells were gated, SplitHalo signal (TAMRA, PE channel) was normalized to cpHalo expression (GFP signal) to account for expression-level variation. Data shown as TAMRA/GFP ratios, normalized to DMSO-treated control. **f**, KBM7-iCas9 cells expressing BRD4short or tandem BD1-BD2 bromodomains (BRD4^tandem^) in TagBFP-P2A-mCherry vector were pre-treated (1 h) with indicated compounds (20 µM) before dBET6 (10 nM) or dHTC3 (2 µM) (8 h). Signal (BFP/mCherry ratio) is normalized to DMSO-treated cells. **g**, KBM7-iCas9 cells expressing BRD4 BD1 or BD2 (with BRD4 nuclear localization sequence) fused to TagBFP-P2A-mCherry were treated with DMSO, 10 nM dBET6, or 2 µM dHTC3 (16 h). BFP/mCherry ratios are normalized to DMSO-treated cells. **h**, SplitHalo assays conducted in RPE1 cells (same as panel e) expressing BRD4(S) with BD1 (N140F) or BD2 (N433F) mutants. Cells pre-treated with carfilzomib and either DMSO or 10 µM JQ1 (1 h), followed by DMSO or 2 µM dHTC3 (3 h) with 100 nM TAMRA-CA. **i**, Rescue of dHTC3-mediated BRD4 degradation. HEK293T-BRD4-HiBiT cells were pre-treated with compounds (1 h) prior to 16 h treatment with dHTC3 (1 µM, mean, 4 biological replicates). **e-h** mean, 3 biological replicates, 2 independent experiments. **c-i** ± S.E.M. Source data

In parallel, we performed a FACS-based CRISPR screen using a library of UPS-focused sgRNAs and a dual fluorescence stability reporter for the short isoform of BRD4, BRD4(S) (KBM7 cells expressing a BRD4(S)–TagBFP–P2A–mCherry construct) (Fig. 5f and Extended Data Fig. 9i)^33–36^. This experiment independently confirmed CRL4^DCAF16^ and SCF^FBXO3^ as being responsible for the activity of dHTC2 and dHTC3, respectively. While FBXO3 was the strongest hit for dHTC3, this screen also identified UBE2L3, ARIH1 and the SCF subunits, Cullin and Skp1. The latter two, which are essential genes, were uniquely discovered by the reporter-based screen, highlighting its ability to query genes that are required for cell survival^33–36^. We validated the results for dHTC2 using previously reported *DCAF16*-KO KBM7 cells (Extended Data Fig. 10a)^34^. To validate FBXO3 as the effector for dHTC3, we introduced *FBXO3* KO in KBM7, MV4;11, NALM6 and HEK293T cells, each of which were resistant to dHTC3 (Fig. 5g and Extended Data Fig. 10a–c).

### dHTC3 is a BD1-specific BRD4 degrader

As DCAF16 is already reported to support targeted protein degradation^67^ and several DCAF16-dependent BRD4 degraders are already known^23,25,34^, we focused our subsequent efforts on dHTC3 and SCF^FBXO3^. We first synthesized *epi*-dHTC3 (**14**) with a stereochemical inversion known to disrupt BET bromodomain engagement^59^ and confirmed that it could not induce BRD4 degradation (Fig. 5h,i and Extended Data Fig. 10d). We also developed a splitHaloTag-based proximity assay for FBXO3 and BRD4 to interrogate ternary complex formation in cells^68^, observing a stereoselective increase in splitHaloTag signal in the presence of dHTC3 that could be suppressed by excess JQ1 (Fig. 5j and Extended Data Fig. 10e).

To further probe this mechanism of ternary complex formation, we tested whether dHTC3 activity could be blocked by iBET-BD1 or iBET-BD2, bromodomain inhibitors that selectively bind to BD1 or BD2 of BET proteins, respectively^69^. Only iBET-BD1 could block both dHTC3-induced ternary complex formation and BRD4 degradation (Fig. 5j and Extended Data Fig. 10f), despite the ability of dHTC3 to engage both bromodomains, as shown by its blockade of dBET6-induced BRD4 degradation (Extended Data Fig. 10d). In agreement with these data, truncated constructs of BRD4 (ref. ^34^) showed that dHTC3 could only degrade BD1 fusions, whereas the PROTAC dBET6 could degrade BD1 and BD2 fusions (Extended Data Fig. 10g). Likewise, dHTC3 failed to induce degradation of a tandem bromodomain construct when it harbored an N140F substitution that blocks binding to BD1 (Fig. 5k)^70^, further confirming it cannot induce degradation through BD2. To determine whether dHTC3 engagement of BD2 is unable to induce ternary complex formation or, alternatively, leads to the formation of a ternary complex that is nonproductive for targeted protein degradation, we again leveraged the splitHaloTag assay. Using BRD4(S) constructs harboring an N140F or N433F substitutions, we found that dHTC3 could only mediate ternary complex formation with BD1 (Extended Data Fig. 10h).

Lastly, we note that dHTC3 did not show a hook effect in BRD4 degradation or BRD4–FBXO3 ternary complex formation assays, providing a preliminary suggestion that it may engage FBXO3 cooperatively through a glue-like mechanism of action (Fig. 5j and Extended Data Fig. 9e). To assess whether this scaffold can engage FBXO3 independently, we tested whether *epi*-dHTC3, which cannot bind to BRD4, could block dHTC3-induced degradation of BRD4 (Extended Data Fig. 10i). Its inability to block dHTC3 suggests that FBXO3 is not independently liganded by dHTC3 and is likely recruited only to the preformed BRD4(BD1)–dHTC3 complex.

## Discussion

While we and others have shown that HTC can be used to reshape the neosubstrate scopes of E3 ligase ligands^13,30,71–79^, a generalizable solution for the target-centric discovery of molecular glues has remained elusive. Satisfied that ligand binding can, in principle, create surface alterations that cooperatively stabilize a defined protein–protein interaction, we sought a method to discover molecular glues by systematically altering a predefined protein–ligand surface at scale. This was principally inspired by the report of BCL6 and CDK12/cyclin K molecular glue degraders, which were retrospectively found to stabilize protein–protein interactions through surficial ligand modifications absent in their nonglue analogs^18–22,80^. However, as BCL6 and CDK12/cyclin K appear uniquely susceptible to molecular glue degraders (nearly half of their ligands are also degraders^18,80,81^), it was unclear whether these examples would be generally instructive.

Previous reports of molecular glues that first bind to a target and then cooperatively engage an E3 ligase have largely relied on serendipity or target-agnostic screens^18–22,82–84^. HTC proved essential for converting ligands into molecular glues, as we found that relatively few modifications could endow ligands with this neomorphic activity. In contrast, previous studies have demonstrated that CIPs can be discovered from a much smaller collection of analogs by appending simple electrophilic handles directly onto a ligand of interest^23,24^. These covalent handles can be transplanted onto ligands of diverse targets to afford effective degraders, as they possess intrinsic reactivity for the E3 ligase^23,24^. A differentiating feature of this HTC-based approach is the ability, embodied by dHTC1, to prospectively discover noncovalent molecular glues that rely on interfacial protein–protein contacts that cooperatively stabilize a ternary complex. However, it is also conceptually possible to discover heterobifunctionals using HTC (dHTC2 shows a strong hook effect in degradation assays) and further study is required to determine whether a hit behaves as a molecular glue or heterobifunctional.

SuFEx provided a favorable method for high-throughput ligand diversification, given (1) the wide commercial availability of SuFEx-compatible amine building blocks; (2) the facile and scalable synthetic procedures used for large library builds; and (3) the ability to perform an effector-agnostic screen by testing crude reaction products directly in native living systems. However, as the sulfamide of dHTC1 was essential for its activity, other chemistries will likely yield different hits and it is, therefore, encouraging that additional HTCs are available^85–89^. DNA-encoded library approaches have identified bifunctional compounds with glue-like cooperativity but they require preselection of both target and effector^90,91^. It is notable that neither the mechanism of dHTC1 nor the effector for dHTC3 would have been anticipated in advance of these screens, highlighting an advantage of effector-agnostic screening in cells. Furthermore, we envision that HTC could be adapted to cell-based screening assays for neomorphic pharmacology well beyond targeted protein degradation.

Initially, it seemed improbable to screen over 3,000 analogs only to discover a compound operating through the well-established effector, CRBN, especially one with minimal independent affinity for the ligase. However, this finding may inform whether some ligases are more easily co-opted than others. Genetic screens using fusion protein constructs have evaluated this question en masse, comparing hundreds of proteins for their ability to stimulate protein degradation^92,93^. Like CRBN, FBXO3 scored highly in these screens, suggesting that it may be similarly well disposed to proximity pharmacology^92,93^. However, several effectors (for example, CRBN, DCAF11, DCAF16, FBXO22 and tripartite motif-containing protein 21 (TRIM21)) have been overrepresented in reports of small-molecule degraders^23,25,34,36,60,67,94–101^, despite some, like DCAF16, scoring poorly in previous genetic screens^92,93^. It is interesting to consider other characteristics that might produce these ‘frequent hitters’, such as their intrinsic ligandability, covalent reactivity or their overrepresentation in the cellular repertoire of CRL complexes, among other possibilities^102^. Furthermore, while we did not observe interactions between ENL and 1 µM CRBN, we cannot rule out the possibility that they interact at concentrations higher than those we were able to assess. If so, dHTC1 may exploit a weak native interaction between ENL and CRBN to form a stable ternary complex, which is a common feature of diverse molecular glues^103^.

One hypothesis is that some proteins may have evolved the capacity to promiscuously engage many interaction partners. CRBN recognizes proteins with C-terminal cyclic imide modifications that are formed from the cyclization of asparagine and glutamine on damaged proteins^38^. This mechanism of degron recognition might suggest that CRBN is poised to bind a large fraction of the proteome, provided that it is presented with a cyclic imide modification for recognition^38^—a hypothesis that would be supported by the conditional engagement of CRBN by dHTC1. Our structural studies indicate that CRBN makes concerted movements to accommodate the induced ENL interaction with a high degree of shape complementarity, which is congruent with emerging studies demonstrating that the CRBN surface is highly plastic and capable of accommodating diverse structural degrons^42,104–106^.

On the basis of these data, we might envision efforts to decorate ligands with diverse cyclic imide moieties, regardless of their intrinsic affinity for CRBN, with the intent of discovering protein–ligand complexes that are biased for CRBN capture without necessarily being biased for CRBN affinity. As the degradation of CRBN neosubstrates can be a major concern in the development of CRBN-based degraders^16,107–109^, a lack of independent CRBN affinity is likely a benefit that minimizes the chance of eliciting off-target neosubstrate degradation, as we see for dHTC1. This selectivity and the availability of an inactive enantiomeric control position (*S*)-dHTC1 favorably as a chemical probe for the study of ENL function in cellular and animal model systems. Notably, such a stereoisomeric control is difficult to develop with thalidomide-based degraders, which are often prone to fast racemization in situ^110^.

Unlike CRBN, which has a central role in the development of targeted protein degradation, FBXO3 has not been co-opted by small molecules. Several observations point toward dHTC3 functioning as a molecular glue, most notably the lack of an apparent hook effect by dHTC3 and the inability of *epi*-dHTC3 to block its activity, as well as its unexpected selectivity for BD1 over BD2. We hypothesize that dHTC3 must first engage BRD4 BD1 before engaging FBXO3. However, structural studies will be required to definitively assess this question and molecular glue activity would not rule out the existence of a ligandable pocket on FBXO3. Prior studies have identified FBXO3 as a regulator of inflammatory signaling^111–113^, suggesting that, in the future, it may perhaps be possible to discover FBXO3-dependent degraders that are conditionally activated by inflammatory cell states.

## Methods

### Chemical synthesis

A detailed description of chemical syntheses, spectroscopic characterization and relevant chiral characterization, as well as flow cytometry gating strategies and SPR sensorgrams, is provided in Supplementary Methods.

### Cell lines and culture

HL-60 cells (gift from the laboratory of J. Bradner), KBM7 cells (gift from the laboratory of T. Brummelkamp) and KBM7 cells harboring a doxycycline-inducible Cas9 (iCas9; gift from the laboratory of J. Zuber) were cultured in 10% FBS in Iscove’s modified Dulbecco’s medium (IMDM). OCI-AML2 (gift from J. Bradner), OCI-AML3 (German Collection of Microorganisms and Cell Cultures (DSMZ), ACC 582) and Kasumi-1 (American Type Culture Collection (ATCC), CRL-2724) were cultured in 20% FBS in RPMI 1640. HB11;19 (gift from the laboratory of A. Yokoyama), Jurkat (gift of the laboratory of M. Bollong) MV4;11 and MOLM-13 (gifts from J. Bradner), NALM6 (given by ChemoGenix, University of Montreal) and EOL1 (DSMZ, ACC 386) cells were cultured in 10% FBS in RPMI 1640. RPE1 (ATCC, CRL-4000), HEK293T (ATCC, CRL-3216) and Lenti-X 293T (Takara Bio, 632180) cells were cultured in 10% FBS in DMEM. All cell lines were confirmed to be free of *Mycoplasma* at 1-month intervals and never tested positive. All cell lines were cultured in media using heat-inactivated FBS, supplemented with streptomycin and penicillin, and stored in a 37 °C incubator at 5% CO_2_.

For lentiviral production, Lenti-X 293T cells were seeded in six-well plates or 10-cm dishes and transfected at approximately 80% confluency with 0.6/3.4 μg of target plasmid, 1.7/0.3 μg of psPAX2 (Addgene, 12260) and 0.85/0.15 μg of pMD2.G (Addgene, 12259) using polyethylenimine (PEI; PolySciences). Virus supernatant was collected after 60 h, filtered using a 0.45-μm filter and stored in aliquots for transduction.

### Viability studies

To assess drug response, 20 × 10^4^ MV4;11 or HL-60 cells were seeded per well in a non-tissue-culture-treated 96-well plate (VWR) and treated with 0.1% DMSO or increasing concentrations of (*R*)-dHTC1 or (*S*)-dHTC1 (39.07 nM, 78.13 nM, 156 nM, 312 nM, 625 nM, 1.25 µM, 2.5 µM, 5 µM and 10 µM). Cells were split 1:10 and fresh drug was added every 3 days. Cell proliferation was measured on day 12 using the CellTiterGlo luminescent cell viability assay (Promega). Drug response was calculated on the basis of luminescence relative to the DMSO-treated samples. The experiment was independently repeated three times in technical replicates and, for each, the average luminescence was calculated. Diagrams show the mean of the three experiments and error bars represent the s.e.m.

### HiBiT–ENL cell lines

The following cell lines were generated bearing an endogenously tagged N-terminal HiBiT–ENL with a GSG tripeptide linker through homology-directed repair: OCI-AML2, OCI-AML3, MV4;11 (WT and *CRBN*-KO^30^), MOLM-13, EOL1 and HL-60. All components necessary were ordered from Integrated DNA Technologies (IDT). A Neon transfection system (Thermo Fisher) was used to electroporate cells. Briefly, sgRNA complexes were synthesized by mixing equal volumes of crRNA (2 µl, 160 µM in nuclease-free water, ggcgccagccatggacaatc) and tracrRNA (2 µl, 160 µM) and incubated at 37 °C for 30 min before Cas9 (2.7 µl, 10 µg µl^−1^, Alt-R SpCas9 nuclease V3, 1081058) and duplex buffer (1.3 µl; 30 mM HEPES pH 7.5 and 100 mM potassium acetate in nuclease-free water) were added. This suspension was incubated at 37 °C for an additional 15 min to form the active RNP complex. The RNP solution (1 µl) was added to single-stranded oligodeoxynucleotide (ssODN; 2 µM in nuclease-free water, gcggcggcggcgagcgacgcggggcccgggggcggggcggggcgccagccatggtgagcggctggcggctgttcaagaagattagcggaagcggagacaatcaagtgaggagcggccgcgccgcccctgcgcagcccgcccggccccct). The resulting mixture is electroporated with 200,000 cells in electroporation buffer and the cells are deposited into antibiotic-free medium, as described above, and allowed to rest in a 37 °C incubator for 24 h before additional antibiotic-containing medium was added. Replicates with the highest overall signal were selected and expanded.

The initial screen (Fig. 1b) was run in MV4;11 cells bearing an N-terminal tagged ENL–HiBiT tag without a GSG linker. Subsequent assays used a redeveloped cell line bearing this tripeptide linker as we found the original HiBiT reporter signal diminished after serial passaging in cell culture. The resulting HiBiT–GSG–ENL reporter (hereafter referred to as HiBiT–ENL) showed improved durability and overall assay performance and was, therefore, used for all subsequent studies. For MV4;11 cells expressing HiBiT–ENL without a GSG linker, the following ssODN was ordered from IDT and used as received: gcggcggcggcgagcgacgcggggcccgggggcggggcggggcgccagccATGGTGAGCGGCTGGCGGCTGTTCAAGAAGATTAGCGACAccgcccggccctccccggtcccggcctccccgctccgcgcccgcccgccc.

The electroporation conditions for the MV4;11, OCI-AML2, OCI-AML3, MV4;11, MOLM-13 and EOL1 cell lines were as follows: pulse voltage, 1,600 V; pulse width, 10 ms; number of pulses, 3. The electroporation conditions for the HL-60 cell line were as follows: pulse voltage, 1,350 V; pulse width, 35 ms; number of pulses, 1.

### BRD4–HiBiT cell lines

All components necessary were ordered from IDT. A Neon transfection system (Thermo Fisher) was used to electroporate cells. Briefly, sgRNA complexes were synthesized by mixing equal volumes of crRNA (2 µl, 160 µM in nuclease-free water, aatcttttctgagcgcacct) and tracrRNA (2 µl, 160 µM) and incubated at 37 °C for 30 min before Cas9 (2.7 µl, 10 µg µl^−1^; Alt-R SpCas9 nuclease V3, 1081058) and duplex buffer (1.3 µl; 30 mM HEPES pH 7.5 and 100 mM potassium acetate in nuclease-free water) were added. This suspension was incubated at 37 °C for an additional 15 min to form the active RNP complex. The RNP solution (1 µl) was added to ssODN (2 µM in nuclease-free water, catgaatttccagagtgatctattgtcaatatttgaagaaaatcttttcggtggcggtggctcgggcggtggtgggtcgggtggcggcggatctgtgagcggctggcggctgttcaagaagattagctgacctaggtggcttctgactttgattttctggcaaaacattgactttccata). All oligonucleotides were acquired from IDT and used as received. The resulting mixture was electroporated (pulse voltage, 1,600 V; pulse width, 10 ms; number of pulses, 3) with 200,000 cells in electroporation buffer and the cells were deposited into antibiotic-free medium, as described above, and allowed to rest in a 37 °C incubator for 24 h before additional antibiotic-containing medium was added. Replicates with the highest overall signal were selected and expanded.

For MV4;11 and NALM6 cells, the mixture was electroporated with the following conditions: pulse voltage, 1,600 V; pulse width, 10 ms; number of pulses, 3. For HEK293T cells, the mixture was electroporated with the following conditions: pulse voltage, 1,500 V; pulse width, 30 ms; number of pulses, 1.

### MV4;11 PARP1–HiBiT cell lines

The procedure above was adapted to generate MV4;11 cells with a C-terminal HiBiT-tagged PARP1. The crRNA used was taagacctccctgtggtaat and the ssODN used was tctgaagtatctgctgaaactgaaattcaattttaagacctccctgtggggaagcggagTAAGCGGCTGGCGGCTGTTCAAGAAGATTAGCTAAaattgggagaggtagccgagtcacacccggtggctctggtatgaattcac. All oligonucleotides were acquired from IDT and used as received.

### *FBXO3*-KO cell lines

All components necessary were ordered from IDT. sgRNA complexes were synthesized by mixing equal volumes of crRNA (2 µl, 160 µM in nuclease-free water) and tracrRNA (2 µl, 160 µM) and incubated at 37 °C for 30 min before Cas9 (2.7 µl, 10 µg µl^−1^; Alt-R SpCas9 nuclease V3, 1081058) and duplex buffer (1.3 µl; 30 mM HEPES pH 7.5 and 100 mM potassium acetate in nuclease-free water) were added. This suspension was incubated at 37 °C for an additional 15 min to form the active RNP complex. The RNP solution (1 µl) was electroporated with 200,000 cells in electroporation buffer and the cells were deposited into antibiotic-free medium, as described above, and allowed to rest in a 37 °C incubator for 24 h before additional antibiotic-containing medium was added.

For MV4;11 and NALM6 cells, the mixture was electroporated with the following conditions: pulse voltage, 1,600 V; pulse width, 10 ms; number of pulses, 3. For HEK293T cells, the mixture was electroporated with the following conditions: pulse voltage, 1,500 V; pulse width, 30 ms; number of pulses, 1.

The following *FBXO3* crRNA guides were ordered from IDT and used:

sgFBXO3-3: GGAGGATTCCAGCAGAGACA

sgFBXO3-4: TATCAAGTCATGATCCGCTG

sgFBXO3-5: TGTTCATACCGAATTCACAA

sgFBXO3-6: GACGTCGATACAGCTGCCGG

### Endpoint HiBiT protein degradation

HiBiT reporter cells, generated as described above, were seeded into 384-well plates at 20,000 cells per well and treated with the indicated compounds and equivalent concentrations of DMSO for the time indicated in the experiment, usually 3, 24 or 72 h. Levels of HiBiT-tagged proteins were detected by luminescence using the Nano-Glo HiBiT lytic detection system (Promega, N3050). Dose–response curves were plotted in GraphPad (log(inhibitor) versus response (three parameters)) and the data shown were from three or four independent repeats (mean ± s.e.m.).

### ENL intracellular target engagement assay

Intracellular ENL engagement assays were carried out as described previously^116^. Briefly, an Echo acoustic liquid handler (Labcyte) was used to transfer drug in a ten-point dose–response series (100-nl transfers) to empty wells on a white, tissue-culture-treated 384-well plate. To these wells were added OCI/AML2 cells stably expressing ENL (YEATS)–HiBiT at concentrations of 20,000 cells in 20 µl of medium. The plate was placed on an orbital shaker for 15 s and then placed in a 37 °C incubator for 3 h or a time otherwise indicated, after which each well was treated with 20 µl of Nano-Glo HiBiT lytic detection (Promega, N3050). After this treatment, the plate was mixed with an orbital shaker for 15 s and allowed to rest in the dark for 20 min before luminescence was measured with a Clariostar microplate reader (BMG Labtech). Dose–response curves were plotted in GraphPad (log(inhibitor) versus response (three parameters)) and the data shown are from three or four independent repeats (mean ± s.e.m.).

### CRBN intracellular target engagement assay

An Echo acoustic liquid handler (Labcyte) was used to transfer drug in a ten-point dose–response series (100-nl transfers) to empty wells on a white, tissue-culture-treated 384-well plate. MV4;11 cells stably expressing an endogenous BRD4 with a C-terminal HiBiT^30^ were added at a concentration of 20,000 cells in 18 µl of medium and the plate was mixed on an orbital shaker for 15 s. The plate was placed into a 37 °C incubator for 2 h before 2 µl of a 5 µM dBET6 solution in RPMI with 10% FBS was added to each well and the plate was again placed on an orbital shaker for 15 s (final concentration of dBET6: 500 nM); control wells were treated with an equal volume of DMSO in RPMI with 10% FBS. After 1 h of incubation at 37 °C, each well was treated with 20 µl of Nano-Glo HiBiT lytic detection (Promega, N3050). After this treatment, the plate was mixed with an orbital shaker for 15 s and allowed to rest in the dark for 20 min before luminescence was measured with a Clariostar microplate reader (BMG Labtech). Dose–response curves were plotted in GraphPad (log(inhibitor) versus response (three parameters)) and the data shown are from three or four independent repeats (mean ± s.e.m.).

### BRD4–acetyllysine homogeneous time-resolved fluorescence

BRD4 BD1 homogeneous time-resolved fluorescence (HTRF) assays were performed as previously described^116^. Briefly, assays were executed by combining recombinant protein and synthetic histone peptide in assay buffer (25 mM HEPES pH 7, 20 mM NaCl, 0.2% Pluronic F-127 and 0.05% BSA) with 1 nM LanthaScreen Eu anti-His tag antibody (Thermo Fisher, PV5597) and 8.9 nM SureLight allophycocyanin–streptavidin (PerkinElmer, CR130-100). BRD4 BD1 was used at 10 nM and the BRD4 BD1 assay was performed with 13.3 nM tetra-acetylated H4 (BioVision, 7144-01). Next, 5 μl of combined reagents were dispensed into each well of a 1536-well plate (Greiner HiBase) and the drug was added using an Echo 655 acoustic liquid handler (Beckman). Assays were incubated for 2 h before measurement of signal on a PHERAstar plate reader (BMG Labtech; simultaneous dual emission: excitation = 337 nm, emission 1 = 665 nm, emission 2 = 620 nm). HTRF signals (emission ratio at 665 nm to 337 nm) from DMSO vehicle-treated wells (maximum signal) and no-peptide-control wells (minimum signal) were used to calculate the percentage inhibition for drug-treated wells.

### ENL–CRBN HTRF

First, 100 nM biotinylated ENL YEATS domain, 2 nM Tb (CoraFluor-1)-labeled Streptavidin and 100 nM eGFP–CRBN–DDB1^ΔBPB^ were added in assay buffer (25 mM HEPES–NaOH pH 7.4, 150 mM NaCl, 0.5% (w/v) BSA, 0.05% (v/v) Tween-20 and 3 mM TCEP). The reaction mix was added to a 384-well microplate at a total volume of 15 µl per well. Compounds were dispensed using a D300 (Tecan) to the microplate in triplicates of 24 concentrations ranging from 10 µM to 0 µM and normalized with DMSO. Reactions were incubated at room temperature for 1 h and read using a PHERAstar plate reader (BMG Labtech). TR-FRET ratios were calculated as 520 nm/490 nm. Ratios were plotted using GraphPad Prism (version 10.3.1).

### BRD4 and CRBN intracellular target engagement assays

An Echo acoustic liquid handler (Labcyte) was used to transfer compound in a ten-point dose–response series (100-nl transfers) to empty wells on a white, tissue-culture-treated 384-well plate. MV4;11 cells stably expressing endogenous BRD4 with a C-terminal HiBiT^30^ were added at a concentration of 20,000 cells in 18 µl of medium and the plate was mixed on an orbital shaker for 15 s. The plate was placed into a 37 °C incubator for 1 h before 2 µl of a 5 µM dBET6 solution in RPMI with 10% FBS was added to each well and the plate was again placed on an orbital shaker for 15 s (final concentration of dBET6: 500 nM); control wells were treated with an equal volume of DMSO in RPMI with 10% FBS. After 1 h of incubation at 37 °C, each well was treated with 20 µl of Nano-Glo HiBiT lytic detection (Promega, N3050). After this treatment, the plate was mixed with an orbital shaker for 15 s and allowed to rest in the dark for 20 min before luminescence was measured with a Clariostar microplate reader (BMG Labtech). Luminescence values were normalized to DMSO-treated wells and dose–response curves were fitted using nonlinear regression.

### PARP1 intracellular target engagement assay

An Echo acoustic liquid handler (Labcyte) was used to transfer compound in a ten-point dose–response series (100-nl transfers) to empty wells on a white, tissue-culture-treated 384-well plate. MV4;11 cells stably expressing endogenous PARP1 with a C-terminal HiBiT were added at a concentration of 20,000 cells in 18 µl of medium and the plate was mixed on an orbital shaker for 15 s. The plate was placed into a 37 °C incubator for 2 h before 2 µl of a 2.5 µM SK-575 (MedChem Express) solution in RPMI with 10% FBS was added to each well and the plate was again placed on an orbital shaker for 15 s (final concentration of SK-575: 250 nM); control wells were treated with an equal volume of DMSO in RPMI with 10% FBS. After 4 h of incubation at 37 °C, each well was treated with 20 µl of Nano-Glo HiBiT lytic detection (Promega, N3050). After this treatment, the plate was mixed with an orbital shaker for 15 s and allowed to rest in the dark for 20 min before luminescence was measured with a Clariostar microplate reader (BMG Labtech). Luminescence values were normalized to DMSO-treated wells and dose–response curves were fitted using nonlinear regression.

### FBXO3 intracellular target engagement assay

An Echo acoustic liquid handler (Labcyte) was used to transfer compound in a ten-point dose–response series (100-nl transfers) to empty wells on a white, tissue-culture-treated 384-well plate. HEK293T cells stably expressing endogenous BRD4–HiBiT were added at a concentration of 20,000 cells in 18 µl of medium and the plate was mixed on an orbital shaker for 15 s. The plate was placed into a 37 °C incubator for 1 h before 2 µl of a 10 µM dHTC3 solution in RPMI with 10% FBS was added to each well and the plate was again placed on an orbital shaker for 15 s (final concentration of dHTC3: 1 µM); control wells were treated with an equal volume of DMSO in RPMI with 10% FBS. After 16 h of incubation at 37 °C, each well was treated with 20 µl of Nano-Glo HiBiT lytic detection (Promega, N3050). After this treatment, the plate was mixed with an orbital shaker for 15 s and allowed to rest in the dark for 20 min before luminescence was measured with a Clariostar microplate reader (BMG Labtech). Luminescence values were normalized to DMSO-treated wells and dose–response curves were fitted using nonlinear regression.

### CRBN overexpression

Lentiviral production was carried out in Lenti-X 293T cells (Takara Biosciences) with cotransfection with pMD2.G and psPAX2 (Addgene, 12259 and 12260) and a lentiviral expression plasmid with PEI. Lentivirus-containing supernatants were collected 48 h and 72 h after transfection, filtered through a 0.45-µm filter and concentrated 20-fold with Lenti-X concentrator (Takara Biosciences). Cell lines were spinfected at 500*g* for 1 h at room temperature with 8 µg ml^−1^ polybrene (EMD Millipore). Lentivirus-compatible plasmids with varying CRBN expression promoters (EF-1a, hPGK and UbC) were transduced into HiBiT–ENL MOLM-13 cells. CRBN expressed plasmids by EF-1a and hPGK were selected with puromycin at 2 µg ml^−1^. CRBN expressed plasmid by UbC was selected with hygromycin at 250 µg ml^−1^. After selection, WT CRBN, EF-1a CRBN, hPGK CRBN and UbC CRBN were screened in a ten-point dose–response series against *rac*-dHTC1, (*S*)-dHTC1, (*R*)-dHTC1 and SR-1114 (three biological replicates repeated with two independent experiments). HiBiT–ENL was read out after 24 h and HiBiT counts were normalized to DMSO-treated cells.

### Immunoblotting

For adherent cell lines, cells were plated in six-well or 12-well plates at approximately 1 × 10^6^ cells per well and allowed to adhere overnight. For suspension lines, cells were plated at 1 × 10^6^ cells per well and used shortly thereafter. Wells were treated with the indicated compounds for the times indicated in each experiment. Cells were then washed with ice-cold PBS and lysed with 1× RIPA buffer supplemented with protease inhibitor and phosphatase inhibitor (Life Technologies). Lysates were sonicated and centrifuged and the supernatants were collected. Protein concentration in cell lysates was measured using a BCA assay. Then, 25 µg of denatured total protein from each sample was separated by SDS–PAGE using 4–12% Bis–Tris gels and transferred to nitrocellulose membranes. Membranes were blocked with 5% milk in Tris-buffered saline with Tween-20 (TBST) and then incubated with primary antibodies overnight at 4 °C (all antibodies were 1:1,000 in 5% milk in TBST, while GAPDH primary antibody was diluted 1:5,000 in 5% milk in TBST). Bound primary antibodies were incubated in the appropriate secondary antibody (IRDye680 goat anti-mouse: 925-68070, IRDye800 goat anti-rabbit: 926-32211; diluted 1:5000 in 5% milk in TBST). Secondary antibodies were incubated at room temperature for 90 min before washing with additional TBST and were visualized on an Odyssey CLx (LI-COR Biosciences).

Alternatively, MV4;11 CRBN^−/−^ ENL–TagBFP–P2A–mCherry cells rescued with CRBN WT or mutant were seeded in 12-well plates at 1 × 10^6^ cells per ml and treated with DMSO, 1 μM dHTC1 or 1 μM SR-1114 for 6 h. Cells were washed twice in ice-cold PBS and lysed for 15 min on ice with RIPA buffer (50 mM Tris-HCl pH 8.0, 150 mM NaCl, 1% Triton X-100, 0.5% sodium deoxycholate and 0.1% SDS) supplemented with Benzonase (25 U per ml) and 1× Halt protease inhibitor cocktail. Following clearance by centrifugation, protein concentration of lysates was determined using a Pierce BCA protein assay (23225, Fisher Scientific) and 30 μg of lysate was prepared using 4× LDS sample buffer (Thermo Fisher) and 10% 2-mercaptoethanol and run on NuPAGE 4–15% Bis–Tris gels (Thermo Fisher). Proteins were transferred to nitrocellulose membranes, blocked for 30 min in 5% milk in TBST at room temperature before incubating with primary antibodies for 1 h at room temperature. The following primary antibodies were used: ENL (1:1,000; D9M4B, Cell Signaling Technology) CRBN (1:2,000; gift from R. Eichner and F. Bassermann) and GAPDH (1:2,000; FL-335, Santa Cruz Biotechnology). Membranes were then washed in TBST and incubated with horseradish peroxidase (HRP)-conjugated secondary antibodies for 45 min at room temperature before further washes and developing with chemiluminescence films. The secondary antibody used was HRP-conjugated AffiniPure goat anti-rabbit IgG (1:10,000, Jackson ImmunoResearch, 111-035-003).

For western blots of xenografted MV4;11 cells, mouse cell depletion was performed on whole bone marrow cells using the mouse cell depletion kit and LS columns (Miltenyi Biotec). RIPA lysis was performed with purified human cells and 25 µg of protein was loaded on 10% Bis–Tris NuPAGE gels with MOPS SDS running buffer (Life Technologies). Samples were transferred on nitrocellulose iBlot 3 transfer stacks (Life Technologies) using the iBlot 3 gel transfer device (Thermo Fisher). Membranes were blocked in 5% milk in TBST for 1 h at room temperature and incubated overnight at 4 °C with antibodies to ENL (Millipore; 1:1,000 in 5% milk in TBST) or GAPDH (Cell Signaling Technologies; 1:1,000 in 5% milk in TBST). Membranes were washed, followed by incubation with the secondary IRDye 800CW donkey anti-rabbit antibody (Thermo Fisher Scientific; 1:7,000 in 5% milk in TBST) for 1 h at room temperature, and subsequent washes were performed. Membranes were imaged on a LI-COR imager.

Primary antibodies used were as follows:

ENL: D9M4B (1:1,000; Cell Signaling Technologies) or ABE2596-100UG (1:1,000; Millipore)

AF9: E5Z7U (1:1,000; Cell Signaling Technologies)

CRBN: D8H3S (1:2,000; Cell Signaling Technologies)

BRD4: E2A7X-13440 (1:1,000; Cell Signaling Technology) and Ab128874 (1:1,000; Abcam)

BRD3: Ab50818 (1:1,000; Abcam)

FBXO3: sc-514625 (1:1,000; Santa Cruz Biotechnology)

GAPDH: sc-32233 (1:1,000 or 1:5,000; Santa Cruz Biotechnology) or FL-335 (1:2,000; Santa Cruz Biotechnology)

Secondary antibodies used were as follows:

Goat anti-mouse IgG with IRDye 680RD: 925-68070 (1:5,000; LI-COR)

Goat anti-rabbit IgG with IRDye 800CW: 926-32211 (1:5,000; LI-COR)

Goat anti-rabbit IgG with HRP: 111-035-003 (1:10,000; Jackson ImmunoResearch)

Goat anti-mouse IgG with HRP: 115-035-003 (1:10,000; Jackson ImmunoResearch)

Donkey anti-rabbit with IRDye 800CW: 926-32213 (1:7,000; LI-COR)

### His_6_–CRBN^midi^–AviTag synthesis and purification

The double-stranded sequence for CRBN^midi^ was amplified from a previously published plasmid (Addgene, 215330)^41^. The sequence was cloned into a modified expression vector backbone derived from pET21a (Novagen) with an N-terminal 6×His tag and a C-terminal AviTag. The ensuing plasmid was cotransformed in BL21(DE3) cells (New England Biolabs) with biotin ligase expression vector pBirAcm (Avidity). Cultures were grown at 37 °C in Luria–Bertani growth medium to an optical density at 600 nm (OD_600_) of 0.6. Protein expression was induced at 16 °C with the addition of 100 µM biotin and IPTG to a final concentration of 1 mM. The culture was harvested by centrifugation following a 16-h incubation and resuspended in lysis buffer (50 mM Tris-HCl pH 8.0, 300 mM NaCl, 10% glycerol, 10 mM imidazole and 1 mM DTT containing cOmplete EDTA-free protease inhibitor (Roche)). Cells were lysed by two passes through a MicroFluidizer at 10,000 psi. The lysate was clarified by centrifugation at 6,000*g* for 30 min at 4 °C. The supernatant was passed over Ni-NTA agarose (Qiagen) and the protein was eluted in elution buffer (50 mM Tris-HCl pH 8.0, 300 mM NaCl, 10% glycerol, 250 mM imidazole and 1 mM DTT). Elution fractions were buffer-exchanged into 20 mM Tris-HCl at pH 8.0 and 50 mM NaCl using PD10 desalting columns (GE Life Sciences). The protein was further purified by size-exclusion chromatography (SEC) through a HiLoad Superdex column (GE) on an AKTA Pure (GE).

### SPR

SPR experiments were performed on a Biacore 8K (Cytiva). A series S streptavidin sensor chip was used for ligand immobilization (Cytiva). Biotinylated ENL_YEATS and biotinylated CRBN^midi^ were prepared in HBS-EP+ (10 mM HEPES, 150 mM NaCl, 3 mM EDTA and 0.05% P20, pH 7.4) or PBS-P+ buffer (Cytiva). Ligand immobilization was carried out in flow cell 2 with a series of ligand injections at a flow rate of 5 µl min^−1^ until the desired density was achieved (700 RU for ENL_YEATS ternary complex, 1,800 RU for ENL_YEATS small-molecule binding and 4,430 RU for CRBN^midi^). Flow cell 1 was left blank as a reference. HBS-EP+ buffer with 1% DMSO was used as running buffer and for the preparation of all analyte samples. For samples with CRBN–DDB1 in the eluent, CRBN–DDB1 was serially diluted in either HBS-EP+ and 1% DMSO buffer or HBS-EP+ and 1% DMSO buffer with 1 µM compound. Analytes were serially diluted in polystyrene 96-well plates immediately before injection. Before analyte injection, three startup cycles were run with injections of running buffer over all flow cells. Binding data were collected by injecting the analyte in a single-cycle analysis at a flow rate of 30 µl min^−1^ over two flow cells (reference and immobilized ligand) at a temperature of 25 °C. The association to biotin–ENL_YEATS or CRBN^midi^ was measured over 120 s and the dissociation was measured over 1,200 s. Duplicate injections were carried out for each sample, including the blank. Affinity curves, association constants, dissociation constants and sensorgrams were determined using Biacore 8K evaluation software. Curves were plotted in GraphPad (one-site-specific binding) and the data shown are from two or three independent repeats.

### CRBN–DDB1 FP assay

Probe displacement from the CRBN–DDB1 complex (performed by Reaction Biology) in the presence or absence of ENL (YEATS) was assessed through FP detection. First, a 9 µM (final 3 µM) solution of ENL (YEATS) was prepared in fresh assay buffer (50 mM HEPES pH 7.5, 100 mM NaCl, 0.5 mM DTT and 0.01% Tween-20). Then, 5 µl of ENL (YEATS) solution or buffer only (for no-ENL controls) was added to assay wells. Compounds were then added to assay wells using an acoustic liquid handler (Echo, Beckman) and incubated for 20 min at 4 °C. Next, a 150 nM (final 50 nM) solution of CRBN–DDB1, prepared in assay buffer, was added (5 µl) to each reaction well, followed by another 20-min incubation at room temperature. Assay buffer was added to blank wells. Lastly, 30 nM solution of BODIPY–thalidomide probe (final concentration: 10 nM; Tenova Pharma, T52674) was prepared in assay buffer and added (5 µl) to assay wells.

After a 90-min incubation at room temperature, FP signal was measured using a PheraStar plate reader (BMG Labtech) with an FP module (excitation: 485 nm, emission: 520 nm (S/P)). The observed signal was blank-corrected and converted to percentage binding relative to DMSO control. IC_50_ determinations were performed using GraphPad Prism 4.0 software with a sigmoidal dose–response (variable slope) equation or log(inhibitor) versus response (three parameters) curve.

### Expression proteomics

The MS proteomics data were deposited to the ProteomeXchange Consortium through the PRIDE^117^ partner repository with the dataset identifier PXD055068.

#### Sample preparation

Treated cells were resuspended in 200 µl of cold DPBS containing protease inhibitor (cOmplete ULTRA tablets mini EDTA-free; Roche, 05892791001; one tablet was dissolved in 10 ml of DPBS before use). Cells were lysed by probe sonication using a Branson Sonifer 250 ultrasonic cell disruptor (2 × 12 pulses, 10% power output). The protein content of whole-cell lysates was determined using the DC protein assay (Bio-Rad, 5000113 and 5000114) with absorbance at 750 nm measured on a CLARIOstar plate reader. A volume corresponding to 200 µg of protein was transferred to a new low-bind Eppendorf tube containing 48 mg of urea and brought to a total volume of 100 µl. Samples were reduced with DTT by the addition of 5 µl of a 200 mM solution (final concentration: 10 mM) and were incubated at 65 °C for 15 min. Samples were next alkylated with iodoacetamide by the addition of 5 µl of a 400 mM solution (20 mM final concentration) and incubated at 37 °C for 30 min in the dark. Proteins in each sample were precipitated by the addition of cold CH_3_OH (500 µL), CHCl_3_ (100 µL) and H_2_O (400 µL). Samples were vortexed and centrifuged (16,000*g*, 10 min, 4 °C). The supernatant was aspirated, leaving behind the protein disc, and an additional 1 ml of cold CH_3_OH was added. The samples were vortexed again and centrifuged (16,000*g*, 10 min, 4 °C). The supernatant was aspirated and the protein pellet was briefly allowed to air dry. The protein pellet was then resuspended in 160 µl of EPPS buffer (200 mM, pH 8.0) by probe sonication (ten pulses, 10% power output). Proteomes were digested with LysC by the addition of 4 µl of a 0.5 µg µl^−1^ LysC solution in high-performance liquid chromatography (HPLC)-grade water and the samples were incubated for 2 h at 37 °C. Proteomes were then digested with trypsin by the addition of 8 µl of a 0.5 µg µl^−1^ trypsin solution in trypsin resuspension buffer with 20 mM CaCl_2_ and the samples were incubated at 37 °C overnight. Peptide concentrations were determined using a Micro BCA assay (Thermo Scientific, 23235) and a volume that corresponded to 25 µg was transferred to a new low-bind Eppendorf tube and brought to a total volume of 25 µl with EPPS buffer. To each sample, 9 µl of HPLC-grade acetonitrile was added followed by 3 µl (20 µg µl^−1^ in dry acetonitrile) of the respective 6plex tandem mass tag (TMT) and the reaction was vortexed and incubated at room temperature for 1 h with additional vortexing every 20 min. The reaction was quenched by the addition of 6 µl of 5% hydroxylamine in H_2_O, vortexed and incubated at room temperature for 15 min. Each sample was acidified by the addition of 2.5 µl of LC–MS-grade formic acid. Samples were combined by taking 25 µl of each sample (~12.5 µg of labeled peptide per channel) and vacuum-centrifuged. The combined samples were desalted with a Sep-Pak C18 cartridge and offline-fractionated by HPLC.

#### Sample desalting and offline fractionation

Samples were resuspended in 500 µl of buffer A (95% H_2_O, 5% acetonitrile and 0.1% formic acid), vortexed and sonicated in a water bath for 5 min at room temperature. Before loading the sample onto a Sep-Pak C18 cartridge (Waters, WAT054955), the column was conditioned with acetonitrile (three times, 1 ml each) and equilibrated with buffer A (three times, 1 ml each). The sample was then loaded onto the column and the flowthrough of the sample was added onto the column again. The sample was desalted by adding buffer A (three times, 1 ml each) and passing it through and the sample was then eluted by the addition of 1 ml of buffer B (80% acetonitrile, 20% H_2_O and 0.1% formic acid). The eluted sample was collected into a new low-bind Eppendorf tube and was evaporated to dryness using a SpeedVac vacuum concentrator.

The sample was then resuspended in 500 µl of buffer A and fractionated using an Agilent HPLC (Agilent Infinity 1260 II system) into a 96-deep-well plate containing 20 µl of 20% formic acid as previously reported^118,119^. The peptides were eluted onto a capillary column (ZORBAX Extend-C18, 80 Å, 4.6 × 250 mm, 5 µM) and separated at a flow rate of 0.5 ml min^−1^ using the following gradient with buffer A (10 mM aqueous NH_4_HCO_3_) and buffer B (100% acetonitrile): 100% buffer A 0–2 min, 0–13% buffer B 2–3 min, 13–42% buffer B 3–60 min, 42–100% buffer B 60–61 min, 100% buffer B 61–65 min, 100–0% buffer B 65–66 min, 100% buffer A 66–75 min, 0–13% buffer B 75–78 min, 13–80% buffer B 78–80 min, 80% buffer B 80–85 min, 100% buffer A 86–91 min, 0–13% B from 91–94 min, 13–80% buffer B 94–96 min, 80% buffer B 96–101 min and 80–0% buffer B 101–102 min. The eluent collected in the 96-deep-well plate was evaporated to dryness using a SpeedVac vacuum concentrator. The peptides were resuspended in 80% acetonitrile with 0.1% formic acid buffer and combined into 12 fractions (fractions were combined down a column of the plate) by the addition 200 µl of buffer, resuspension by pipetting and combination into the next row. This process was repeated with another 200 µl. Each of the fractions had a final volume of 400 µl and was evaporated to dryness using a SpeedVac vacuum concentrator.

#### TMT LC–MS analysis

Dried fractions were solubilized in 50 ml of 0.1% trifluoroacetic acid and desalted using 2-μg-capacity ZipTips (Millipore) according to the manufacturer’s instructions. Peptides were then online eluted into a Fusion Tribrid MS instrument (Thermo Scientific) from an EASY PepMap RSLC C18 column (2 μm, 100 Å, 75 μm × 50 cm; Thermo Scientific), using a hold of 6% solvent B (80:20 acetonitrile and water, 0.1% formic acid) for 15 min, followed by a gradient of 6–28% solvent B in 105 min, then 28–49% solvent B in 15 min, 40–100% solvent B in 10 min, a 10-min hold of 100% solvent B, a return to 5% solvent B in 3 min and finally a 3-min hold of 5% solvent B. The gradient was then extended for the purpose of cleaning the column by increasing solvent B to 100% in 3 min, a 100% solvent B hold for 10 min, a return to 5% solvent B in 3 min, a 5% solvent B hold for 3 min, an increase in solvent B again to 100% in 3 min, then a 100% solvent B hold for 10 min, a return to 5% solvent B in 3 min, a 5% solvent B hold for 3 min, one last increase to 100% solvent B in 3 min and a hold of 100% solvent B for 10 min. All flow rates were 250 nl min^−1^ delivered using an nEasy-LC1000 nanoLC system (Thermo Scientific). Solvent A consisted of water and 0.1% formic acid. Ions were created at 2.0 or 1.9 kV using an EASY Spray source (Thermo Scientific) held at 50 °C. A synchronous precursor selection MS3 method was selected on the basis of the work by Ting et al.^120^. Scans were conducted between 380 and 2,000 *m*/*z* at a resolution of 120,000 for MS1 in the Orbitrap mass analyzer at a standard automatic gain control (AGC) target (as defined in Thermo Scientific Xcalibur version 4.6.67.17) and a maximum injection of 50 ms. Collision-induced dissociation (CID) was then performed in the linear ion trap of peptide monoisotopic ions with a charge of 2–8 above an intensity threshold of 5 × 10^3^, using a quadrupole isolation of 0.7 *m*/*z* and a CID energy of 35%. The ion trap AGC target was also set to standard with a maximum injection time of 50 ms. Dynamic exclusion duration was set at 60 s and ions were excluded after one iteration within the ±10-ppm mass tolerance window. The top ten MS2 ions in the ion trap between 400 and 1,200 *m*/*z* were then chosen for higher-energy C-trap dissociation at 65% energy. Detection occurred in the Orbitrap at a resolution of 50,000 and an AGC target of 1 × 10^5^ (or 200% custom setting as defined in Thermo Scientific Xcalibur version 4.6.67.17), with an injection time of 120 ms (MS3). All scan events occurred within a 3-s specified cycle time. The analysis was performed at The Herbert Wertheim UF Scripps Institute for Biomedical Innovation and Technology, MS and Proteomics Core Facility (RRID:SCR_023576).

#### Peptide and protein identification and quantification

The raw spectrum files were uploaded to the Integrated Proteomics Pipeline (IP2) (http://ip2.scripps.edu/ip2/mainMenu.html) and MS2 and MS3 files were extracted from the raw files using RAW converter and searched using the ProLuCID algorithm against a reverse-concatenated and nonredundant variant of the human UniProt database (2016-07). The precursor ion mass tolerance for a minimum envelop of three isotopic peaks was set to 50 ppm. Cysteine residues were searched with a static modification for carbamidomethylation (+57.02146 Da) and up to two differential modifications were allowed per peptide for methionine oxidation (+15.994915 Da). Lysine and N-terminal residues were also searched with a static modification corresponding to the TMT (+229.1629 Da). Peptides were required to be a minimum of 6 aa long. ProLuCID data were filtered using DTAselect 2.0 within IP2 to allow for a false-positive rate less than 1% at the spectrum level. MS3-based peptide quantification was performed with a reporter ion mass tolerance set to 20 ppm with IP2. Proteins were required to have at least two unique peptides. Peptide-spectrum matches were grouped on the basis of protein identifier and peptides were excluded if (1) the sum of the reporter ion intensities was less than 10,000; (2) the coefficient of variance was greater than 0.5, or (3) they were nonunique or nontryptic peptide sequences. Proteins were quantified by summing the reporter ion intensities across all matching peptide-spectrum matches and normalizing to the highest signal channel per protein. Plots were generated with the log_2_ fold change of the quotient of DMSO-treated cells with drug-treated cells and the inverse log_10_ of a two-tailed Student’s *t*-test.

### Determination of ENL target genes

Typical and asymmetrical targeted genes were previously determined by ChIP-seq^26^. dTAG-responsive genes were also previously defined by RNA sequencing (RNA-seq) following dTAG-13-mediated degradation of ENL^54^. These are categorized separately in Fig. 4e, whereas the combined list is used in Fig. 1h.

### In vivo experiments

#### Xenograft studies

These studies were approved by the Dana-Farber Cancer Institute’s Institutional Animal Care and Use Committee (IACUC) and were carried out with compliance to all ethical regulations. The mice were housed in cages within the Optimize rack system at a temperature of 72 ± 2 °F, a relative humidity of 35–55% and a photoperiod of 12 h of light and 12 h of dark. Studies were conducted in 6–8-week-old female NOD.Cg-*Prkdc*^*scid*^ *Il2rg*^*tm1Wjl*^/SzJ mice under IACUC protocol number 16-021 at the Dana-Farber Cancer Institute. A total of 1 × 10^6^ MV4;11 cells were intravenously injected per mouse and engraftment was confirmed in the peripheral blood by flow-cytometry-based detection of human CD45^+^ cells using an LSR Fortessa, BD Biosciences (human CD45–PE, clone HI30, 1:200; mouse CD45–APC–Cy7, clone 30-F11, 1:100; BioLegend). Upon confirmation of engraftment, mice were randomly assigned to treatment groups and received three doses of (*R*)-dHTC1, (*S*)-dHTC1 (50 mg kg^−1^ intraperitoneally every 12 h) or vehicle. The compounds were dissolved in 5% DMAC and 95% PEG-300 (40%) + NaCl (0.9%) (60%).

Mice were killed 1 h after the last dosing using CO_2_ inhalation followed by cervical dislocation. Bone marrow cells were isolated from tibias, femurs, iliac crest and spine by crushing the bones with a mortar and pestle in PBS (Life Technologies) + 2% FBS (Life Technologies) and passing the cell suspension through a 40-µm cell strainer (Falcon). Spleens were smashed using a syringe plunger and passed through a 40-µm cell strainer (Falcon). Blood was collected by cardiac puncture and red blood cell (RBC) lysis was performed using RBC lysis buffer (BioLegend). The differentiation status of bone marrow, spleen and blood cells was assessed by flow cytometry (LSR Fortessa, BD Biosciences) using the following antibodies: human CD45–PE, clone HI30, 1:200; mouse CD45–APC–Cy7, clone 30-F11, 1:100; human CD11b-FITC, clone ICRF44, 1:100 (BioLegend).

#### Pharmacokinetic studies

Mice were housed in the DMPK animal facility in Building A of Shanghai ChemPartner.

The room environment was well controlled: ventilating at least 15 times per hour, light 12 h per day, temperature of approximately 20–24 °C and humidity of approximately 40–70%. The study rooms were disinfected and cleaned before the start of the study and the operation area was cleaned after each dosing or sampling during the study conduct. Animals had access to certified rodent diet (SCXK [SH] 2017-0012, Shanghai Pu Lu Teng Biological Technology) ad libitum. Water was available ad libitum. The nutritional composition and levels of contaminants of the diet and impurities and contaminants of the water were monitored. The health status of the animals was evaluated in accordance with accepted animal husbandry procedures and deemed suitable for experimental use.

Pharmacokinetic analyses were performed by Shanghai ChemPartner in male C57BL/6 mice (6–8 weeks old, 19–21 g; *n* = 27 mice) obtained from Shanghai Jihui Laboratory Animal (qualification no. SCXK (SH) 2022-0009, 20220009012123). Animals were housed with free access to food and water.

Compounds were administered intravenously (2 mg kg^−1^ or 5 ml kg^−1^, tail vein), orally (2 mg kg^−1^ or 10 ml kg^−1^, gavage) or intraperitoneally (10 mg kg^−1^ or 10 ml kg^−1^, injection). At defined time points, blood was collected under manual restraint either by facial-vein sampling or by terminal cardiac puncture. Approximately 95 μl of blood was drawn into K_2_EDTA tubes, kept on ice and centrifuged promptly (2,000*g*, 5 min, 4 °C) to isolate plasma within 15 min.

Alternatively, male C57BL/6 mice (6–8 weeks old, 19–21 g; *n* = 9 mice) obtained from Shanghai Jihui Laboratory Animal (qualification no. SCXK (SH) 2022-0009, 20220009020523) were administered compounds intraperitoneally (25, 50 or 75 mg kg^−1^, all 10 ml kg^−1^, injection). At defined time points, mice were anesthetized with isoflurane and approximately 26 µl of blood per time point was collected into K_2_EDTA tubes through facial or cardiac puncture for terminal bleeding. Tubes were kept on ice and centrifuged promptly (2,000*g*, 5 min, 4 °C) to isolate plasma within 15 min.

Plasma samples were processed by protein precipitation. For undiluted plasma, 20 μl of sample was used. Alternatively, samples were prepared as tenfold dilutions (2 µl of sample diluted into 18 µl of blank plasma) or 20-fold dilutions (2 µl of sample diluted into 38 µl of blank plasma, shaken for 5 min, with 20 µl aliquoted for analysis). These samples were mixed with 200 μl of methanol containing 40 ng ml^−1^ diclofenac or propranolol as the internal standard (IS). The mixture was vortexed for 5 min and centrifuged (3,800*g*, 10 min). Then, 90 µl of supernatant was transferred to a fresh plate and 3 μl was injected for LC–MS/MS analysis.

Concentrations were calculated using a calibration curve using 0.2–1,000 ng ml^−1^ of the analyte in male C57 plasma.

#### PPB assay

PPB assays were performed by Shanghai ChemPartner. Plasma protein binding was determined using a 96-well equilibrium dialysis format with a sodium phosphate buffer system (0.05 M, pH 7.4). The buffer was prepared by combining monobasic and dibasic sodium phosphate solutions with sodium chloride, followed by dilution and pH adjustment to 7.4.

Stock solutions of test and reference compounds were diluted to generate two working solutions:

Solution A (0.5 mM): 10 µl of a 10 mM DMSO stock diluted with 190 µl of DMSO

Solution B (0.02 mM): 8 µl of solution A added to 192 µl of phosphate buffer (final DMSO 4%)

Working solutions were spiked into plasma by adding 20 µl of solution B to 380 µl of blank plasma in each well of a 96-well plate (final compound concentration: 1 µM, 0.2% DMSO). Dialysis chambers were preloaded with 100 µl of blank buffer on the receiver side. An equal volume of spiked plasma was added to the donor side. Chambers were sealed with a plastic lid and incubated on a shaker (60 rpm) for 5 h at 37 °C.

For *T*_0_ samples, immediately before incubation, 25-µl aliquots of spiked plasma were mixed 1:1 (v/v) with buffer and quenched with 200 µl of acetonitrile containing IS (warfarin or quinidine). For postdialysis samples (5 h), aliquots (25 µl) from both plasma (donor) and buffer (receiver) chambers were transferred to new plates, mixed 1:1 with the opposite matrix and quenched with 200 µl of acetonitrile containing IS. All samples were shaken at 600 rpm for 10 min and centrifuged at 5,594*g* for 15 min. Supernatants (50 µL) were transferred to new plates containing 50 µl of purified water and stored at −20 °C until LC–MS/MS quantification.

The fraction unbound was determined from the ratio of compound concentration in the buffer chamber to that in the plasma chamber at equilibrium, corrected for recovery using *T*_0_ samples.

### UPS screen

#### Plasmids and oligonucleotides

The design and construction of the human UPS-focused sgRNA library used for ENL stability screens and the human CRBN DMS library were described previously^36,49^. The engineering of the fluorescent protein stability reporter of ENL for the FACS-based CRISPR–Cas9 and DMS screens and the CRBN point mutants for the validation of the DMS screen were performed as described previously^34,49^. The fluorescent protein stability reporter of GSPT1 was generated by inserting the GSPT1 coding sequence into pArtichoke plasmid (Addgene, 73320) by Gibson assembly. All the BRD4-based stability reporters were generated previously^34^. For splitHalo assays, two separate Gateway-compatible pRRL vectors were generated using cpHalo∆ sequence from Addgene 205703 (pRRL-SFFV-gw-EGFP-(GSG)9-cpHalo∆, where ‘gw’ is the Gateway cassette) and the published Hpep6 sequence^68^ (pRRL-SFFV-Hpep6-GGGS-gw-EF1as-TagBFP), into which FBXO3 and BRD4 short (BRD4s) coding sequences were inserted by Gateway cloning.

#### Cell culture

KBM7 iCas9 cells were a gift from J. Zuber. Lenti-X 293T lentiviral packaging cells (Clontech), HEK293T cells and RPE1 cells were cultured in DMEM (Gibco) supplemented with 10% fetal calf serum (FCS; Thermo Fisher) and 1% penicillin–streptomycin (Thermo Fisher). MV4;11 cells were cultured in RPMI supplemented with 1% FCS and 1% penicillin–streptomycin. KBM7 cells were cultured in IMDM supplemented with 1% FCS and 1% penicillin–streptomycin. All cells were grown in a humidified incubator at 37 °C and 5% CO_2_ and routinely tested for *Mycoplasma* contamination.

For lentiviral production, Lenti-X 293T cells were seeded in six-well plates or 10-cm dishes and transfected at approximately 80% confluency with 0.6/3.4 μg of target plasmid, 1.7/0.3 μg of psPAX2 (Addgene, 12260) and 0.85/0.15 μg of pMD2.G (Addgene, 12259) using PEI (PolySciences). Virus supernatant was collected after 60 h, filtered using a 0.45-μm filter and stored in aliquots for transduction.

#### FACS-based CRISPR–Cas9 ENL and BRD4s stability screens

The UPS-focused library packaged lentivirus-containing supernatant was used to transduce KBM7 ENL–TagBFP–P2A–mCherry or BRD4s–TagBFP–P2A–mCherry cells harboring iCas9 at a multiplicity of infection (MOI) of 0.19 and 1,000-fold library representation for ENL and an MOI of 0.03 and 300-fold library representation for BRD4s using 8 μg ml^−1^ polybrene (Szabo Scandic, SACSC-134220). Library-transduced KBM7 cells were selected with 1 mg ml^−1^ G418 (Gibco) for 14 days and expanded; Cas9 expression was induced with doxycycline at 0.4 μg ml^−1^ (PanReac AppliChem). Then, 3 days after doxycycline induction, 50 million cells per condition were treated with DMSO, *rac*-dHTC1 (10 μM) or SR-1114 (10 μM) for 8 h in two biological replicates for ENL or with DMSO, dHTC2 (2 μM) or dHTC3 (2 μM) for 16 h in two biological replicates for BRD4s. Cells were washed with PBS, stained with APC anti-mouse Thy1.1 antibody (1:400; 202526, BioLegend) and Zombie near-infrared (NIR) fixable viability dye (1:1,000; BioLegend) in the presence of Human TruStain FcX Fc receptor blocking solution (1:400; 422302, BioLegend) for 10 min at 4 °C in the dark and fixed with 1 ml of BD CytoFix fixation buffer (BD Biosciences, 554655) for 30 min at 4 °C in the dark. Cells were washed with and stored in FACS buffer (PBS with 5% FCS and 1 mM EDTA) at 4 °C overnight.

The next day, cells were strained through a 35-μm nylon mesh and sorted on a BD FACSAria Fusion (BD Biosciences) operated on BD FACSDiva software (version 8.0.2) using a 70-μm nozzle excluding aggregate, dead (Zombie NIR-positive), Cas9-negative (GFP-negative) and sgRNA library-negative (Thy1.1–APC-negative) cells. The remaining cells were sorted on the basis of their ENL–BFP or BRD4s–BFP and mCherry levels into ENL/BRD4s^hi^ (5–10% of cells), ENL/BRD4s^mid^ (25–50% of cells) and ENL/BRD4s^low^ (5–10% of cells) fractions. Cells corresponding to at least 750-fold library representation were sorted per each sample replicate.

DNA libraries of sorted fractions for next-generation sequencing (NGS) were prepared as described previously^121^. In short, genomic DNA was extracted by cell lysis (10 mM Tris-HCl, 10 mM EDTA, 150 mM NaCl and 0.1% SDS), proteinase K treatment (New England Biolabs) and DNAse-free RNAse digest (Thermo Fisher), followed by two rounds of phenol extraction and isopropanol precipitation.

Barcoded NGS libraries for each sorted fraction were prepared using a two-step PCR protocol using AmpliTaq Gold polymerase (Invitrogen). Barcodes for each sample were introduced in a first PCR, the product of which were purified using Mag-Bind TotalPure NGS beads (Omega Bio-tek) and amplified in a second PCR introducing the standard Illumina adaptors. The final Illumina libraries were bead-purified, pooled and sequenced on NovaSeq 6000 instrument (Illumina) following a 100-bp, paired-end recipe.

Screen analysis was performed as previously described^121^. In short, sequencing reads were trimmed using fastx-toolkit (version 0.0.14), aligned using Bowtie 2 (version 2.4.2) and quantified using featureCounts (version 2.0.1). The crispr-process-nf Nextflow workflow is available from GitHub (https://github.com/ZuberLab/crispr-process-nf/tree/566f6d46bbcc2a3f49f51bbc96b9820f408ec4a3). For statistical analysis, we used the crispr-mageck-nf Nextflow workflow, also available from GitHub (https://github.com/ZuberLab/crispr-mageck-nf/tree/c75a90f670698bfa78bfd8be786d6e5d6d4fc455). To calculate gene-level enrichment, the sorted populations (ENL/BRD4s^high^ or ENL/BRD4s^low^) were compared to the ENL/BRD4s^mid^ populations in MAGeCK (version 0.5.9)^114^ using median-normalized read counts.

#### FACS-based DMS screens

A total of 18 million MV4;11 CRBN^−/−^ ENL–TagBFP–P2A–mCherry cells were transduced with the CRBN DMS library virus supernatant at an MOI of 0.17, yielding a calculated library representation of 1,760 cells per variant. For the transduction, 1 million cells were seeded in a 24-well plate with 8 μg ml^−1^ polybrene (Szabo Scandic, SACSC-134220) and filled to 1 ml with the titrated amount of virus and culture medium. The plate was centrifuged at 760*g* for 45 min at 37 °C and then cells were pooled and expanded. Next, 3 days after transduction, library-transduced cells were selected with 1 μg ml^−1^ Blasticidin (Gibco, R21001) for 7 days and selected cells were expanded. A total of 50 million cells per condition were treated with *rac*-dHTC1 (1 μM) or SR-1114 (1 μM) for 8 h in three biological replicates. Cells were washed with PBS, stained with Zombie NIR fixable viability dye (1:1,000; BioLegend) for 10 min at 4 °C in the dark and fixed with 1 ml of BD CytoFix fixation buffer (BD Biosciences, 554655) for 30 min at 4 °C in the dark. Cells were washed with and stored in FACS buffer (PBS with 5% FCS and 1 mM EDTA) at 4 °C overnight. Two sets of 50 million unsorted cells were also harvested as a control, directly washed and frozen.

The next day, cells were strained through a 35-μm nylon mesh and sorted on a BD FACSAria Fusion (BD Biosciences) operated on BD FACSDiva software (version 8.0.2) using a 70-μm nozzle excluding aggregate, dead (Zombie NIR-positive) and reporter-negative (BFP-negative and mCherry-negative) cells. The remaining cells were sorted on the basis of their ENL^−1^BFP and mCherry levels into an ENL^hi^ fraction (30^−1^35% of cells). Cells corresponding to at least 3,000-fold library representation were sorted per each sample replicate.

DNA libraries of sorted and unsorted cell pools for NGS were prepared as described previously^49,121^. In short, genomic DNA was extracted by cell lysis (10 mM Tris-HCl, 10 mM EDTA, 150 mM NaCl and 0.1 % SDS), proteinase K treatment (New England Biolabs) and DNAse-free RNAse digest (Thermo Fisher), followed by two rounds of phenol extraction and isopropanol precipitation.

CRBN variant complementary DNA was amplified by PCR from genomic DNA using AmpliTaq Gold polymerase (Invitrogen) and primers CRBN_GA_fwd (AGGTGTCGTGACGTACGGGATCCCAGGACCATGGCCGGCGAAGGAG) and CRBN_GA_rev (GGGGGGGGGGCGGAATTAATTCCTACTACTTACAAGCAAAGTATTACTTTGTCTGGAC). The cycle number for specific amplification of the 1.4-kb CRBN target was confirmed by agarose gel electrophoresis. PCR reactions for each sample were pooled and purified using Mag-Bind TotalPure NGS beads (Omega Bio-tek). The library preparation from the amplified DNA was performed using the Tagment DNA TDE1 enzyme and IDT for Illumina unique dual indices (Illumina). Library concentrations were quantified with the Qubit 2.0 fluorometric quantitation system (Life Technologies) and the size distribution was assessed using the 2100 Bioanalyzer instrument (Agilent). For sequencing, samples were diluted and pooled into NGS libraries and sequenced on NovaSeq 6000 instrument (Illumina) following a 100-bp, paired-end recipe.

Raw sequencing reads were converted to fastq format with SAMtools (version 1.15.1) and BEDTools (version 2.30.0) using the bamtofastq function. Sequencing reads were trimmed using Trim Galore (version 0.6.6) using nextera and pair modes. Short reads were aligned to the CRBN cassette and SAM files were generated using mem algorithm from the bwa software package (version 0.7.17). The SAM file was converted to BAM using SAMtools and mutation calling was performed using the AnalyzeSaturationMutagenesis tool from GATK (version 4.1.8.1). Given our sequencing strategy, >98% of reads corresponded to WT sequences and were filtered out during this step. Next, relative frequencies of variants were calculated for each position and variants that were covered by <1 in 30,000 reads were excluded from further analysis. Read counts for each variant were then normalized to total read count of each sample and log_2_ fold changes of ENL^hi^ over unsorted pools were calculated. To correct for differential drug potency, each variant was then normalized to the maximum log_2_ fold change. For drug comparisons, log_2_ fold changes of ENL^hi^ over unsorted pools of each drug were subtracted. Heat maps were generated using the pheatmap (version 1.0.12) package in R (version 4.1.0) or with GraphPad Prism.

#### Flow cytometry ENL reporter assay

MV4;11 CRBN^−/−^ ENL–TagBFP–P2A–mCherry or GSPT1–GFP–P2A–mCherry cells were transduced with lentivirus expressing WT or mutant CRBN in pRRL-EF1a-CRBN-IRES-BlastR plasmid to generate stable cell lines. For evaluation of reporter degradation on the different CRBN backgrounds, cells were treated with DMSO or *rac*-dHTC1, SR-1114 (both 1 μM, 6 h, ENL reporter) or CC-885 (1 μM, 5 h, GSPT1 reporter) before flow cytometry analysis on an LSR Fortessa (BD Biosciences) operated on BD FACSDiva software (version 9.0).

#### Flow cytometry BRD4 reporter assay

KBM7 iCas9 expressing the reporter SFFV–BRD4(s)–mTagBFP–P2A–mCherry or its different truncations were generated previously^34^. To quantify the influence of genetic perturbation on compound-induced BRD4(s) reporter degradation, the stable cell line was transduced with lentiviral sgRNA (pLentiV2-U6-sgRNA-IT-PGK-iRFP720-P2A-Neo) to 30–50% transduction efficiency. Cas9 expression was induced by treating with doxycycline (0.4 μg ml^−1^) for 3 days, followed by a 16-h treatment with DMSO, dBET6 (10 nM), IBG3 (0.1 nM), dHTC2 (2 μM) or dHTC3 (2 μM). Cells were stained for sgRNA expression with an APC-conjugated anti-mouse Thy1.1 antibody (202526, BioLegend; 1:400) and human TruStain FcX receptor blocking solution (422302, BioLegend; 1:1,000) for 5 min at 4 °C in FACS buffer (PBS with 5% FBS and 1 mM EDTA). Cells were washed and resuspended in FACS buffer and analyzed on an LSR Fortessa (BD Biosciences) operated on BD FACSDiva software (version 9.0).

Flow cytometry data analysis was performed in FlowJo version 10.10.0. mCherry and BFP median fluorescence intensity values were normalized by background subtraction of the respective values from reporter-negative KBM7 cells. BRD4 abundance was calculated as the ration of background-subtracted BFP to mCherry median fluorescence intensity and is displayed normalized to DMSO-treated cells.

#### Flow cytometry FBXO3 and BRD4s splitHalo assay

RPE1 cells stably expressing SFFV–FBXO3–EGFP–cpHalo and SFFV–Hpep6–BRD4s–EF1as–TagBFP were seeded in 96-well plates. After attachment, cells were pretreated with carfilzomib (1 μM) and, when indicated, with DMSO, JQ1, GSK778 (iBET-BD1) or GSK046 (iBET-BD2) (all at 20 μM) for 1 h and then treated with DMSO, dBET6, *epi*-dHTC3 or dHTC3 at the concentrations indicated for each experiment in combination with 100 nM TAMRA-CA for 3 h. After treatment, cells were washed, trypsinized and resuspended in FACS buffer (PBS with 5% FBS and 1 mM EDTA) and analyzed on an LSR Fortessa (BD Biosciences) with plate reader operated on BD FACSDiva software (version 9.0).

Flow cytometry data analysis was performed in FlowJo version 10.10.0. PE and GFP median fluorescence intensity values from GFP^+^BFP^+^ cells were normalized by background subtraction of the respective values from reporter-negative cells. SplitHalo signal was calculated as the ratio of background-subtracted PE to GFP median fluorescence intensity and is displayed normalized to DMSO-treated cells.

sgAAVS1: GCTGTGCCCCGATGCACAC

sgDCAF16: GTTCCAGTTTGGGGACACAA

sgFBXO3-1: GTTCATACCGAATTCACAA

sgFBXO3-2: GAATGTGTAGCAACAACTG

sgTRIM25-1: GATGACTGCAAACAGAAAGG

sgTRIM25-2: GAACACGGTGCTGTGCAACG

### Chemogenomic CRISPR–Cas9 KO screen in NALM6 cells

The Genome-wide pooled CRISPR/Cas9 KO screen was performed by the ChemoGenix platform (IRIC, Université de Montréal; https://chemogenix.iric.ca/) as previously described^122^. Briefly, a NALM6 clone bearing an integrated iCas9 expression cassette generated by lentiviruses made from pCW-Cas9 (Addgene, 50661) was transduced with the genome-wide KO EKO sgRNA library (278,754 different sgRNAs)^122^. After thawing the library from liquid N_2_ and letting it recover in 10% FBS RPMI for 1 day, KOs were induced for 7 days of culture with 2 µg ml^−1^ doxycycline. The pooled library was then split in different T-75 flasks (28 × 10^6^ cells per flask; a representation of 100 cells per sgRNA) in 70 ml at 4 × 10^5^ cells per ml. Cells were treated with dHTC2 (150 nM) or dHTC3 (400 nM), using 1,000× DMSO stock solution for 8 days with monitoring of growth every 2 days, diluting back to 4 × 10^5^ cells per ml and adding more compound to maintain same final concentration whenever cells reached 8 × 10^5^ cells per ml. Over that period, treated cells had 4.64 (dHTC2) or 1.39 (dHTC3) population doublings whereas DMSO-only treated negative controls had a 7.5 population doubling. Cells were collected, genomic DNA was extracted using the Gentra Puregene kit according to the manufacturer’s instructions (Qiagen) and sgRNA sequences were PCR-amplified as described^122^. sgRNA frequencies were obtained by NGS (Illumina NextSeq500). Reads were aligned using Bowtie 2.2.5 in the forward direction only (--norc option) with otherwise default parameters and total read counts per sgRNA were tabulated. Context-dependent chemogenomic interaction scores were calculated using a modified version of the RANKS algorithm^122^, which uses guides targeting similarly essential genes as controls to distinguish condition-specific chemogenomic interactions from nonspecific fitness and essentiality phenotypes. Raw read counts are available upon request from the ChemoGenix platform.

### Crystallization of CRBN^midi^ bound to dHTC1

Structural coordinates were deposited to the PDB and are available under accession number 9GY3.

His_6_–CRBN^midi^ protein (Addgene, 215330) was expressed and purified as previously described^41^. CRBN^midi^ at 3.4 mg ml^−1^ in a buffer containing 20 mM HEPES pH 7.5, 500 mM NaCl and 0.5 mM TCEP was mixed with a 4 M excess of dHTC1 (364 µM, 1.8% (v/v) DMSO final). The complex was subjected to cocrystallization using the sitting-drop vapor diffusion method across several sparse matrix screens at 20 °C by mixing equal volumes of protein solution and reservoir solution. Crystal hits from Morpheus H10 (0.1 M buffer system 3, pH 8.5, 0.12 M alcohol and 30% precipitant mix 2) (Molecular Dimensions) were isolated directly from the drop and flash-cooled in liquid nitrogen.

Diffraction data were collected at Diamond Light Source beamline I24 using the Eiger CdTe 9M detector at a wavelength of 0.6199 Å. The 2.2-Å dataset was processed using Dials^123^, indexed in the *P*3_1_ space group and the structure was solved by molecular replacement in phenix.phaser^124^ using the coordinates of PDB 8RQA as a search model. Phenix.refine^125^ and Coot^126^ were used for refinement and model building.

### RNA-seq

The data discussed in this publication were deposited to the National Center for Biotechnology Information (NCBI) Gene Expression Omnibus (GEO)^127^ under accession number GSE278582.

Total RNA was extracted from mouse-cell-depleted bone marrow cells from MV4;11 xenografts using the RNeasy Kit (Qiagen). RNA samples were run on a TapeStation (Agilent) to assure an RNA integrity score > 8 and quantified by Qbit (Thermo Fisher). The NEBNext poly(A) mRNA magnetic isolation module and NEBNext Ultra II RNA library prep kit (NEB BioLabs) were used to prepare RNA libraries. Sequencing was performed using the NextSeq500 (Illumina) as 37-bp paired-end sequencing with 20 million reads. Further analysis was performed on expressed genes (rlog expression < 3).

### Cryo-EM

#### Cloning, protein expression and purification

N-terminal His_6_-tagged hsDDB1^ΔBPB^ (UniProt Q16531, amino acids 396–705 replaced with a GNGNSG linker) and FLAG-tagged hsCRBN (UniProt Q96SW2) were cloned into pAC8-derived vectors^128^. The N-terminal GST-tagged eGFP–hsCRBN was cloned into a pLIB vector, which was a gift from J.-M. Peters (Addgene, 80610)^129^. The pAC8 constructs were recombinantly expressed using the baculovirus expression system (Invitrogen). To generate baculovirus from the pAC8-derived constructs, 1 μg of plasmids were cotransfected with linearized baculoviral DNA in *Spodoptera* *frugiperda* Sf9 cells grown in ESF 921 medium (Expression Systems) at a density of 1.0 × 10^6^ cells per ml. To generate baculovirus from the pLIB construct, 0.5 µg of the pLIB construct was transformed in DH10EMBacY cells^130^ and recombinant bacmid was isolated using the Invitrogen PureLink quick plasmid miniprep kit (Invitrogen, K210010). Sf9 cells were transfected at a density of of 1.0 × 10^6^ cells per ml and viral titer was increased by three rounds of amplification. For protein expression, *Trichoplusia* *ni* High Five cells, grown in SF-4 Baculo Express ICM (BioConcept) to a density of 2 × 10^6^ cells per ml, were coinfected with 1.5% (v/v) each of DDB1^ΔBPB^ -expressing and CRBN-expressing baculoviruses. After 48 h of incubation, cells were collected (20 min, 3,300*g*).

For His_6_–hsDDB1^ΔBPB^/FLAG–hsCRBN-infected cells, the pellet was resuspended and sonicated in FLAG lysis buffer (50 mM HEPES–NaOH pH 7.4, 200 mM NaCl, 0.1% (v/v) Triton X-100 and 1 mM TCEP) supplemented with protease inhibitors. The cell lysate was treated with Benzonase for 10 min and cleared by ultracentrifugation (60 min, 120,000*g*), the supernatant was incubated with FLAG antibody-coated beads (Genscript, L00432) and washed with wash buffer (50 mM HEPES–NaOH pH 8.0 and 200 mM NaCl). Bound proteins were eluted with 0.15 mg ml^−1^ 1× FLAG (DYKDDDK) peptide and diluted to 50 mM NaCl with anion-exchange chromatography (IEX) buffer A (50 mM HEPES–NaOH pH 7.4 and 1 mM TCEP). Samples were added to Poros 50 HQ resin (Thermo Scientific) and eluted with a linear salt gradient from 50 mM to 1,000 mM. Peak fractions from IEX were concentrated using centrifugal concentrators (Amicon, 30-kDa molecular weight cutoff (MWCO)) and polished by SEC (HiLoad 16/600 Supderdex 200 pg, Cytiva) in CRBN SEC buffer (50 mM HEPES–NaOH pH 7.4, 150 mM NaCl and 1 mM TCEP). Peak fractions were concentrated by centrifugation, flash-frozen in liquid nitrogen and stored at −80 °C.

For His_6_–hsDDB1^ΔBPB^/GST–eGFP–hsCRBN cells, the pellet was resuspended and sonicated in GST lysis buffer (50 mM Tris pH 8.0, 200 mM NaCl and 2 mM TCEP) supplemented with protease inhibitors. The cell lysate was cleared by ultracentrifugation and treated with Benzonase for 10 min, incubated with glutathione Sepharose 4B (Cytiva, GE17-0756-01) and washed with GST lysis buffer. Elution fractions including the target protein were pooled and concentrated using 30-kDa centrifugal concentrators and cleaved with tobacco etch virus protease at 4 °C overnight. The sample was diluted to 50 mM NaCl and cleared through IEX, followed by SEC using eGFP–CRBN SEC buffer (25 mM HEPES–NaOH pH 7.4, 200 mM NaCl and 2 mM TCEP).

The C-terminal His_6_-tagged ENL YEATS domain (amino acids 1–148; UniProt Q03111) and C-terminal Strep-Avi-tagged ENL YEATS domain were cloned into a pNIC-Bio2 (SGC Oxford) vector and expressed in a LOBSTR *Escherichia* *coli* expression strain (Kerafast, EC1002). After transformation, 2-L cultures in Terrific Broth medium were grown at 37 °C to an OD_600_ of ~0.6 and induced with 0.35 mM IPTG. Temperature was decreased to 18 °C, proteins were expressed overnight and cultures were harvested by centrifugation (20 min, 3,300*g*). Cell pellets were resuspended in His lysis buffer (50 mM HEPES–NaOH pH 7.4, 200 mM NaCl, 20 mM imidazole, 5% (v/v) glycerol and 1 mM TCEP) or Strep lysis buffer (50 mM HEPES–NaOH pH 7.4, 200 mM NaCl, 5% (v/v) glycerol and 2 mM TCEP) supplemented with protease inhibitors and lysed using sonication. After clearance by ultracentrifugation (60 min, 120,000*g*), the supernatant was treated with Benzonase for 10 min and incubated with high-affinity Ni-charged resin (Genscript, L00223) or Strep-Tactin XT 4Flow high-capacity resin (IBA Life Sciences, 2-5030-002). Bound proteins were eluted with increasing imidazole concentrations (150–750 mM) or 50 mM biotin. His_6_-tagged protein elution fractions were concentrated using a centrifugal concentrator (10-kDa MWCO) and polished by SEC (Superdex 75 Increase 10/300 GL, Cytiva) in ENL SEC buffer (30 mM HEPES–NaOH pH 7.4, 300 mM NaCl and 3 mM TCEP). Peak fractions were concentrated by centrifugation, flash-frozen in liquid nitrogen and stored at −80 °C. Strep-tagged protein elution fractions were concentrated using a centrifugal concentrator (10-kDa MWCO) and biotinylated overnight in 4 °C with BirA biotin ligase. Biotinylated ENL YEATS domain was further polished by SEC using ENL SEC buffer.

#### EM sample preparation and data collection

DDB1^∆BPB^•CRBN, dHTC1 and ENL YEATS were mixed and incubated on ice for 1 h at a final concentration of 1.4, 14 and 2.8 µM (dataset 1) and 1.6, 16 and 3.2 µM (dataset 2), respectively, keeping DMSO concentrations below 2% (v/v). Grids (Quantifoil UltrAuFoil R 0.6/1) were glow-discharged (20 mA, 120 s, 39 Pa) and vitrified in a Leica EM-GP operated at 10 °C and 90% relative humidity. Grids were preincubated with 4 µl of 10 µM CRBN agnostic IKZF1-Q146A;G151N (amino acids 140–196) for 1 min and then blotted from behind for 4 s with NCI Micro Whatman no. 1 filter paper. Immediately, 4 µl of the sample were applied to the grids before blotting for 4 s and plunging into liquid ethane, kept at −181 °C. Datasets were collected using Smart EPU (version 3.7) on a Thermo Scientific Titan Krios equipped with a Thermo Fisher Scientific Selectris energy filter (10-eV slit width) and Thermo Fisher Scientific Falcon 4 direct electron detector. Videos (49 frames per video, 2.87-s exposure time) were acquired at 300 kV at a nominal magnification of ×165,000 in counting mode with a pixel size of 0.736 Å per pixel. One video was recorded per hole with aberration-free image shift (as implemented in EPU). A total of 9,856 videos for dataset 1 and 13,027 videos for dataset 2 were recorded with defocus varying from −0.8 to −2.0 µm and a total exposure rate of 50.22 e^−^ per A^2^.

#### Data processing and model building

cryoSPARC (version 4.5.3)^131^ was used for all processing steps. All resolutions given are based on the Fourier shell correlation (FSC) = 0.143 threshold criterion^132^. For dataset 1, 9,856 videos were corrected for beam-induced motion and contrast transfer function (CTF) was estimated on the fly in cryoSPARC live. Low-quality videos were rejected and 1,267,417 particles were extracted (1.31 Å per pixel) from the 6,558 remaining micrographs after TOPAZ (version 0.2.5a)^133^ particle picking. The particles were cleaned up in three rounds of heterogeneous refinement. Preferred orientation became apparent at this step. Thus, the remaining 994,856 particles were further classified using heterogeneous refinement, which revealed a small number of classes (95,520 particles) representing a more balanced range of views. The selected 95,520 particles were reextracted at 0.92 Å per pixel. For dataset 2, 13,027 videos were corrected from beam-induced motion and CTF was estimated on the fly in cryoSPARC live. Low-quality videos were rejected and the remaining 11,541 micrographs were split into two groups, resulting in 5,173,911 particles extracted (1.31 Å per pixel) after TOPAZ (version 0.2.5a)^133^ particle picking. The particles were cleaned up in two rounds of heterogeneous refinement. Similarly to dataset 1, the resulting 1,536,327 particles were further classified using heterogeneous refinement, which again revealed a small number of classes (89,792 particles) with more balanced views. The selected 89,792 particles were reextracted at 0.92 Å per pixel and combined with the particles from dataset 1. The pooled particles were used in a final homogeneous refinement, yielding a consensus reconstruction at global resolution of 2.6 Å. Local refinement with a soft mask encompassing CRBN and ENL YEATS yielded a final reconstruction at 2.9 Å (EMD-47174), which was followed by postprocessing using deepEMhancer to improve the density for ENL YEATS were used for model building. CRBN (PDB 8TNQ) and ENL YEATS (PDB 6HPY) were fit into the density in UCSF ChimeraX (version 1.6.1)^134,135^ and relaxed using ISOLDE (version 1.6.0)^136^ and Rosetta (version 3.13)^137^. The relaxed model was manually adjusted in Coot (version 0.9.8.0)^126^ and prepared for refinement with phenix.ready_set (version 1.19.2-4158)^138^, followed by refinement using reference restraints in phenix.real_space_refine (version 1.19.2-4158)^139^ against the unsharpened maps. For figures, DDB1^ΔBPB^ (from PDB 8TNQ) was placed on the basis of a superposition of CRBN. The final model (9DUR) and map (EMD-47174) were deposited to the PDB and EM Data Bank, respectively. Data collection parameters and refinement statistics are available in Supplementary Table 9. To compare the model with other reported CRBN structures, every reported closed conformation CRBN structure in complex with a molecular glue degrader or a PROTAC was superimposed (PDB 4TZ4, 5FQD, 5HXB, 5V3O, 6BN7, 6BN8, 6BN9, 6BOY, 6H0G, 6UML, 6XK9, 7LPS, 8D7U, 8D7V, 8D7W, 8D7Z, 8D80, 8D81, 8DEY, 8G66, 8OIZ, 8OJH, 8TNP, 8TNQ, 8TNR, 8TZX, 8U15, 8U16 and 8U17) with the C-terminal domain (amino acids 318–442) of the CRBN in the model^13–15,17,53,74,78,140–146^. For comparison with a non-ligand-bound ENL structure, we superimposed PDB 6HQ0 with the ENL YEATS domain in the model^115^. Backbone r.m.s.d. values were calculated in UCSF ChimeraX (version 1.9)^134,135^. Structural biology applications used in this project were compiled and configured by SBGrid^147^.

#### Interface analysis

Interface comparison was made with Rosetta (version 3.13) by reordering the jump number of fold tree such that the CRBN and ENL YEATS interface was compared and then the following command was used to obtain buried interface solvent-accessible surface area and shape complementarity score^52^: sasa_and_sc_calc_scripts.sh.

### Immunoprecipitation and sample preparation for immunoprecipitation–MS

#### Experimental setup

A total of 1 × 10^7^ cells per immunoprecipitation were collected and lysed in lysis buffer (50 mM Tris pH 8, 200 mM NaCl, 2 mM TCEP, 0.1% NP-40, 10 U of turbonuclease per 200 µl of buffer and 1× cOmplete protease inhibitor tablet per 5 ml of buffer) and sonicated on ice for five rounds of 2 s followed by 10-s pauses at 25% amplitude. After centrifugation clarification, the following steps were performed on an opentrons OT2 liquid handler. Cell lysate, 10 µg of FLAG–CRBN–DDB1ΔB, 1 µM of MLN4924 and CSN5i-3 (neddylation trap)^148^ and 1 µM of selected degraders or DMSO vehicle control were transferred to PCR plate and incubated for 1 h at 4 °C. Then, 3 µl of prewashed and resuspended anti-FLAG magnetic bead 25% slurry (Pierce) were added to the lysates, followed by a second incubation for 1 h at 4 °C. Beads were washed three times with wash buffer (50 mM Tris pH 8, 2 mM TCEP, 0.1% NP-40 and 1× cOmplete protease inhibitor tablet per 5 ml of buffer) containing the required compounds, followed by three additional nondetergent (50 mM Tris pH 8, 2 mM TCEP and 1× cOmplete protease inhibitor tablet per 5 ml of buffer) washes containing the required compounds. After the final wash step, samples were eluted using 0.1 M glycine-HCl pH 2.7. Tris (1 M, pH 8.5) was added to elution to reach a pH of 8. Samples were then reduced with 10 mM TCEP for 30 min at room temperature, followed by alkylation with 15 mM iodoacetamide for 45 min at room temperature in the dark. Alkylation was quenched by the addition of 10 mM DTT. The resuspended protein samples were digested with 2 μg of LysC and 1 μg of trypsin overnight at 37 °C. Sample digests were acidified with formic acid to a pH of 2–3 before desalting using C18 solid-phase extraction plates (SOLA, Thermo Fisher Scientific). Desalted peptides were dried in a vacuum-centrifuged and reconstituted in 0.1% formic acid for LC–MS analysis.

#### Label-free quantitative MS with data-independent acquisition parallel accumulation–serial fragmentation and data analysis

Data were collected using a TimsTOF Ultra2 (Bruker Daltonics) coupled to a nanoElute2 LC pump (Bruker Daltonics) through a CaptiveSpray nanoelectrospray source. Peptides were separated on a reversed-phase C18 column (25 cm × 75 μm inner diameter, 1.6 μm; IonOpticks) containing an integrated CaptiveSpray emitter. Peptides were separated using a 30-min gradient of 2–30% buffer B (acetonitrile in 0.1% formic acid) with a flow rate of 250 nl min^−1^ and column temperature maintained at 50 °C.

The thermal ionization MS elution voltages were calibrated linearly with three points (Agilent ESI-L tuning mix ions; 622, 922 and 1,222 *m*/*z*) to determine the reduced ion mobility coefficients (1/*K*_0_). To perform data-independent acquisition parallel accumulation–serial fragmentation (diaPASEF), we used py_diAID^149^, a Python package, to assess the precursor distribution in the *m*/*z* ion mobility plane to generate a diaPASEF acquisition scheme with variable window isolation widths aligned to the precursor density in *m*/*z*. Data were acquired using 20 cycles with three mobility window scans each (creating 60 windows) covering the diagonal scan line for doubly and triply charged precursors, with singly charged precursors able to be excluded by their position in the *m*/*z* ion mobility plane. These precursor isolation windows were defined between 350 and 1,250 *m*/*z*, with 1/*K*_0_ of 0.6–1.45 V s cm^−2^.

The diaPASEF raw file processing and control of peptide-level and protein-level false discovery rates (FDRs), assembly of proteins from peptides and protein quantification from peptides were performed using library-free analysis in DIA-NN (version 1.8)^150^ searched against a Swiss-Prot human database (January 2021). Database search criteria largely followed the default settings for directDIA including: tryptic with two missed cleavages, carbamidomethylation of cysteine and oxidation of methionine and a precursor *Q*-value (FDR) cutoff of 0.01. The precursor quantification strategy was set to robust LC (high accuracy) with retention-time-dependent cross-run normalization. Resulting data were filtered to only include proteins that had a minimum of three counts in at least four replicates of each independent comparison of treatment sample to the DMSO control. Protein abundances were globally normalized using in-house scripts in the R framework (R Development Core Team, 2014). Proteins with missing values were imputed by random selection from a Gaussian distribution either with a mean of the nonmissing values for that treatment group or with a mean equal to the median of the background (in cases where all values for a treatment group were missing^42^). Protein abundances were scaled and significant changes comparing the relative protein abundance of these treatments to DMSO control comparisons were assessed by moderated *t*-test as implemented in the limma package within the R framework^151^.

### ENL mutagenesis

Mutations were induced with primers on a lentiviral compatible construct expressing ENL–BFP.

R16A: 5′-gagctggggcatgccgcccaactgc-3′ and 5′-cttgcgcagttgggcggcatgccccagc-3′.

D31A: 5′- gttcactcacgcctggatggtgtttg-3′ and 5′- caccatccaggcgtgagtgaaccc-3′.

M33A: 5′- ctcacgactgggccgtgtttgtcc-3′ and 5′- caaacacggcccagtcgtgagtgaa-3′.

Q41A: 5′- cgcggccccgaggcctgtgacatccagc-3′ and 5′- atgtcacaggcctcggggccgcggacaaaca-3′.

K72A: 5′- cccccctacgccgtagaggagtcggggtacg-3′ and 5′- ccccgactcctctacggcgtaggggggct-3′.

E74A: 5′- ccctacaaagtagccgagtcggggtacgctgg-3′ and 5′- gtaccccgactcggctactttgtagggggg-3′.

R96A: 5′- ggaggagccggccaaggtctgcttcacc-3′ and 5′-gaagcagaccttggccggctcctccttgtt-3′.

Halfway primers: 5′-cgcccttcccaacagttgcgcagcctgaatgg-3′ and 5′-ctgttgggaagggcgatcggtgcgggcctcttc-3′.

In a 1:1 ratio, each mutated plasmid was Gibson-assembled into the other half of the pRRL-SFFV-ENL-GGGS-3×V5-BFP backbone. Lentivirus of each plasmid (pRRL-SFFV-ENL-GGGS-3×V5-BFP, pRRL-SFFV-ENL^R16A^-GGGS-3×V5-BFP, pRRL-SFFV-ENL^D31A^-GGGS-3×V5-BFP, pRRL-SFFV-ENL^M33A^-GGGS-3×V5-BFP, pRRL-SFFV-ENL^Q41A^-GGGS-3×V5-BFP, pRRL-SFFV-ENL^K72A^-GGGS-3×V5-BFP, pRRL-SFFV-ENL^E74A^-GGGS-3×V5-BFP and pRRL-SFFV-ENL^R96A^-GGGS-3×V5-BFP) was prepared as described above and transduced into MV4;11 cells. Cells were selected for BFP expression. BFP signal was measured by flow cytometry after a 24-h treatment with (*S*)-dHTC1, (*R*)-dHTC1 or SR-1114 and normalized to DMSO (*n* = 3) at the doses indicated.

### Reporting summary

Further information on research design is available in the Nature Portfolio Reporting Summary linked to this article.

## Online content

Any methods, additional references, Nature Portfolio reporting summaries, source data, extended data, supplementary information, acknowledgements, peer review information; details of author contributions and competing interests; and statements of data and code availability are available at 10.1038/s41589-025-02137-2.

## Supplementary information

Supplementary Information Supplementary Methods and Fig. 1, NMRs, flow cytometry gating strategies and raw sensorgrams.

Reporting Summary

Supplementary Table 1 Key physicochemical properties for the library of amine building blocks used in high-throughput derivatization.

Supplementary Table 2 Crude reaction products were screened for ENL degradation (5 µM, 24 h) in MV4;11 cells expressing HiBiT–ENL. HiBiT was luminescence normalized to DMSO:PBS vehicle-control-treated cells (single independent experiment).

Supplementary Table 3 Resynthesized hits were rescreened by HiBiT–ENL and counterscreened against BRD4–HiBiT (three biological replicates). HiBiT luminescence was normalized to DMSO:PBS vehicle-control-treated cells.

Supplementary Table 4 dHTC1 proteomics. Quantitative TMT-based expression proteomics analysis of MV4;11 cells treated with *rac*-dHTC1 (1 µM, 16 h) or DMSO vehicle control (5,513 proteins with >2 peptides; data were filtered using DTAselect 2.0 within IP2). *P* values were calculated using a two-tailed Student’s *t*-test (three biological replicates).

Supplementary Table 5 dHTC1 AP–MS results. AP–MS results showing relative protein abundance by label-free proteomics following enrichment of recombinant FLAG–CRBN–DDB1ΔB from MOLT4 cell lysates treated with 1 µM dHTC1 or DMSO. *P* values were calculated using a moderated two-sided *t*-test implemented in the limma package (four independent replicates of each treatment group).

Supplementary Table 6 CRBN FP signal (background subtracted and normalized to DMSO) following treatment with the indicated compounds, in the presence of ENL (3 µM) or buffer control (mean; three biological replicates).

Supplementary Table 7 MV4;11 *CRBN*-KO cells expressing ENL–TagBFP–P2A–mCherry were transduced with a DMS library, which reintroduced CRBN with single-amino-acid substitutions within a 10-Å radius of the thalidomide-binding site. After these cells were treated with SR-1114 (1 µM, 8 h), endpoint ENL levels were quantified by flow cytometry. Results are depicted indicating log_2_ fold change of ENL^high^ over the unsorted pools.

Supplementary Table 8 MV4;11 *CRBN*-KO cells expressing ENL–TagBFP–P2A–mCherry were transduced with a DMS library, which reintroduced CRBN with single-amino-acid substitutions within a 10-Å radius of the thalidomide-binding site. After these cells were treated with dHTC1 (1 µM, 8 h), endpoint ENL levels were quantified by flow cytometry. Results are depicted indicating log_2_ fold change of ENL^high^ over the unsorted pools.

Supplementary Table 9 dHTC1 CRBN^midi^ crystal statistics.

Supplementary Table 10 Cryo-EM statistics.

Supplementary Table 11 Crude reaction products were screened for BRD4 degradation (2 µM, 16 h) in MV4;11 cells expressing BRD4–HiBiT. HiBiT luminescence was normalized to DMSO:PBS vehicle-control-treated cells (single independent experiment).

Supplementary Table 12 Hits from Fig. 5b were rescreened at 2 or 10 µM (16 h) in MV4;11 BRD4–HiBiT cells and counter screened against MV4;11 HiBiT–ENL cells. HiBiT luminescence was normalized to DMSO:PBS vehicle-control-treated cells (three biological replicates).

Supplementary Table 13 dHTC2 and dHTC3 proteomics. Quantitative TMT-based expression proteomics analysis of MV4;11 cells treated with dHTC2 and dHTC3 (1 µM, 16 h) or DMSO vehicle control (5,399 proteins with >2 peptides depicted; data were filtered using DTAselect 2.0 within IP2). *P* values were calculated using a two-tailed Student’s *t*-test (three biological replicates).

## Source data

Source Data Fig. 1 Statistical source data.

Source Data Fig. 1 Unprocessed western blots.

Source Data Fig. 2 Statistical source data.

Source Data Fig. 2 Unprocessed western blots.

Source Data Fig. 3 Statistical source data.

Source Data Fig. 4 Statistical source data.

Source Data Fig. 4 Unprocessed western blots.

Source Data Fig. 5 Statistical source data.

Source Data Extended Data Fig. 1 Statistical source data.

Source Data Extended Data Fig. 1 Unprocessed western blots.

Source Data Extended Data Fig. 2 Statistical source data.

Source Data Extended Data Fig. 2 Unprocessed western blots.

Source Data Extended Data Fig. 3 Statistical source data.

Source Data Extended Data Fig. 4 Statistical source data.

Source Data Extended Data Fig. 5 Statistical source data.

Source Data Extended Data Fig. 5 Unprocessed western blots

Source Data Extended Data Fig. 6 Statistical source data.

Source Data Extended Data Fig. 8 Statistical source data.

Source Data Extended Data Fig. 9 Statistical source data.

Source Data Extended Data Fig. 10 Statistical source data.

Source Data Extended Data Fig. 10 Unprocessed western blots.

## Data Availability

The sequencing data discussed in this publication were to deposited the NCBI GEO^127^ under accession number GSE278582. The MS proteomics data were deposited to the ProteomeXchange Consortium through the PRIDE^117^ partner repository with the dataset identifier PXD055068. Structural coordinates were deposited to the PDB and are available under accession numbers 9GY3 (CRBN^midi^:dHTC1) and 9DUR (DDB1^∆BPB^•CRBN–dHTC1–ENL YEATS). The cryo-EM data generated by this study were deposited to the EM Data Bank under accession code EMD-47174. Source data are provided with this paper.
