# Supplementary material for: High-throughput ligand diversification to discover chemical inducers of proximity
Source: Nat Chem Biol. 2026 Feb 16;22(9):1540–52. doi: 10.1038/s41589-025-02137-2 (PMC13249469; doi:10.1038/s41589-025-02137-2)

# Extended Data Figure 1h

Boxes indicate regions shown in figures.

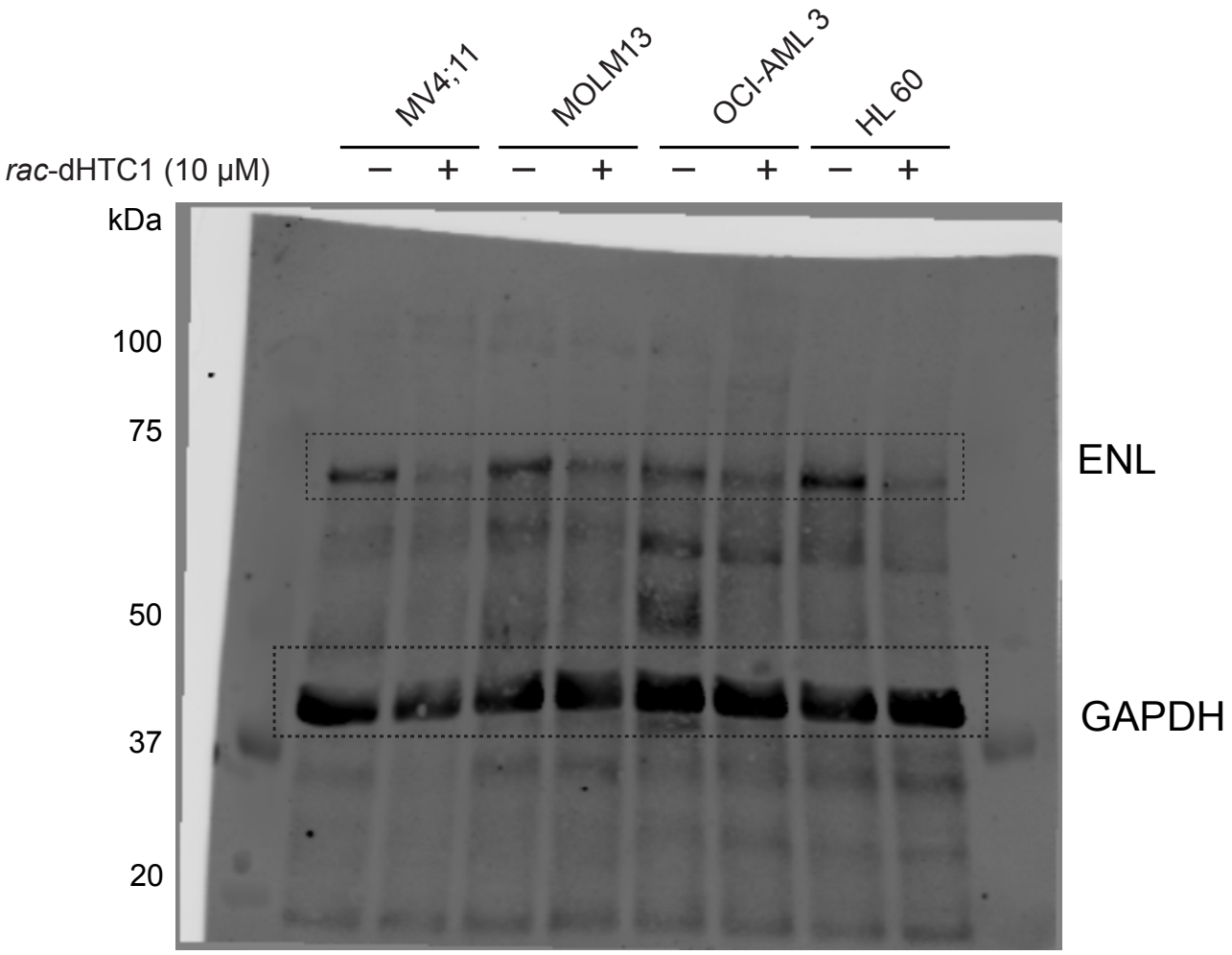


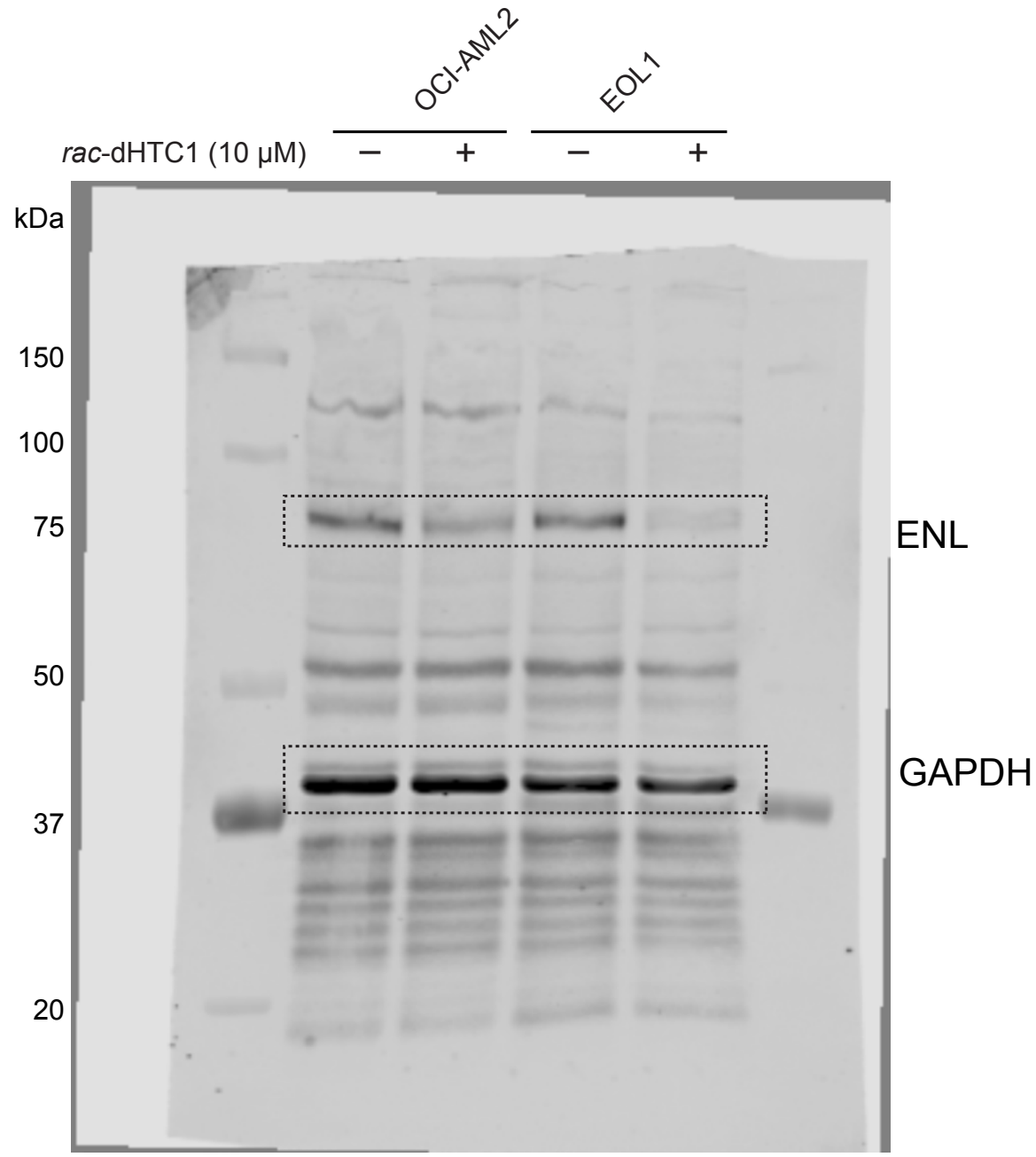


# Extended Data Figure 1j

Boxes indicate regions shown in figures.

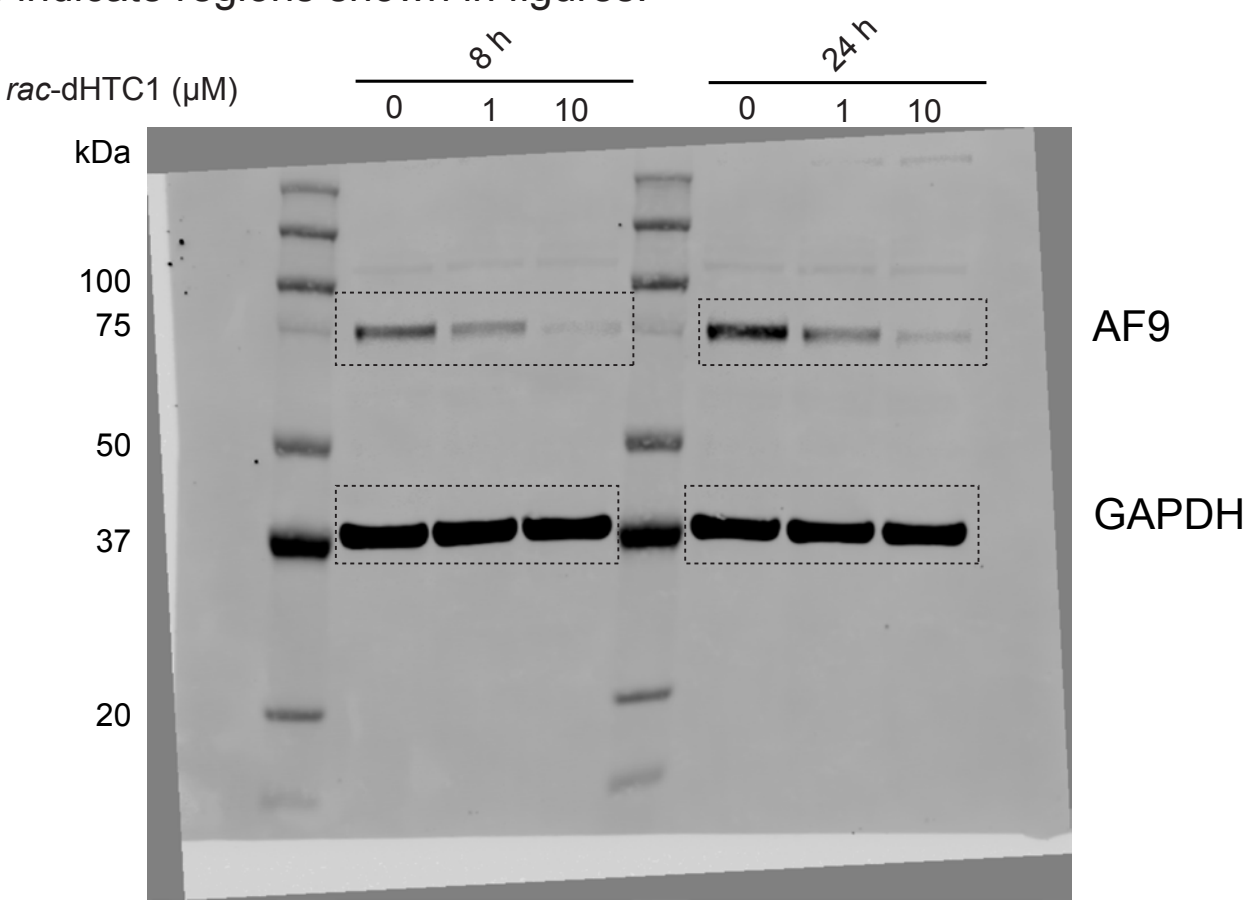

Supplement: Supplementary file 5 [file 41589_2025_2137_MOESM25_ESM.pdf]
