# Supplementary material for: High-throughput ligand diversification to discover chemical inducers of proximity
Source: Nat Chem Biol. 2026 Feb 16;22(9):1540–52. doi: 10.1038/s41589-025-02137-2 (PMC13249469; doi:10.1038/s41589-025-02137-2)
Supplement: Supplementary file 7 [file 41589_2025_2137_MOESM2_ESM.pdf]

nature portfolio

Corresponding author(s): Michael Erb

Last updated by author(s): Nov 19, 2025

Reporting Summary

Nature Portfolio wishes to improve the reproducibility of the work that we publish. This form provides structure for consistency and transparency in reporting. For further information on Nature Portfolio policies, see our Editorial Policies and the Editorial Policy Checklist.

Statistics

For all statistical analyses, confirm that the following items are present in the figure legend, table legend, main text, or Methods section.

n/a	Confirmed	
☐	☒	The exact sample size (n) for each experimental group/condition, given as a discrete number and unit of measurement
☐	☒	A statement on whether measurements were taken from distinct samples or whether the same sample was measured repeatedly
☐	☒	The statistical test(s) used AND whether they are one- or two-sided<br>*Only common tests should be described solely by name; describe more complex techniques in the Methods section.*
☒	☐	A description of all covariates tested
☐	☒	A description of any assumptions or corrections, such as tests of normality and adjustment for multiple comparisons
☐	☒	A full description of the statistical parameters including central tendency (e.g. means) or other basic estimates (e.g. regression coefficient) AND variation (e.g. standard deviation) or associated estimates of uncertainty (e.g. confidence intervals)
☐	☒	For null hypothesis testing, the test statistic (e.g. F, t, r) with confidence intervals, effect sizes, degrees of freedom and P value noted<br>*Give P values as exact values whenever suitable.*
☒	☐	For Bayesian analysis, information on the choice of priors and Markov chain Monte Carlo settings
☒	☐	For hierarchical and complex designs, identification of the appropriate level for tests and full reporting of outcomes
☒	☐	Estimates of effect sizes (e.g. Cohen's d, Pearson's r), indicating how they were calculated

Our web collection on statistics for biologists contains articles on many of the points above.

Software and code

Policy information about availability of computer code

Data collection

1) Proteomics: Thermo Scientific Xcalibur software v4.6.67.17
2) Luminescence measurements: CLARIOstar v5.4
3) Protein concentration quantification: CLARIOstar v5.4
4) Immunoblot imaging: LICORbio Image Studio version 5.2
5) HRMS: Agilent MassHunter Workstation Software vB.08.00
6) Flow cytometry: BD FACSDiva software v8.0.2 or v9.0.
7) Surface plasmon resonance: Biacore S200 evaluation software
8) Cryo-EM: Thermo Fisher Smart EPU v3.7.
9) HTRF & Fluorescence Polarization: PHERAstar software version 4.00 R4

Data analysis

1) Graphical and statistical analysis: GraphPad Prism v10 for MacOS
2) Proteomics: Integrated Proteomics Pipeline was used to process the raw proteomics data which was searched using the ProLuCID algorithm
3) CRISPR-Cas9 ENL stability screen: fastx-toolkit (v0.0.14), Bowtie2 (v2.4.2) featureCounts (v2.0.1), MAGeCK (v0.5.9). The crispr-process-nf Nextflow workflow is available at https://github.com/ZuberLab/crispr-process-nf/tree/566f6d46bbcc2a3f49f51bbc96b9820f408ec4a3. For statistical analysis used crispr-mageck-nf Nextflow workflow, available at https://github.com/ZuberLab/crispr-mageck-nf/tree/c75a90f670698bfa78bfd8be786d6e5d6d4fc455.
4) FACS based DMS screening: samtools (v1.15.1), bedtools (v2.30.0), Trim Galore (v0.6.6), bwa software package (v0.7.17), AnalyzeSaturationMutagenesis tool from GATK (v4.1.8.1).
5) CRBNmidi:dHTC1 crystal structure: DIALS (v3.21.1) (https://dials.github.io/), phenix.phaser, phenix.refine, coot, ChimeraX (1.7-1.9), pyMOL (3.0.3)
6) Surface plasmon resonance: Biacore S200 evaluation software.

7) Cryo-EM and crystallography: CryoSPARC v4.5.3, TOPAZ v0.2.5a, deepEMhancer, ISOLDE (v1.6.0), Rosetta (v3.13), COOT (v0.9.8.0), phenix.ready_set (v1.19.2-4158), phenix.real_space_refine (v1.19.2-4158), ChimeraX (1.6.1-1.9), pyMOL (3.0.3), sasa_and_sc_calc_scripts.sh
8) APMS analysis- Statistical Analysis Limma package
9) RNA sequencing analysis- DESeq2 software package
10) Pharmacokinetics analysis- WinNonlin V 8.2 (Pharsight corp. CA, USA) and Microsoft Excel (16.1)
11) Chemogenic CRISPR screen: RANKS algorithm, Bowtie 2.2.5

For manuscripts utilizing custom algorithms or software that are central to the research but not yet described in published literature, software must be made available to editors and reviewers. We strongly encourage code deposition in a community repository (e.g. GitHub). See the Nature Portfolio guidelines for submitting code & software for further information.

Data

Policy information about availability of data

All manuscripts must include a data availability statement. This statement should provide the following information, where applicable:

- Accession codes, unique identifiers, or web links for publicly available datasets
- A description of any restrictions on data availability
- For clinical datasets or third party data, please ensure that the statement adheres to our policy

Data Availability. The sequencing data discussed in this publication have been deposited in NCBI's Gene Expression Omnibus and are accessible through GEO Series accession number GSE 278582 (https://www.ncbi.nlm.nih.gov/geo/query/acc.cgi?acc=GSE 278582). The mass spectrometry proteomics data have been deposited to the ProteomeXchange Consortium via the PRIDE partner repository with the dataset identifier PXD055068. Structural coordinates are deposited in the Protein Data Bank and are available under accession numbers 9GY3 (CRBNmidi:dHTC1) and 9DUR (DDB1ΔBPB•CRBN/dHTC1/ENL YEATS). The cryo-EM data generated by this study has been deposited to the Electron Microscopy Data Base with accession code EMD-47174. All figure data is available as Source Data.

Research involving human participants, their data, or biological material

Policy information about studies with human participants or human data. See also policy information about sex, gender (identity/presentation), and sexual orientation and race, ethnicity and racism.

Reporting on sex and gender	This study did not include research conducted on human participants.
Reporting on race, ethnicity, or other socially relevant groupings	Not applicable
Population characteristics	Not applicable
Recruitment	Not applicable
Ethics oversight	Not applicable

Note that full information on the approval of the study protocol must also be provided in the manuscript.

Field-specific reporting

Please select the one below that is the best fit for your research. If you are not sure, read the appropriate sections before making your selection.

☒ Life sciences ☐ Behavioural & social sciences ☐ Ecological, evolutionary & environmental sciences

For a reference copy of the document with all sections, see nature.com/documents/nr-reporting-summary-flat.pdf

Life sciences study design

All studies must disclose on these points even when the disclosure is negative.

Sample size	No statistical methods were used for the determination of sample sizes. Sample sizes of 3 or more were used for all cell based assays according to accepted literature standards.
Data exclusions	For all proteomics data sets, proteins with fewer than three distinct peptides or those with less than six amino acids were excluded from analysis. Data exclusions and filtering for cryo-EM studies are presented in Extended Data Figure 7. Flow cytometry gating strategies are discussed below in this document and in Supplementary Figure 2.
Replication	The number of replicates is listed in each figure legend. Experiments were repeated at least once with similar results.
Randomization	All mammalian cancer cell lines were cultured identically. All wells and plates were randomly chosen for each set of treatment conditions.
Blinding	All mammalian cancer cell lines were cultured identically. Blinding was not used because the initial conditions for each treatment group were identical. No subjective measurements were done in the course of this study.

Reporting for specific materials, systems and methods

We require information from authors about some types of materials, experimental systems and methods used in many studies. Here, indicate whether each material, system or method listed is relevant to your study. If you are not sure if a list item applies to your research, read the appropriate section before selecting a response.

Materials & experimental systems

n/a	Involved in the study
☐	☒ Antibodies
☐	☒ Eukaryotic cell lines
☒	☐ Palaeontology and archaeology
☐	☒ Animals and other organisms
☒	☐ Clinical data
☒	☐ Dual use research of concern
☒	☐ Plants

Methods

n/a	Involved in the study
☒	☐ ChIP-seq
☐	☒ Flow cytometry
☒	☐ MRI-based neuroimaging

Antibodies

Antibodies used	Primary Antibodies Used:<br>ENL: D9M4B (1:1000, Cell Signaling Technologies) or ABE2596-100UG (1:1000, Millipore)<br>AF9: E5Z7U (1:1000, Cell Signaling Technologies)<br>CRBN: D8H3S (1:2000, Cell Signaling Technologies)<br>BRD4: E2A7X-13440 (1:1000 Cell Signaling Technology) and Ab128874 (1:1000, Abcam)<br>BRD3: Ab50818 (1:1000, Abcam)<br>FBXO3: sc-514625 (1:1000, Santa Cruz Biotechnology)<br>GAPDH: sc-32233 (1:1000 or 1:5000, Santa Cruz Biotechnology) or FL-335 (1:2000, Santa Cruz Biotechnology)<br><br>Secondary Antibodies Used:<br>Goat anti-Mouse IgG with IRDye 680RD: 925-68070 (1:5,000, LI-COR)<br>Goat anti-Rabbit IgG with IRDye 800CW: 926-32211 (1:5,000, LI-COR)<br>Goat anti-Rabbit IgG with HRP: 111-035-003 (1:10,000, Jackson ImmunoResearch)<br>Goat anti-Mouse IgG with HRP: 115-035-003 (1:10,000, Jackson ImmunoResearch)<br>Donkey anti-Rabbit with IRDye800CW: 926-32213 (1:7000, LI-COR)
Validation	For all primary antibodies used in this work, the manufacturers' websites validate each antibody for use in western blots with human cell lines.

Eukaryotic cell lines

Policy information about cell lines and Sex and Gender in Research

Cell line source(s)	HL-60 (gift of the laboratory of Dr. Jay Bradner), KBM7 (gift of the laboratory of T. Brummelkamp) and KBM7 iCas9 (gift of the laboratory of Dr. Johannes Zuber), were cultured in 10% FBS in IMDM. OCI-AML2 (gift of Dr. Bradner), OCI-AML3 (DSMZ catalog number ACC 582), and Kasumi-1 (ATCC catalog number CRL-2724) were cultured in 20% FBS in RPMI 1640. HB11;19 (gift of the laboratory of Prof. Akihiko Yokoyama), Jurkat (gift of the laboratory of Prof. Michael Bollong) MV4;11 and MOLM-13 (gifts of Dr. Jay Bradner) Nalm-6 (given by ChemoGenix, University of Montreal) and EOL1 (DSMZ catalog ACC 386) were cultured in 10% FBS in RPMI 1640. RPE-1 (ATCC catalog number CRL-4000), HEK-293T (ATCC catalog number CRL-3216) and Lenti-X 293T (Takara Bio catalog number 632180) were cultured in 10% FBS in DMEM. All cell lines were confirmed mycoplasma negative at 1-month intervals and never tested positive. All cell lines were cultured in media using heat inactivated FBS, supplemented with streptomycin and penicillin and stored in a 37 °C incubator at 5% CO2.
Authentication	The cell lines were used as provided without further authentication.
Mycoplasma contamination	All cell lines were tested monthly for mycoplasma contamination and always tested negative.
Commonly misidentified lines (See ICLAC register)	No commonly misidentified cell lines were used in this study.

Animals and other research organisms

Policy information about studies involving animals; ARRIVE guidelines recommended for reporting animal research, and Sex and Gender in Research

Laboratory animals	This study used C57BL/6 and Cg-Prkdcscidll2rgtm1Wjl/SzJ (NSG) mice. All mice were 6-8 weeks old at the time of the study.
Wild animals	This study did not employ wild animals.

Reporting on sex	In vivo studies were performed in mice of both genders (pharmacokinetic studies in male C57BL/6 mice and MV4;11 cells deriving from a male patient were xeno-transplanted into female NSG mice). To the best of our knowledge, there is no evidence for gender-specific differences regarding pharmacokinetics or antileukemic effects of the small molecules in this study.<br><br>Pharmacokinetic and antileukemic studies were carried out with treatment groups of ≥ 3 mice in order to establish statistical significance.
Field-collected samples	This study did not use field collected samples.
Ethics oversight	This study was approved by the Institutional Animal Care and Use Committee (IACUC) protocol number 16-021 at Dana-Farber Cancer Institute. Pharmacokinetic data was provided by Shanghai ChemPartner which used an AAALAC and OLAW accredited animal facility.

Note that full information on the approval of the study protocol must also be provided in the manuscript.

Plants

Seed stocks	No plants were used in this study.
Novel plant genotypes	Not applicable.
Authentication	Not applicable.

Flow Cytometry

Plots

Confirm that:

- ☐ The axis labels state the marker and fluorochrome used (e.g. CD4-FITC).
- ☐ The axis scales are clearly visible. Include numbers along axes only for bottom left plot of group (a 'group' is an analysis of identical markers).
- ☐ All plots are contour plots with outliers or pseudocolor plots.
- ☐ A numerical value for number of cells or percentage (with statistics) is provided.

Methodology

Sample preparation	Sample preparation is described in detail in the methods section.<br><br>To engineer our reporter, MV4;11 CRBN-/- ENL-TagBFP-P2A-mCherry or GSPT1-GFP-P2A-mCherry cells were transduced with lentivirus expressing wild-type or mutated CRBN in pRRL-EF1a-CRBN-IRES-BlastR plasmid to generate stable cell lines. For evaluation of reporter degradation on the different CRBN backgrounds, cells were treated with DMSO or dHTC1, SR-1114 (both 1µM, 6h, ENL reporter) or CC885 (1µM, 5h, GSPT1 reporter) before flow cytometry analysis on an LSR Fortessa (BD Biosciences) operated on BD FACSDiva software (v9.0).<br><br>For FACS based DMS screens, 18 million MV4;11 CRBN-/- ENL-TagBFP-P2A-mCherry cells were transduced with the CRBN DMS library virus supernatant at a multiplicity of infection of 0.17 yielding a calculated library representation of 1,760 cells per variant. For the transduction, 1 million cells were seeded in a 24-well plate with 8µg/mL polybrene (Szabo Scandic, SACSC-134220) and filled to 1mL with the titrated amount of virus and culture medium. The plate was centrifuged at 760g for 45min at 37°C and then cells were pooled and expanded. 3 days after transduction, library-transduced cells were selected with 1µg/mL Blasticidin (Gibco, R21001) for 7 days and selected cells were expanded. 50 million cells per condition were treated with dHTC1 (1µM) or SR-1114 (1µM) for 8h in three biological replicates. Cells were washed with PBS, stained with Zombie NIR Fixable Viability Dye (1:1000, BioLegend) for 10min at 4°C in the dark, and fixed with 1mL BD CytoFix Fixation Buffer (BD Biosciences, 554655) for 30min at 4°C in the dark. Cells were washed with and stored in FACS buffer (PBS with 5% FCS and 1mM EDTA) at 4°C overnight. Two sets of 50 million unsorted cells were also harvested as control, and directly washed and frozen.<br><br>For the CRISPR-Cas9 ENL stability screen, the UPS-focused library packaged lentivirus containing supernatant was used to transduce KBM7 ENL-TagBFP-P2A-mCherry cells harboring a doxycycline-inducible Cas9 allele (iCas9) at a multiplicity of infection of 0.19 and 1,000x-fold library representation using 8µg/mL polybrene (Szabo Scandic, SACSC-134220). Library-transduced KBM7 cells were selected with 1mg/mL G418 (Gibco) for 14 days, expanded and Cas9 expression was induced with doxycycline at 0.4 µg/mL (PanReac AppliChem). Three days after doxycycline induction, 50 million cells per condition were treated with DMSO, dHTC1 (10µM) or SR-1114 (10µM) for 8h in two biological replicates. Cells were washed with PBS, stained with APC anti-mouse Thy1.1 antibody (1:400, 202526, BioLegend) and Zombie NIR Fixable Viability Dye (1:1000, BioLegend) in the presence of Human TruStain FcX Fc Receptor Blocking Solution (1:400, 422302, BioLegend) for 10min at 4°

C in the dark, and fixed with 1mL BD CytoFix Fixation Buffer (BD Biosciences, 554655) for 30min at 4°C in the dark. Cells were washed with and stored in FACS buffer (PBS with 5% FCS and 1mM EDTA) at 4°C overnight.

Instrument	All flow cytometry data was collected with a BD FACSAria Fusion (BD Biosciences)
Software	All flow cytometry data was analyzed with BD FACSDiva software (v8.0.2 and v9.0)
Cell population abundance	For the FACS DMS screen, cells were sorted based on their ENL-BFP and mCherry levels into an ENLhi fraction (30-35% of cells). Cells corresponding to at least 3,000-fold library representation were sorted per each sample replicate.<br><br>For the CRISPR-Cas9 ENL stability screen, cells were sorted based on their ENL-BFP and mCherry levels into ENLhi (5-10% of cells), ENLmid (25-50%) and ENLlow (5-10%) fractions. Cells corresponding to at least 750-fold library representation were sorted per each sample replicate.
Gating strategy	For the FACS based DMS screen, cells were strained through a 35µm nylon mesh and sorted on a BD FACSAria Fusion (BD Biosciences) operated on BD FACSDiva software (v.8.0.2) using a 70µm nozzle excluding aggregates, dead (Zombie NIR positive) and reporter negative (BFP and mCherry negative) cells.<br><br>For the CRISPR-Cas9 ENL stability screen, cells were strained through a 35µm nylon mesh and sorted on a BD FACSAria Fusion (BD Biosciences) operated on BD FACSDiva software (v.8.0.2) using a 70µm nozzle excluding aggregates, dead (Zombie NIR positive), Cas9-negative (GFP negative) and sgRNA library-negative (Thy1.1-APC negative) cells.

☐ Tick this box to confirm that a figure exemplifying the gating strategy is provided in the Supplementary Information.
